# Estrogenic Endocrine Disrupting Chemicals Influencing NRF1 Regulated Gene Networks in the Development of Complex Human Brain Diseases

**DOI:** 10.3390/ijms17122086

**Published:** 2016-12-13

**Authors:** Mark Preciados, Changwon Yoo, Deodutta Roy

**Affiliations:** 1 Department of Environmental & Occupational Health, Florida International University, Miami, FL 33199, USA; mprec001@fiu.edu; 2 Department of Biostatistics, Florida International University, Miami, FL 33199, USA; cyoo@fiu.edu

**Keywords:** estrogen, endocrine disrupting chemicals, nuclear respiratory factor 1 (NRF1), brain health

## Abstract

During the development of an individual from a single cell to prenatal stages to adolescence to adulthood and through the complete life span, humans are exposed to countless environmental and stochastic factors, including estrogenic endocrine disrupting chemicals. Brain cells and neural circuits are likely to be influenced by estrogenic endocrine disruptors (EEDs) because they strongly dependent on estrogens. In this review, we discuss both environmental, epidemiological, and experimental evidence on brain health with exposure to oral contraceptives, hormonal therapy, and EEDs such as bisphenol-A (BPA), polychlorinated biphenyls (PCBs), phthalates, and metalloestrogens, such as, arsenic, cadmium, and manganese. Also we discuss the brain health effects associated from exposure to EEDs including the promotion of neurodegeneration, protection against neurodegeneration, and involvement in various neurological deficits; changes in rearing behavior, locomotion, anxiety, learning difficulties, memory issues, and neuronal abnormalities. The effects of EEDs on the brain are varied during the entire life span and far-reaching with many different mechanisms. To understand endocrine disrupting chemicals mechanisms, we use bioinformatics, molecular, and epidemiologic approaches. Through those approaches, we learn how the effects of EEDs on the brain go beyond known mechanism to disrupt the circulatory and neural estrogen function and estrogen-mediated signaling. Effects on EEDs-modified estrogen and nuclear respiratory factor 1 (NRF1) signaling genes with exposure to natural estrogen, pharmacological estrogen-ethinyl estradiol, PCBs, phthalates, BPA, and metalloestrogens are presented here. Bioinformatics analysis of gene-EEDs interactions and brain disease associations identified hundreds of genes that were altered by exposure to estrogen, phthalate, PCBs, BPA or metalloestrogens. Many genes modified by EEDs are common targets of both 17 β-estradiol (E2) and NRF1. Some of these genes are involved with brain diseases, such as Alzheimer’s Disease (AD), Parkinson’s Disease, Huntington’s Disease, Amyotrophic Lateral Sclerosis, Autism Spectrum Disorder, and Brain Neoplasms. For example, the search of enriched pathways showed that top ten E2 interacting genes in AD—*APOE*, *APP*, *ATP5A1*, *CALM1*, *CASP3*, *GSK3B*, *IL1B*, *MAPT*, *PSEN2* and *TNF—*underlie the enrichment of the Kyoto Encyclopedia of Genes and Genomes (KEGG) AD pathway. With AD, the six E2-responsive genes are NRF1 target genes: *APBB2*, *DPYSL2*, *EIF2S1*, *ENO1*, *MAPT*, and *PAXIP1*. These genes are also responsive to the following EEDs: ethinyl estradiol (*APBB2*, *DPYSL2*, *EIF2S1*, *ENO1*, *MAPT*, and *PAXIP1*), BPA (*APBB2*, *EIF2S1*, *ENO1*, *MAPT*, and *PAXIP1*), dibutyl phthalate (DPYSL2, EIF2S1, and ENO1), diethylhexyl phthalate (*DPYSL2* and *MAPT*). To validate findings from Comparative Toxicogenomics Database (CTD) curated data, we used Bayesian network (BN) analysis on microarray data of AD patients. We observed that both gender and NRF1 were associated with AD. The female NRF1 gene network is completely different from male human AD patients. AD-associated NRF1 target genes—*APLP1*, *APP*, *GRIN1*, *GRIN2B*, *MAPT*, *PSEN2*, *PEN2*, and *IDE*—are also regulated by E2. NRF1 regulates targets genes with diverse functions, including cell growth, apoptosis/autophagy, mitochondrial biogenesis, genomic instability, neurogenesis, neuroplasticity, synaptogenesis, and senescence. By activating or repressing the genes involved in cell proliferation, growth suppression, DNA damage/repair, apoptosis/autophagy, angiogenesis, estrogen signaling, neurogenesis, synaptogenesis, and senescence, and inducing a wide range of DNA damage, genomic instability and DNA methylation and transcriptional repression, NRF1 may act as a major regulator of EEDs-induced brain health deficits. In summary, estrogenic endocrine disrupting chemicals-modified genes in brain health deficits are part of both estrogen and NRF1 signaling pathways. Our findings suggest that in addition to estrogen signaling, EEDs influencing NRF1 regulated communities of genes across genomic and epigenomic multiple networks may contribute in the development of complex chronic human brain health disorders.

## 1. Introduction

Endocrine disruptors (EDs) are defined as exogenous substances or mixtures that alter functions of the endocrine system and consequently cause adverse health effects in an organism, its progeny, or populations [1]. EDs encompass a plethora of chemicals: industrial chemicals, plastics, plasticizers, pesticides, fungicides, pharmaceutical agents, and natural chemicals found in human and animal foods [1,2,3,4]. EDs can be taken in the body through various routes of exposure: ingestion, inhalation, and dermal contact [1,2]. The main occurrence of EDs is in the environment through manufacturing and the production of e-waste. In addition to affecting the individual organism, EDs have been shown to affect children and subsequent generations [1]. EDs affect all sensitive periods in a human lifetime: gestation, childhood, puberty, reproductive life, and old age [1]. A subset of EDs, estrogenic endocrine disruptors (EEDs), specifically affect processes in the body that are influenced and modulated by the estrogen hormones and encompass the same chemical classes and routes of exposure as EEDs [1,2,3,4].

The human brain, a jelly-like mass of tissue weighing around 1.4 kg (3 pounds), is the most complex biological structure in the universe. Each individual brain originates from a single neural stem cell, which becomes approximately 100 billion neurons at birth. During some stage of the embryo’s development, 250,000 to 500,000 neurons per minute are produced. We also now know new brain cells are being generated throughout our lives—a process called neurogenesis. The brain has bursts of growth and then periods of consolidation, when excess connections are pruned. The most bursts are observed in the first two or three years of life, during puberty, and also a final burst in young adulthood. All these processes are strongly dependent on estrogens [5]. How the brain ages or become susceptible to stressors depends on genes and the environment. Adult human brain contains around 86 billion neurons, and close to 85 billion glial cells, which are as important as neurons, that amplify neural signals. Each cell contains approximately 25–30,000 genes that code for proteins which perform most life functions. Both formation and function of a protein is controlled by inter-, intra-, and extra- cellular environment within a brain tissue of a human. With estrogen having an important role in brain development, there has been a strong interest in investigating effects of EEDs on brain because their effects on circulatory and neural estrogen functions have adverse effects on brain health. There is a general agreement that human populations are constantly exposed to a wide variety of EEDs. Brain cells and neural circuits are likely to be influenced by environmental stressors that includes EEDs, because they are strongly dependent on estrogens [6]. For those reasons, the brain is very susceptible to the effects of EEDs during critical development periods. Findings from experimental models, clinical observations, and epidemiological studies converge to implicate exposure to EEDs as a significant brain health concern to children as well as adults, because certain EEDs are able to alter neurogenesis, neural transmission, and the formation of neural networks, thus implicating EEDs with an increase in neurological disorders, including autism, attention deficit and hyperactivity disorder as well as learning disabilities and aggressiveness [1,6]. EEDs have been implicated as one of the causes of neurodegenerative conditions and diseases [1,6,7]. Research suggests maternal exposure to EEDs produce various neurological deficits: changes in rearing behavior, locomotion, anxiety, learning difficulties, memory issues, and neuronal abnormalities [1,6,7].

There is emerging consensus that susceptibility to many of the complex and chronic brain diseases that individuals develop occur as a result of multiple interactions between biologically unique and inherited individual DNA sequence variation, epigenetic individuality, and genetic and epigenetic variation. Exposure to environmental and stochastic factors occur in both germ and somatic cells during intrauterine, postnatal, childhood, and adult life. Low DNA sequence variation across unrelated individuals and our closest related species are not sufficient enough to account for all major differences in physiological and chronic brain disease phenotypic outcomes. Nuclear respiratory factor 1 (NRF1) is one of the sequence-specific DNA-binding redox sensitive transcription factors that constitutes the most important and diverse gene-regulatory mechanism. Context specific NRF1 proteins binding to DNA response elements depends on the variation in DNA binding sequences and the local cell-dependent chromatin alterations, including histone modification, DNA methylation status, and accessibility of regulatory elements [8]. Recently, Satoh et al. reported 2470 NRF1 target genes, including many estrogen signaling pathway genes, associated with neurodegenerative diseases [9]. Both neurogenesis and synaptogenesis are controlled by NRF1 regulated genes. A strong association of NRF1 with human fetal brain and neural development has been recently reported [10]. The effects of EEDs on the brain are varied during the entire life span and far-reaching with many different mechanisms. How a healthy brain responds to EED stressors may help to explain how these stressors disrupt brain cells to produce undesired lesion producing brain diseases. The recent advances in bioinformatics and molecular genetics have provided an opportunity to understand how genes, and genetic and epigenetic changes interact with environmental and stochastic stressors to either preserve health or cause disease [4]. Keeping in mind the above concepts, first, we reviewed environmental epidemiologic and experimental evidence on brain health with exposure to endogenous estrogen, oral contraceptives, hormonal therapy and then EED compounds such as BPA, PCBs, phthalates, and metalloestrogens: arsenic, cadmium, and manganese. Using bioinformatics, molecular and epidemiologic approaches, we have attempted to review how these ubiquitous EEDs, which are known to disrupt the ovarian and local estrogen function, influence NRF1 transcription factor regulated gene networks, contribute in the development of complex chronic human brain health disorders.

## 2. Epidemiologic and Experimental Evidence of Brain Health Deficits with Exposure to Estrogenic Endocrine Disruptors (EEDs)

Table 1, Table 2 and Table 3 present epidemiologic and experimental evidence on brain health with exposure to endogenous estrogen, oral contraceptive, hormonal therapy and EED compounds, such as, BPA, PCBs, phthalates, and heavy metals-arsenic, cadmium, and manganese. We have succinctly described below effects of each estrogenic chemical on brain health.

### 2.1. Estrogens and Brain Health

Estrogen has a complex role in the body, and been tied to cardiovascular, metabolic, endocrine, reproductive, and neurodegenerative diseases, as well as roles in gene expression and protein synthesis [11,12]. Estrogen has been shown to have a role in sexual differentiation in the brain [12], neuroprotection, and anti-inflammatory effects [13,14,15,16,17,18]. Studies also suggest estrogen plays a significant role in the modulation of neuroplasticity and synaptogenesis [19,20,21,22,23,24]. Estrogens have also been demonstrated to protect and regulate mitochondrial function in the brain [25,26,27].

When assessing the effects of endogenous estrogens on brain health, we observed endogenous estrogen levels were quantified through blood and/or serum levels, or through the use of a surrogate of exposure to estrogen. The surrogates take into account the times at which the human female body is exposed in a lifetime, from the start of menarche to the end of menopause. We examined studies that took into account exogenous estrogen use through either controlling it as a confounder, or using it as an exclusionary criterion, since exogenous estrogen can modify the effects of endogenous estrogens.

Our review identified cross-sectional epidemiological studies that assessed the effects of endogenous estrogen on brain health [28,29,30,31,32]. Four of the studies examined the relationship between endogenous estrogen and memory performance [28,30,31,32] and one study examined the relationship between age of Parkinson’s disease (PD) onset and exposure to endogenous estrogen [29]. One study examined the effects of endogenous estrogens in males [32], and the other four studies examined the effects on females [28,29,30,31].

A study of 393 postmenopausal women did not find any significant associations between endogenous estradiol levels and neuropsychological test performance [28]. Another study did not find any significant associations between reproductive time as a surrogate of endogenous estrogen exposure and performance on cognitive and memory tests in a cross-sectional study of 760 postmenopausal women [31]. However, a study by Heys et al., found significant associations between proxies of endogenous estrogen exposure and cognitive delayed recall scores (*p*-value = 0.001, 95% CI, 0.008–0.02) and the mini-mental state exam scores (*p*-value < 0.001, 95% CI, 0.004–0.007) in a cross-sectional study of 11,094 postmenopausal women [30]. In a cross-sectional study of 181 older men, examining the effects of endogenous estrogen exposure from serum levels and verbal memory assessment scores in men, higher total levels of estradiol were associated with better performance (β = 0.17, *p*-value < 0.02) [32]. Cereda et al., found that age of PD onset was positively associated with the duration of exposure to endogenous estrogens using reproductive surrogates of endogenous estrogen exposure (7.10, [3.31], *p*-value = 0.032) in a cross-sectional study of 579 females [29].

We identified case-control studies that assessed the effects of endogenous estrogen exposure on brain health [33,34], with one study assessing endogenous estrogen exposure and Alzheimer’s disease (AD) risk using serum estrogen levels [33] and one study assessing amyotrophic lateral sclerosis (ALS) risk using a lifetime estrogen exposure surrogate [34]. In a case-control study of 50 AD cases and 93 controls, Manly et al., found women with the lowest endogenous estradiol levels were 4 times as likely to have AD compared to women with the highest estradiol levels (OR = 4.2, 95% CI 1.1–15.6) [33]. In another case-control study of 131 ALS cases and 430 age-matched controls, De Jong et al. found longer reproductive time-span decrease ALS risk (OR = 0.95, 95% CI 0.91–0.98) and longer endogenous estrogen exposure decreases ALS risk (OR = 0.95, 95% CI 0.89–1.01) [34].

We identified cohort studies that assessed the effects of endogenous estrogen exposure on brain health [35,36,37]. One study assessed dementia risk and the length of the reproductive period as a surrogate for endogenous estrogen exposure [35], and two studies assessed AD risk, one using bioavailable estrogen levels [36], and one using a surrogate measure of endogenous estrogen exposure [37]. Geerlings et al. found after adjusting for covariates, women with longer reproductive spans had an increased risk of dementia compared to women with the shortest reproductive spans (RR = 1.78, 95% CI 1.12–2.84) and an increased risk of AD (RR = 1.51, 95% CI 0.91–2.50) in a cohort of 3601 postmenopausal women [35]. The risk was more pronounced in carriers of the APOE allele [35]. In a cohort study of 119 women with Down’s syndrome, Schupf et al. found women with low bioavailable estrogen were more likely to develop AD (HR = 4.1, 95% CI 1.2–13.9) [36]. In another cohort study of 133 postmenopausal females, Fox et al. found longer duration of months with endogenous estrogen exposure may have a protective effect against AD risk (*p*-value = 0.0235, 96% CI 0.9907–0.9993) [37]. The effect is more pronounced when subjects were over the total median number of months (*p*-value = 0.00754, 96% CI 0.004118–0.005289) [37].

### 2.2. Oral Contraceptives and Brain Health

Oral contraceptives (OC) containing specifically ethinyl estradiol has been shown to reduce the amount of available endogenous estrogen in the body [38]. OCs has varying effects brain structure, function, and cognition [39]. Most recent studies have shown varying effects, However, most recent studies do not differentiate between the various types of contraceptives, making it difficult to find out, if, the contraceptives used contained ethinyl estradiol and inconsistencies are found in the reporting of the type of OC used [40].

A cross-sectional study examined OC effects on spatial and verbal abilities found OC users to perform better on some cognitive test, but not others and between homogenous groups of specific OC medications [40]. Another cross-sectional study by Egan and Gleason found OC users to have better performance on cognitive exams compared to non-users [41]. Griksiene and Ruksenas found OC to negatively affect cognition [38]. A review by Warren et al., suggest an overall positive effect with OC use and verbal memory [42].

### 2.3. Hormonal Replacement Therapy and Brain Health

Hormone replacement therapy (HRT) has been shown to be associated with the onset of neurodegenerative disease, although evidence points to a timeframe dependent response, depending on when the therapy is initiated. As summarized in Maki and Henderson, initial observation studies and analyses with women indicated the use of HRT containing estrogen to be associated with a reduced risk of AD [43]. Studies suggest that the timing of HRT during parts of the menopausal stage may dictate whether beneficial adverse effects are observed [44].

Several analytical studies, however, showed associations between the use of HRT and neurodegenerative disease. In the Women’s Health Initiative Memory Study (WHIMS), a randomized, double-blind, placebo-controlled clinical trial, it was found that an HRT treatment of estrogen plus progestin increased the risk for dementia in postmenopausal women and did not prevent cognitive impairment [45]. In another study from the same trial, it was found that estrogen-only HRT did not reduce the incidence of dementia, or cognitive impairment, and increased the risk for both [46].

An ancillary to the WHIMS study, the Women’s Health Initiative Study of Cognitive Aging, or WHISCA, further supports HRT’s association with neurodegenerative disease [47], however, results varied. One finding from Resnick et al. (2006), from the WHISCA study found that a combination of conjugated equine estrogen with medroxyprogesterone acetate (estrogen + progestin) appeared to negatively impact verbal memory, but positively affect figural memory among postmenopausal women, free of probable dementia, and compared to controls [47]. Another finding from Resnick et al. (2009), showed estrogen alone, as conjugated equine estrogen, did not improve cognitive functioning and lowered certain cognitive functions in women with prior hysterectomy [48]. Other studies have indicated that no visible improvement has been observed in using estrogen only treatments for cognitive function [49,50,51,52]. Other studies that administered estrogen only treatments to younger surgically menopausal women have shown benefits to memory [53,54], which may show an age-related effect of estrogen only treatments.

New studies and reviews give mixed results. A newer observational study conducted by Shao et al. (2012), showed increased AD risk amongst women who used HRT more than five years after menopause, but observed a decreased risk of AD if used within five years of menopause [55]. Another recent meta-analysis showed no observable associated between postmenopausal HRT use and AD or dementia [56]. While another study found that an increased risk in the type of hormonal therapy used and PD risk [57]. At least one study has examined the relationship between HRT use and brain cancer, where a positive association between HRT use and the diagnosis of meningioma was observed [58]. In summary, to date, most of the neuroprotective actions of E2 against AD are based on animal models. While animal studies support a beneficial role for E2 in AD, there has been little success in translating this preclinical work to treat AD with estrogens. This may be as a result of species and sex differences, and brain complexity. HRT and anti-estrogen therapy (raloxifene) in AD are focused on circulating estrogens, as measured by blood levels, not based on brain estrogen levels. This is one of the main reasons why endocrine therapies (HRT and anti-estrogen therapy) are unable to produce a significant beneficial effect in human AD [59]. Also there are negative effects of E2-based therapies, such increased risk of cancer and loss of gray matter in certain regions of brain in older women. 

### 2.4. Bisphenol A and Brain Health

BPA, an EED, is widely used to make polycarbonate plastics and resin [4,60]. BPA can be found in many consumer products that contain plastics [60]. These include water and baby bottles, compact discs, impact-resistant safety equipment, medical devices, food cans, bottle tops, water supply pipes, ATM receipts, dental sealants and composites [60]. Inhalation, ingestion, and dermal contact are all routes of exposures for BPA [60]. Additionally, food containers made with BPA can cause BPA to leech into foods and is expedited by the use of high heat [60]. It is of note that during the 2003–2004 NHANES cycle, BPA was detected in 93% of urine samples from people 6 years of age and older [61]. BPA has also been demonstrated to show effects at all stages from prenatal to old age [62]. There is evidence to suggest BPA is released from plastic bottles used to store beverages consumed by consumers, with BPA migration increasing with exposure to heat [63]. Animal studies have indicated the ability BPA to penetrate the blood brain barrier due to its lipophilic nature [64,65].

Numerous studies highlight the link between BPA and neurotoxicity. Synaptogenesis, the formation of synapses between neurons [66], and neurogenesis, the formation of new neurons [67], are processes by which the brain can regenerate itself from damage due to trauma and disease. Several animal studies have BPA to significantly affect these processes. Decreased synaptic spinal density and adverse synaptic structures were observed in both male and female species of animals which include rodents [68,69,70,71,72,73] and primates [74]. Postnatal exposure to BPA was also shown to affect synaptic spinal density and structure in a rodent study [71]. BPA exposure was also shown to decrease neurogenesis in the hippocampus of postnatal female rats whose mothers were exposed to BPA [75] as well as young adult mice [76]. Recently, it has been shown that both BPA and bisphenol S (BPS), a replacement used in BPA-free products, at a very low dose, equally affects neurodevelopment by altering neurogenesis [77]. Some studies have indicated BPA has a neuroprotective effect on brain cells. In a study using an immortalized clonal mouse hippocampal cell line (HT-22), BPA was found to protect against glutamate and β amyloid protein induced cell death [78]. Some studies have also indicated that BPA has no effect on neurotoxicity. In a study examining dietary exposure to F1 rat offspring, BPA was shown to have no effect on offspring neurological development and motor activity [79].

BPA has been found to cause hyperactivity in rats, compared to its derivatives, possibly due to the longer-lasting effect of the parent compound in the brain [80]. A single dose of BPA administered to neonatal mice was observed to affect cognitive function and alter adult spontaneous behavior, with the effect being seen in mid and high dosed mice [81]. In utero exposure to BPA in mice was shown to reduce dendritic spine densities in the hippocampal CA1 region, regardless of its dose [82]. BPA was demonstrated to impair neural ectoderm specification and neural progenitor cells in mouse embryonic stem cells [83]. In a study using *Caenorhabditis elegans* to examine early embryogenesis exposure to BPA and BPS into adulthood, it was shown that changes in behavior and learning were followed into adulthood [84]. BPA was shown to decrease the proliferation of multipotent neural progenitor cells and produce cytotoxicity in F1 mice, and in low-doses stimulated neuronal differentiation which might disrupt brain development [85].

Animal studies have indicated BPA to affect various aspects of memory at lower than the US EPA’s reference safe daily limit of 50 µg/kg/day [86]. The types of memory affected include spatial memory, visual memory, object recognition, working memory, reference memory and navigational memory [68,69,70,71,73,74,75,76,87,88]. Animal studies have also indicate affects to locomotor function [71,87].

Prenatal BPA exposure has been shown to produce more aggressive and hyperactive behavior in offspring when compared to mothers with lower BPA levels [89]. This human study is consistent with animal studies that have also shown that prenatal BPA exposure is associated with increased aggression, alterations in the dopaminergic system, and other neurobehavioral effects [90,91,92,93,94,95,96,97]. In a study examining early life exposure to BPA, it was found that prenatal urinary BPA concentrations in the mother and child were associated with anxiety, depression, and hyperactivity [98]. A recent French study of 46 children with autistic spectrum disorders (ASD) and 52 controls reported an association between BPA exposure and ASD in children [99]. In autistic children, plasma levels of BPA and phthalates were significantly higher compared to controls [100]. Studies suggest BPA may cause autism by inducing methylation changes in transcriptionally relevant regions of the BDNF gene in the hippocampus of mice [101]. One study using cross-sectional data from the Canadian Health Measures Survey found children taking psychotropic medications was associated with urinary BPA (OR 1.59; 95% CI 1.05–2.40) [102]. Another study assessing prenatal exposure to BPA and phthalates and infant neurobehavior at 5 weeks found no associations with BPA and some associations with phthalate exposure and improved possible neurobehavior [103]. In a prospective cohort study following African-American and Dominican women from pregnancy to children’s age of 7–9, it was found that high prenatal BPA concentrations was associated with increased internalizing and externalizing behaviors in boys with a decrease in internalizing behavior in girls and high postnatal BPA concentrations was associated with increased internalizing and externalizing behaviors in girls than in boys [104]. Other studies have found a decrease in hyperactivity symptoms in boys and an increase in anxiety, depression, and externalizing behavior in young girls [89,105]. Other studies have found no associations among maternal BPA levels, behavior, and autism [106].

Studies have demonstrated evidence of BPA exposure and associations with brain cancer. In particular, meningioma, which account for 20% of intracranial tumors, may be linked to estrogen sensitivity and genetic predisposition [107]. BPA exposure has also been implicated to be associated with increased risk of meningioma—a common type of brain tumor—and patients with the highest urine BPA levels were about 1.4 to 1.6 times more likely to have meningioma compared to those with lower concentrations [108]. BPA at high doses has also been shown to induce cell growth and the production of prolactin in a pituitary tumor cell line [109]. BPA increases *N*-ras expression in a K. marmortatus model, which shows exposure to BPA can predispose the host to environmental carcinogenesis [110]. BPA may produce nongenomic estrogenic responses in pituitary tumor cells by detectable ERK phosphorylation [111]. In rat pituitary GH3 cancer cell lines, BPA interferes with cell proliferation alone or with co-treatment with T3 [112].

In summary, besides diethylstilbestrol (DES), BPA is one of the widely researched EEDs. BPA is detected in many human fluids and tissues including saliva, serum, urine, amniotic fluid, follicular fluid, placental tissue and breast milk [99]. From the above studies, it is apparent that BPA exposure has a great potential to impact human brain development and possibly contributes to the increasing prevalence of human neurodevelopmental and behavioral disorders (Table 1, Table 2 and Table 3).

### 2.5. Phthalates and Brain Health

Phthalates, a group of endocrine-disrupting chemicals commonly used to [4], are used as in plastics to make them flexible and resilient, also known as plasticizers [113,114,115,116]. Di(2-ethylhexyl) phthalate, or DEHP is the one of the most widely used plasticizers and is found plastic products such as wall coverings, tablecloths, floor tiles, furniture upholstery, shower curtains, garden hoses, swimming pool liners, rainwear, baby pants, dolls, some toys, shoes, automobile upholstery and tops, packaging film and sheets, sheathing for wire and cable, medical tubing, and blood storage bags [114,117]. Other commonly used plasticizers are di-*N*-butyl phthalate, found in carpets, paints, glue, insect repellents, hair spray, nail polish, and rocket fuel [115]; di-*N*-octyl phthalate (DNOP), found in carpet back coating, packaging films, medical tubing and blood storage bags, floor tile, wire, cables, adhesives, cosmetics, and pesticides [113]; and Diethyl phthalate, in found in products such as toothbrushes, automobile parts, tools, toys, food packaging, cosmetics, insecticides, and aspirin [116]. Phthalates are not part of the chemical chain that make up the polymers in plastic, allowing them to readily released into the surrounding environment [113,114,115,116]. Exposure to phthalates comes through ingestion, inhalation, and, to a lesser extent, dermal contact with phthalate-containing products [117]. Phthalates have also been demonstrated to have the ability to cross the blood brain barrier [118,119].

Phthalates have been found to disrupt neuroplasticity. A recent studies using a rat model suggests exposure to phthalates reduces dendritic spine density, neurogenesis, and synaptogenesis in male rats [120]. A recent review of phthalates and neuroplasticity also suggests phthalates negative effects on neurogenesis and plasticity in animal models [121,122].

Phthalate exposure has been associated with various cognitive deficits. Animal studies indicate that specific learning deficits include decreased fear conditioning [123], impaired spatial memory [72,122,124] and adverse effects to locomotor activity [72]. A cross-sectional epidemiological study using data from the NHANES 2011–2012 data collection found individuals who had difficulties thinking and remembering had increasing levels of urinary phthalates along with other estrogenic chemicals [125]. In another study found prenatal maternal exposure to phthalates was inversely associates with child IQ DnBP and DiBP (*b* = −2.69, 95% CI −4.33 to −1.05 and *b* = −2.69, 95% CI −4.22 to −1.16) [126].

There are a group of studies that have found possible links between phthalates and brain behavioral disorders such as ASD and attention deficit hyperactive disorder (ADHD). One study, examining exposure to estrogenic endocrine disrupting chemicals (EEDCs) and autism and ADHD found intracranial exposure to several classes of EEDCs, which include phthalates, caused significant hyperactivity in neonatal rats [127]. Altered gene expression was also observed [127]. A retrospective study followed children 1–6 years of age with a follow-up at 6–8 years of age and found the children with exposure to phthalates from PVC pipe dust were more likely to develop ASD [128]. Additionally, two other studies looked at both BPA and phthalate levels and neurobehavioral outcomes, with associations found between adverse neurobehavioral outcomes and increasing urinary phthalate levels [103,106]

Phthalates are suspected in the development of neurodegenerative diseases [119]. In an animal study involving a rat model, phthalate exposure was shown to impair cognitive function and increase the level of phspho-Tau, a precursor of AD development, in exposed rat offspring [129]. Currently no definitive epidemiological studies have been conducted with phthalates and neurodegenerative diseases.

### 2.6. Polychlorinated Biphenyls and Brain Health

PCBs are synthetic organic chemicals composed in different combinations of 209 different chlorinated compounds, or congeners [130]. PCBs have been used as coolant and lubricants in transformers, and its manufacture was banned in 1977 because of harmful health effects, however, old products may still contain them [130]. Many of PCBs congeners produce estrogenic effects [4]. PCBs have been found to cause cancer in animals [130]. Routes of exposure included occupational exposure, breathing air near contaminated areas, using older products manufactured before and around 1977, and eating contaminated food [130]. PCBs bio accumulate in the food chain and remain active in the environment for extended periods of time [130].

Recent animal and cell studies have shown PCBs to adversely affect brain health. Animal studies have shown exposure to PCBs affect brain health by affecting social behavior [131], increased hyperactivity [132], reduction in learning ability [133], and physiological effects on the brain such as neuronal degradation [132,134], neuronal loss and damage [135], and susceptibility to amyloid stress and reduced expression of synaptic proteins [134]. In in-vitro models, PCBs have shown to cause neuronal cell death [136] and interfere with estrogen’s neuroprotective effects on neurons and brain cells [137]. In a study using ovariectomized, estradiol-replaced ewes, the VEGF/VEGFR systems is affected by PCBs, which therefore affect the production of cerebrospinal fluid [138].

In a nested case-control study in Finland, serum PCBs were not associated with PD development [139]. In a retrospective mortality study examining PCB exposure amongst workers in capacitor plants, sex-specific analyses found higher deaths from PD amongst exposed women [140]. In a case-control study examining post-mortem brain tissue from PD, AD, and control patients, PCB levels were higher in PD groups, and when stratified by age, were higher amongst women [141].

There are a number of studies that support the hypothesis that exposure to EEDs represent risk factors for ADHD. Lead and PCB exposures have been implicated with ADHD in children [142]. There are a large number of studies focused on the association between exposures to PCBs and cognitive function. There is ample evidence in human populations to suggest both high and low exposures to PCBs during fetal development may be linked to cognitive deficits. Also alterations in sexually dimorphic behaviors have been reported in humans exposed to PCBs. For example, cross-sectional studies have shown the effects of PCBs on brain health. In children, PCB exposure is associated with lower IQ [143] and lower visual memory function [144]. In older populations, PCB exposure is associated with lower verbal learning and memory [145], lower verbal memory and depressive symptoms [146], lower score on memory and learning measures [147] and attenuation of emotional wellbeing and attentional functioning [148].

### 2.7. Cadmium and Brain Health

Cadmium is a naturally occurring metal in the earth’s crust and a natural constituent of ocean water. Populations are exposed through food, cigarettes, smoke, drinking water, and air. Cadmium is introduced into the food chain through soil and food contact surfaces, and through foods such as leafy vegetables, grains, legumes, and organ meats. Occupation exposures are highest in occupations involving cadmium-containing products, and occupations involved in alloy, battery, plastics, and coloring production. Current evidence supports cadmium being cytotoxic at high concentrations. At low doses cadmium mimics estrogen (E2) effects both in vitro and in vivo [149].

Cell models have shown cadmium to have adverse effects on brain health. In a study using hippocampal slides of CA1 neurons, cadmium was shown to affect synaptic transmission and short-term neural plasticity [150]. In another cell study using PC12 and SH-SY5Y cells, cadmium induced cell apoptosis [151,152]. Animal models have shown cadmium to affect different aspects of brain health. In a zebrafish animal model, cadmium has been shown to inhibit neurogenesis in embryonic development [153]. Cadmium has been shown to negatively affect brain function. Cadmium has been shown to induce apoptosis in vitro in rat cerebellum cortical neurons by affecting by affecting calcium homeostasis [154] and also has been shown to induce apoptosis in vitro in through damage of mitochondria in rat oligodendrocytes [155]. Cadmium has been demonstrated to interact with β amyloid peptides which may lead to the development of AD [156].

Studies on human populations have also shown cadmium’s adverse effects on adult brain function. A cross-sectional study using NHANES III data from 1988 to 1994 compared neurocognitive test scores and urinary cadmium concentrations found individuals with no smoking history, or known occupational cadmium exposure were found to have lower attention/perception scores with increasing urinary cadmium levels [157]. In a cohort study of rural elderly Chinese persons, it was found that increasing serum cadmium and copper levels was significantly associated with lower composite cognitive scores [158]. Another study, which examined the cerebrospinal fluid of ALS patients, found elevated levels of various metals, including cadmium, that were higher than the measured blood levels and indicative of bioaccumulation [159]. A case-control study examining heavy metal levels in a group of Mongolian people found elevated cadmium, as well as other heavy metals in those with Parkinson-like symptoms from their hair samples [160]. In a study of boiler workers and occupational exposure, exposure to various heavy metals, including cadmium was associated with neurodegenerative-like conditions similar to PD and AD [161].

### 2.8. Arsenic and Brain Health

At low doses arsenic acts as a xenoestrogen and modifies estrogen signaling [149]. Human populations are usually exposed through the air, drinking water, and food, with food being the main source of arsenic in a population, with some areas having naturally high levels of arsenic [162]. Occupational exposure occurs through individuals working in metal smelting, wood treatment, and those working in the production and application of pesticides and herbicides [162]. Arsenic is also used in the animal and poultry feed as an antimicrobial additive [162]. The major routes of exposure are inhalation and oral, with dermal exposure being considered a minor route [162]. Arsenic has been associated with various health conditions, which include respiratory disorders, cardiovascular outcomes, diabetes, ocular effects, immune response disturbances, impaired neurological function, developmental effects, and cancers [163]. Genetic polymorphisms are suspected of contributing to the sensitivity towards arsenic [163].

In animal studies using rats, arsenic exposure through ingest water has been shown to impair neurogenesis, worsened spatial memory, and promote abnormal neural synapses. In addition, animal studies in rats have shown arsenic exposure to affect synaptic plasticity, by affecting the expression of NDMA receptors [164,165] and downregulating the PTEN-Akt-Creb signaling pathway and damaging cerebral neurons [166]. In a cell study conducted by Shavali and Sens (2007), arsenic was found to cause enhanced oxidative stress and cell death in cultured neuronal cells, when administered with dopamine [167]. In a study using a cholinergic neuronal cell line overexpressing amyloid precursor protein (APP) and exposing it to sodium arsenite and its metabolite, dimethylarsenic acid, it was found to affect cleavage of APP and increase its production [168].

In a cohort study consisting of 133 men and 201 women from the Project FRONTIER, a rural healthcare study, long-term low level exposure to arsenic from groundwater was found to be associated with poorer scores in language, visuospatial skills, and executive functioning, global cognition, processing speed, and immediate memory [169]. In another study from Project Frontier, consisting of 526 subjects genotyped according to the AS3MT gene, exposure to higher low level arsenic in groundwater reduced cognitive functioning, but the results differed with amongst the different SNPs [170]. In a case-control study measuring heavy metal serum levels in 89 Ad patients and 188 cognitively normal controls, there was no difference in serum arsenic levels between the AD group and controls [171]. Another study conducted by Edwards et al., with a cohort consisting of 733 AD patients, 127 individuals with mild cognitive impairment, and 530 individuals of normal cognition, found that exposure to low level arsenic exposure from groundwater was found to be associated with poorer neuropsychological performance [172]. A cross-sectional study by Shuie (2015), using NHANES 2011-2012 data, found that individuals who has problems remembering had higher levels of urinary arsenic [125].

### 2.9. Manganese and Brain Health

Manganese (Mn) is a naturally abundant environmental trace mineral that is an essential nutrient required as a cofactor for various enzymes and is found naturally in grains and fruit. Manganese, which is found in various industrial processes and products, exposure can occur through inhalation, oral, dermal, and occupational routes. Recently, prepubertal exposure to environmental levels of Mn has been shown to produce precocious mammary gland development through inducing gonadotropin-releasing hormone (GnRH) regulated increases in levels of serum LH and E2 by activating the hypothalamic-pituitary-ovarian axis [173]. MnCl2 (1.0 µM) is almost 9% as efficient as 17-β estradiol in activating estrogen receptor-mediated transcriptional activity in MCF-7 breast cancer cells. Mn has been show to accumulate in the hypothalamus and induces precocious puberty in rats through activating GnRH peptide release from the basal hypothalamus leading to elevated puberty-related hormones including E2 [173].

Evidence suggests manganese having a role in neurotoxicity. Chronic manganese exposure has been shown to promote the build-up of the metal in the basal ganglia, white matter, and cortical structures of the brain [174]. Manganese has also been shown to cause an inhibitory effect on NMDA receptors [175]. Although manganese is an essential nutrient and has beneficial uses in the human body, increased levels of manganese in the body can lead to PD-like symptoms and developmental exposure has been shown to negatively affect neurological development [176]. Manganese appears to interfere with dopaminergic synaptic transmission, by possibly impairing presynaptic dopamine release. A study using a monkey model showed manganese exposure caused neurotoxicity by inhibiting dopamine neurotransmission [177].

In animals chronic exposure of Mn has been shown to produce subtle deficits in spatial working memory, spontaneous activity, manual dexterity, and compulsive-like behavior [178]. A small cohort study that followed 26 welders exposed to manganese found after a 3.5-year follow-up found worsened olfactory, extrapyramidal, and mood disturbances [179]. A cohort study following asymptomatic welder trainees with no previous manganese exposure found low-level exposure to cause sub-clinical brain changes in subjects before any measurable learning deficits occur [180]. In another cross-sectional study assessing children’s intellectual functioning and arsenic and manganese exposure, blood manganese levels were negatively associated with full scale IQ test scores (*p*-value < 0.05), working memory (*p*-value < 0.05), and perceptual memory (*p*-value < 0.05) [181]. A cross-sectional study by Kim et al. [182] examining low-level manganese exposure in adults of an Ohio community found subtle subclinical effects in unified PD rating scale and postural sway test for PD. A cross-sectional study of school-aged children in Brazil found inverse scores on executive function and attention tests with manganese levels [183]. A study amongst school children in Canada found low-level manganese exposure in drinking water was associated with poorer neurobehavioral functions [184].

Manganese has been shown to produce Parkinsonism [176,185]. Recent epidemiological studies have assessed the effects of manganese on brain health. A cross-sectional study by Hozumi et al. [186], analyzed the cerebrospinal fluid of various neurodegenerative disease patients and found a higher level of manganese among PD patients (*p*-value < 0.05). Koc et al. [187] found higher levels of metal, including manganese, in hair samples of AD patients compared to controls. A case-control study Miyake et al. [188], assessing dietary intake of heavy metals amongst PD patients found no association with manganese intake. A case-control study by Roos et al. [159] found elevated manganese levels in cerebrospinal fluid of ALS patients. A study by Kihira et al. [189] found elevated manganese levels in ALS patients vs controls from their hair samples. Another study by Arain et al. [190] found higher levels of manganese and aluminum in hair samples of patients suffering from neurodegenerative disease.

In summary, experimental and epidemiologic studies suggest that exposure to metalloestrogens—Cd, As and Mn may influence brain health and they may be associated in the development of some of the chronic complex brain disorders.

## 3. Mechanisms of Actions of Estrogenic Endocrine Disruptors (EEDs) on Brain Health Deficits

**Figure 1:**
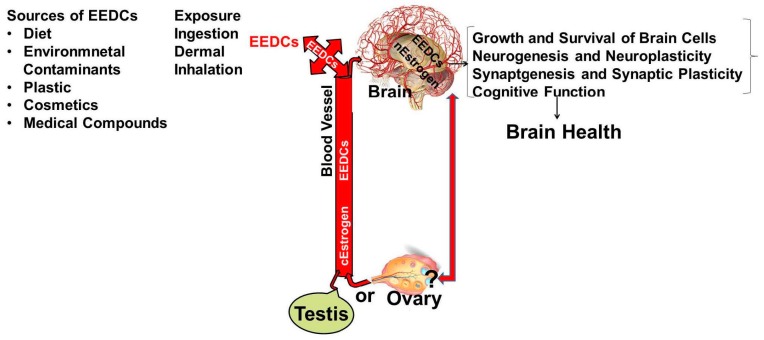
A schematic representation showing how circulatory (c) estrogen produced by ovary or testis, neural (n) estrogen (produced by aromatization of testosterone to 17 β estradiol (E2) by neuronal or glial aromatase in the brain) and estrogenic Endocrine Disruptors (EEDs) entering into the blood circulation from ingestion, inhalation or dermal exposure may interact in the brain and their impact on brain health. Neural estradiol and ovarian estradiol feedback remains to be ascertained.

**Figure 2:**
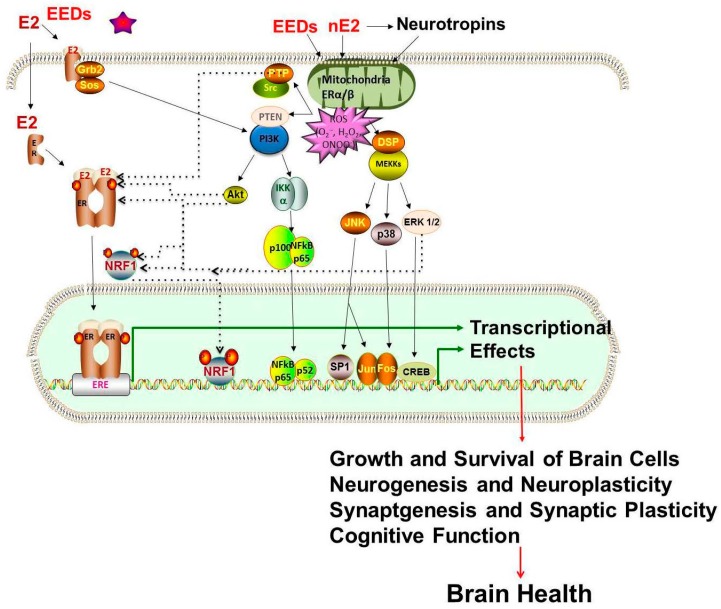
Possible mechanisms of estrogen or EED action. Genomic mechanism involves the nuclear form of estrogen receptors ER-α or ER-β which associates with either the estrogen response element (ERE) or Fos/Jun heterodimers that bind, in turn to activator protein-1 (AP-1) sites, whereas plasma membrane bound ER-mediated mechanisms include the activation of an ER linked to the phosphatidylinositol 3-kinase (PI3-K)/Akt, ERK1/2 mitogen-activated protein kinase (MAPK) pathways, protein kinase C (PKC), and cAMP/protein kinase A (PKA) converging with the genomic pathway. Actions of neurotrophins are influenced by estrogens or EEDs and vice versa. These pathways are also activated by reactive oxygen species (ROS). ROS mediated redox signaling leading to the activation of Akt/ERK/PKC pathways modulates nuclear transcription factors (e.g., ER, CREB, c-Fos/c-Jun, NRF1) binding to their DNA motifs in the regulatory regions in DNA sequences of genes associated with growth and survival of brain cells, neurogenesis and neuroplasticity, synaptogenesis and synaptic plasticity and cognitive function. Estrogenic effects mediated through these pathways are inhibited by antioxidants.

Estrogenic Mechanisms: It is clear through the studies described above that physiologic and pharmacologic forms of estrogen and estrogenic active environmental chemicals affect brain health (Table 1, Table 2 and Table 3). Estrogens have long been implicated in influencing brain health disorders, yet the molecular mechanisms underlying brain health effects remain unclear. Estrogens are very intriguing complex chemicals that regulate many of the women and men body’s functions, including development, growth, reproduction, and nervous and immune systems, as well as the way various organs operate. In addition to the primarily produced in ovary in females, estrogens are also produced by many other tissues such as the testis, liver, adrenal glands, brain, breasts and fat cells. For example, estrogen in the testis is produced by Leydig cells and germ cells by aromatization of testosterone. A relatively high concentration of estrogen in rete testis fluid and in semen of several species that exceed even the female vasculature is observed. 17 β estradiol concentration in testis venous blood and lymph is relatively high in all species [191]. Similarly, in brain, both neurons and glia synthesize E2 through aromatization of testosterone. The neurons of hypothalamus of rhesus monkeys has been shown to produce large amounts of estradiol to regulate release of gonadotropin releasing hormone (GnRH) [192]. Recently estrogen synthesized in glial cells in Zebra Finch brain under conditions of brain injury have been reported to control inflammation [193]. Associations between gender specific estrogen levels and brain functions-related health challenges, such as cognition, depression, brain neurodegenerative diseases such as AD and PD and neurodevelopment disorders, such as autism, have raised the importance of estrogens in brain health. It has become apparent that estrogens play very important role in brain health of both sexes. The underlying mechanisms of brain susceptibility to estrogen’s effect remain elusive. In both cell culture and animal studies, estrogen exposure has been shown to have adverse as well as protective effects on neurons, through genomic and non-genomic pathways [194]. Endogenous estrogens play a role in the growth and regulation of neurons and synaptic plasticity [19,20,21]. How circulating EDEs interact with neural and/or circulatory estrogens in the control of brain structure and function remains to be understood. Maternal PCBs exposure has been reported to accumulate in brain and liver fetal tissues that are strongly associated with PCB levels in adipose tissue [195]. Other EEDs have been also found in brain. The brain is the fattest tissue (60% fat) in our body. Therefore, once EEDs enter in the body, these compounds can get into the circulation, cross through the blood brain barrier, metabolized, and/or stored by the brain, where their effects can last for long periods of time. The biochemical and molecular mechanisms by which EEDs may affect brain health are not clear. The effects of EEDs on the brain are varied during the entire life span and far-reaching with many different mechanisms. A schematic representation showing how circulatory (c) estrogen produced by ovary or testis, neural (n) estrogen [produced by aromatization of testosterone to 17 β estradiol (E2) by neuronal or glial aromatase in the brain] and EEDs entering into the blood circulation from ingestion, inhalation or dermal exposure may interact in the brain and their impact on brain health (Figure 1). The relationship between neural estradiol and ovarian estrogen or EED feedback is not clear. However, PCBs, BPA, phthalates, Cd, As or Mn, through influencing gonadotropin-releasing hormone (GnRH) regulated increases in levels of serum LH and E2, have been shown to modify the hypothalamic–pituitary-ovarian axis and they also bind to estrogen receptors [1,2,149,173]. BPA, Phthalates and metalloestrogens—Cd, As and Mn have been shown to cross the blood brain barrier [1,64,65,118,119,149,173]. Metalloestrogens, such as arsenite, cadmium, mimic the actions of physiological estrogens through binding to estrogen receptors [196,197,198]. We have not discussed existing paradigms of actions of estrogen, BPA and phthalates, because they have been extensively reviewed. The most widely researched paradigm is that effects of estrogens and EEDs are mediated via estrogen receptors (ERa, ERβ, GPER/GPR30), all three of which are widely expressed in the brain [1,2,3,4] (Figure 2). Excess endogenous estrogens or EEDs produce adverse effects on brain health as a result of aberrant estrogen receptor signaling. In addition to the classical estrogen receptor-mediated signaling, emerging new evidence indicate other mechanisms, such as oxidative stress mediated redox signaling and epigenetic disturbances may be involved with EEDs-associated detrimental brain health outcomes.

Oxidative Stress Mechanisms: There is emerging evidence that exposure to estrogen and EEDs-PCBs, BPA, phthalate produce oxidative stress. Oxidative stress may be one of the mechanisms associated with the adverse effect of EEDs on brain health (Figure 2). Physiologically achievable concentrations of estrogen or estrogen metabolites have been shown to generate reactive oxygen species (ROS). In addition to the direct effect of estrogen on mitochondria and the redox cycling of catechol estrogen, estrogen-induced proinflammatory cytokines, such as interleukin-1β (IL-1β) and tumor necrosis factor α (TNF-α), can also generate ROS. Phthalates—DEHP, DBP, or DEP, have been reported to produce ROS and lipid peroxidation. Epidemiological studies indicated that phthalate metabolites are associated with oxidative stress [199]. BPA has been shown to modulate neuronal activity by both dependent and independent of estrogen receptors, GPER, or estrogen-related receptor-γ pathways [200]. BPA can lower cell viability and induce cell death by mediating calcium influx, generation of ROS, and activation of MAPK and caspase 3 [201]. A similar chemical to BPA, BPAF or Bisphenol AF, was shown to cause hippocampal cell death in mouse neuronal cells (HT-22), by increasing cellular calcium, generation of ROS, and activation of p38, JNK, and caspase 3 [202]. BPA administered to male rats was found to increase malondiadehyde levels, decrease glutathione and superoxide dismutase activity in the cerebrum and cause upregulation of p53 and CD95-Fas and activation of capsases 3 and 8 causing cell death [203], with melatonin attenuating the effects of BPA. All three metalloestrogens—Cd, As and Mn produce oxidative stress in both cell lines and animal models [1,151,160,167]. Different cellular signaling pathways may operate in response to varying levels of estrogen or EED-induced ROS, leading to genotoxic damage, cell apoptosis, or cell growth. At high levels of ROS, cells receiving genotoxic insults, if not repaired, may engage the apoptotic pathways. There is increasing evidence supporting that estrogen or EED-induced alterations in the genome of cells are produced by oxidative attack. Furthermore, ROS generated by estrogen or EED exposure in combination with receptor-mediated proliferation of genetically damaged cells may be involved in pathology of brain diseases. Inhibition of Akt and ERK/CREB/BDNF pathways in the hippocampus by maternal BPA exposure has been suggested to impair object recognition memory in the male offsprings [204]. In cultured hippocampal neurons, BPA was demonstrated the promote changes in dendritic morphology by the phosphorylation of the NMDA receptor subunit NR2B. BPA was shown to induce ERK1/2 activation at low concentrations in cerebellar granule cells and show neurotoxic effects. Recently, BPA-mediated neurodegeneration has been reported to be a result of modulation of autophagy through involvement of AMPK and mTOR pathways [205]. BPA has recently been shown to disrupt insulin signaling, where glucose uptake into the CNS was affected, with increase phosphorylated tau and APP observed in male mice. The adverse effects of BPA on insulin signaling and GLUTs may contribute to the increment of p-tau and APP in the brain of adult offspring. Therefore, perinatal BPA exposure might be a risk factor for the long-term neurodegenerative changes in offspring male mice [206].Phthalates have been demonstrated to induce nuclear transcription factors that are activators of telomerase reverse transcriptase (TERT), which has a role in tumor promotion [207], increase levels of phospho-tau, a precursor to AD [129], and affects dendritic spine density [120]. Recent reviews have also indicated disruption of thyroid homeostasis, disruption of calcium signaling, peroxisome proliferator-activate receptors (PPAR) activation, and adverse lipid metabolism as other potential mechanism [208].

NRF-1 network genes interacting with individual EED impacting Brain Health: NRF1 (synonyms: α palindromic-binding protein, α-PAL), a mammalian homolog to the P3A2 sea urchin and erect wing (ewg) Drosophila protein, is a redox sensitive transcription factor [209,210]. NRF-1 proteins have been shown to interact with a broad spectrum of transcription factors; and the NRF-1 recognition site is one of the seven transcription factor binding sites which are most frequently found in the proximal promoters of ubiquitous genes [8,9], indicating a broader spectrum of target genes for NRF-1. NRF-1 mediate oxidative stress responses by regulating the expression of genes involved in cell cycle, DNA repair, cell apoptosis and mitochondrial biogenesis. NRF1 is highly expressed in human fetal brain [10]. A recent report based on ChIP Seq data from SK-N-SH human neuroblastoma cells showed that NRF1 DNA motif(s) are present in the promoters of 2470 genes [9]. This study suggested that dysregulation of NRF1 and its targets may be involved in the pathogenesis of neurodegenerative diseases. Seven out of 2470 NRF1 target genes have significant relationships with several neurodegenerative conditions. For examples, NRF1 target genes- PARK2, PARK6 (PINK1), PARK7, PAELR (GPR37) are associated with Parkinson’s disease. NRF1 target genes -PSENEN AND MAPT are involved in Alzheimer’s disease. TAF4, a NRF1 target gene, is associated with Huntington’s disease. NRF1 as a potential important player in AD neighbors with five (CREB1, ESR1, NFE2L2, NOS1 and TP53) genes involved in AD [211]. Levels of NRF1 and its target gene- transcription factor A of the mitochondria (TFAM) are significantly decreased in hippocampal tissues in AD autopsy brains [212]. Many estrogen signaling pathway genes are NRF1 target genes, which may be also associated with neurodegenerative diseases [9]. Therefore, we used the Comparative Toxicogenomics Database (CTD) based on curated information about chemical-gene/protein interactions, chemical-disease and gene-disease relationships, to analyze the gene-EDC interactions associated with both estrogen and NRF1 signaling pathways, and neurodegenerative diseases. Findings of EDCs-modified estrogen signaling and NRF1 signaling genes with exposure to natural estrogen, pharmacological estrogen, PCBs, phthalate, BPA, As, Cd, or Mn are summarized in Table 4 and Table 5 and discussed below.

**Figure 3:**
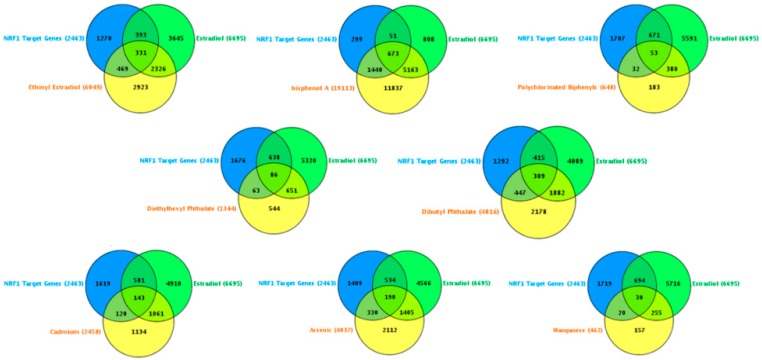
A Venn diagram showing the individual pharmacological estrogen or estrogenic Endocrine Disruptor (EED) modified genes common to both NRF1 and/or estrogen signaling pathways. NRF1 target genes were mapped between 17-β estradiol (E2) and pharmacological estrogen-ethinyl estradiol or PCBs or BPA or phthalates or Cd or As or Mn.

17-β Estradiol interacting genes which are also NRF1 target genes: The CTD search results showed that there were 6695 genes related to 17-β estradiol (E2) (Figure 3). The 724 genes out of the 6695 E2 modified genes are NRF1 target genes (Tables S1 and 2, Figure 3). Three of the common E2 and NRF1 target genes, GPR37, MAPT, and PSENEN are associated with neurodegenerative disease ([9], Table 4). Table 4 showing enriched pathway analysis revealed the top pathways associated with E2 responsive NRF1 target genes, that included: (1) Disease (86 genes); (2) Metabolism (86 genes); (3) Gene Expression (77 genes); (4) Signal Transduction (72 genes); (5) Immune System (67 genes); (6) Cell Cycle (62 genes); (7) Metabolic pathways (62 genes); (8) Metabolism of proteins (48 genes); (9) Developmental Biology (43 genes); and (10) Mitotic M-M/G1 phases (35 genes).

Ethinyl Estradiol Interacting genes common to both E2 and NRF1 target genes: The CTD search revealed that there were 6049 genes related to the active estrogenic chemical in oral contraceptives—ethinyl estradiol (EE) (Figure 3). Out of the 6049 EE modified genes, 800 genes are NRF1 target genes and 331 genes are target genes of both E2 and NRF1 (Tables S1 and S2, Figure 3). Two of the genes, MAPT and PSENEN, observed interacting with all three EE, E2 and NRF1 are associated with neurodegenerative disease [9]. Table 4 showing enriched pathway analysis revealed the top pathways associated with the number of common E2, E2 and NRF1 target genes, that included: (1) Metabolism (49 genes); (2) Disease (48 genes); (3) Gene Expression (43 genes); (4) Immune System (36 genes); (5) Cell Cycle (35 genes); (6) Metabolic pathways (33 genes); (7) Signal Transduction (33 genes); (8) Metabolism of Proteins (28 genes); (9) Developmental Biology (25 genes); and (10) Cancer Pathways (22 genes).

BPA and Interactions with NRF1 Target Genes: The CTD search revealed there are 19,113 genes related to BPA. Out of the 19,113 interacting genes, 2113 are NRF1 target genes, and 673 are target genes of both BPA and E2 (Tables S1 and S2, Figure 3). Three of the genes, *GPR37*, *MAPT* and *PSENEN* observed interacting with all three BPA, E2, and NRF1 are associated with neurodegenerative disease [9]. Enriched pathway analysis revealed the top pathways associated with number of common BPA/E2/NRF1 target genes, that included: (1) Metabolism (85 genes); (2) Disease (84 genes); (3) Gene Expression (71 genes); (4) Signal Transduction (68 genes); (5) Immune System (66 genes); (6) Cell Cycle (61 genes); (7) Metabolic Pathways (58 genes); (8) Metabolism of Proteins (47 genes); (9) Developmental Biology (41 genes), and Cancer Pathways (34 genes) (Table 4).

Phthalates and Interactions with NRF1 Target Genes: The CTD search revealed there were 4816 genes interacting with dibutyl phthalate (DBP) and 1344 genes with diethylhexyl phthalate (DEHP). The 756 DBP interacting genes are NRF1 target genes, whereas 149 DEHP interacting genes are NRF1 target genes. Among DBP responsive genes, there are 309 genes common E2 and NRF1 target genes and 86 DEHP associated genes are common E2 and NRF1 target genes (Tables S1 and S2, Figure 3). MAPT, a common DEHP, E2 and NRF1 target genes are associated with neurodegenerative disease [9]. Enriched pathway analysis revealed the top pathways associated with number of common DBP/E2/NRF1 target genes, that included: (1) Metabolism (56 genes); (2) Disease (45 genes); (3) Gene Expression (39 genes); (4) Metabolic Pathways (38 genes); (5) Cell Cycle (34 genes); (6) Signal Transduction (32 genes); (7) Immune System (31 genes); (8) Metabolism of Proteins (25 genes); (9) Mitotic M-M/G1 phases (23 genes), and Developmental Biology (21 genes). Enriched pathway analysis of E2/NRF1/DEHP-responsive genes revealed the top pathways for gene involvement that includes: (1) Metabolism (21 genes); (2) Metabolic pathways (15 genes); (3) Disease (15 genes); (4) Immune System (12 genes), (5) Metabolism of proteins (11 genes); (6) Cell Cycle (9 genes); (7) Cellular response to stress (8 genes); (8) Cancer Pathways (8 genes); (9) Developmental Biology (8 genes); and (10) Cell cycle (5 genes) (Table 4).

Polychlorinated Biphenyls and Interactions with NRF1 Target Genes: There were 648 genes interacting with polychlorinated biphenyls (PCBs). The 433 PCBs genes are NRF1 target genes, and the 53 PCBs genes are common E2 and NRF1 target genes (Tables S1 and S2, Figure 3). Enriched pathway analysis of common PCBs/E2/NRF1-associated genes revealed the top pathways for gene involvement that included: (1) Cell Cycle (13 genes); (2) Mitotic M-M/G1 phases (9 genes); (3) DNA replication (4 genes); (4) Cell Cycle (4 genes); (5) DNA replication and repair (3 genes); and (6) Pancreatic cancer (3 genes) (Table 4).

Cadmium and Interactions with NRF1 Target Genes: Using CTD search we found 2458 genes interacting with cadmium (Cd). The 263 Cd interacting genes are NRF1 target genes and 143 interacting genes are common E2 and NRF1 target genes (Tables S1 and S2, Figure 3). *GPR37* gene, a common Cd/E2/NRF1 gene, is associated with neurodegenerative disease [9]. Enriched pathway analysis of Cd/E2/NRF1 common genes revealed the top pathways that included: (1) Metabolism (26 genes); (2) Gene Expression (25 genes); (3) Disease (24 genes); (4) Signal Transduction (21 genes); (5) Metabolic pathways (20 genes); (6) Immune System (18 genes); (7) Cell Cycle (17 genes); (8) Cancer Pathways (15 genes); (9) Developmental Biology (15 genes) and (10) Metabolism of proteins (12 genes) (Table 4).

Arsenic and Interactions with NRF1 Target Genes: The CTD search revealed there were 4037 genes related to arsenic (As). The 520 As interacting genes are NRF1 target genes and the 190 as interacting genes are common E2 and NRF1 target genes (Tables S1 and S2, Figure 3). Enriched pathway analysis of As/E2/NRF1 common genes revealed the top pathways that included: (1) Metabolism (33 genes); (2) Immune System (30 genes); (3) Disease (27 genes); (4) Signal Transduction (24 genes); (5) Developmental Biology (22 genes); (6) Metabolic pathways (20 genes); (7) Cell Cycle (19 genes); (8) Gene Expression (19 genes); (9) Cancer Pathways (17 genes); and (10) Cellular responses to stress (16 genes) (Table 4).

Manganese and Interactions with NRF1 Target Genes: Using CTD search we found 462 genes interacting with manganese (Mn). The 50 Mn interacting genes are NRF1 target genes and the 30 Mn interacting genes are common E2 and NRF1 target genes (Tables S1 and S2, Figure 3). Enriched pathway analysis of common Mn/E2/NRF1 genes revealed the top pathways that included: (1) Cellular responses to stress (6 genes); (2) Developmental Biology (5 genes); (3) Tuberculosis (4 genes); (4) p53 signaling pathway (3 genes), Small cell lung cancer (3 genes), Apoptosis (3 genes), Cell cycle (3 genes) (Table 4).

Association of EED interacting genes common to both E2 and NRF1 targets with Neurodegenerative Diseases: Table 5 as well as Tables S1 and S2 summarize EEDs modified genes, which are common E2 and NRF1 target genes and their involvement with the specific type of brain disease, such as AD, PD, HD, ALS, Autism Spectrum Disorder, and Brain Neoplasms. Out of the 6643 E2 interacting genes, there were 1413 genes associated with nervous system disease.

The CTD search of E2 interacting genes in AD revealed 61 genes: *ACE*, *ACHE*, *AMFR*, *APBB2*, *APOE*, *APP*, *ARC*, *ATP5A1*, *BAX*, *BCHE*, *BCL2*, *BDNF*, *BIN1*, *CALM1*, *CASP3*, *CHRNA7*, *CHRNB2*, *CLU*, *CRH*, *CYP46A1*, *DHCR24*, *DPYSL2*, *EIF2S1*, *ENO1*, *EPHA1*, *ESR1*, *F2*, *GAPDHS*, *GSK3B*, *HFE*, *HMOX1*, *IGF1*, *IGF1R*, *IGF2*, *IGF2R*, *IL1B*, *INS*, *INSR*, *LEP*, *MAOB*, *MAPT*, *MIR146A*, *MPO*, *NOS3*, *NPY*, *PAXIP1*, *PICALM*, *PLAU*, *PPARG*, *PRNP*, *PSEN2*, *SLC2A4*, *SOD2*, *SORL1*, *TF*, *TFAM*, *TNF*, *TPI1*, *TREM2*, *VEGFA*, *VSNL*. The bold indicates E2 interacting genes which are NRF1 target genes. With AD, the six E2-responsive genes are NRF1 target genes: *APBB2*, *DPYSL2*, *EIF2S1*, *ENO1*, *MAPT*, and *PAXIP1*. These genes are also responsive to the following EEDCs: ethinyl estradiol (*APBB2*, *DPYSL2*, *EIF2S1*, *ENO1*, *MAPT*, and *PAXIP1*), BPA (*APBB2*, *EIF2S1*, *ENO1*, *MAPT*, and *PAXIP1*), dibutyl phthalate (*DPYSL2*, *EIF2S1*, *ENO1*), diethylhexyl phthalate (*DPYSL2* and *MAPT*), cadmium (*ENO1*), arsenic (*ENO1* and *MAPT*), and manganese (*MAPT*) (Table 5).

With PD, the 8 E2-responsive NRF1 target genes are: *GAK*, *HSPA9*, *MAPT*, *PARK2*, *PARK7*, *PINK1*, *RPL14*, *AND VPS35*. These genes are also responsive to the following EEDCs: ethinyl estradiol (*HSPA9, MAPT*), BPA (*HSPA9*, *MAPT*, *RPL14*), dibutyl phthalate (*HSPA9*, *RPL14*), diethylhexyl phthalate (*HSPA9*, *MAPT*), cadmium (*RPL14*), and arsenic *(HSPA9*, *MAPT*) (Table 5). The CTD search revealed that PCBs-interacting 9 genes: *HMOX1*, *IL6*, *NQO1*, *RPS8*, *SLC18A2*, *SNCA*, *SOD1*, *SOD2*, *TNF* are associated with Parkinson Disease. Both SOD and TNF E2 interacting genes are NRF1 target genes.

With HD, the 2 E2-responsive NRF1 target genes are: *AIFM1* and *IP6K2*. These genes are also responsive to the following EEDCs: ethinyl estradiol (*AIFM1*), BPA (*AIFM1* and *IP6K2*), dibutyl phthalate (*IP6K2*), cadmium (*AIFM1*), and arsenic (*IP6K2*) (Table 5).

With ALS, the E2-responsive NRF1 target genes are *CHMP2B* and *GSR*. These genes are also responsive to the following EEDCs: ethinyl estradiol (*GSR*), BPA (*GSR* and *CHMP2B*), dibutyl phthalate (*GSR*), diethylhexyl phthalate (*GSR*), cadmium (*GSR*), and arsenic (*GSR*) (Table 5).

With ASD, the E2-responsive NRF1 target genes are *CIRBP*, *PCDH9*, and *GTF2I*. These genes are also responsive to the following EEDCs: ethinyl estradiol (*CIRBP* and *GTF2I*), BPA (*CIRBP*, *GTF2I*, and *PCDH9*), polychlorinated biphenyls (*CIRBP*), cadmium (*CIRBP*), and arsenic (*PCDH9*) (Table 5).

With brain neoplasms, the E2-responsive NRF1 target genes are *PCNA*, *PTCH1*, and *RELA*. These genes are also responsive to the following EEDCs: ethinyl estradiol (*PCNA* and *PTCH1*), BPA (*PCNA* and *RELA*), dibutyl phthalate (*PCNA* and *PTCH1*), cadmium (*RELA*), arsenic (*PCNA* and *PTCH1*), and manganese (*RELA*) (Table 5).

### 3.1. NRF1, Mitochondrial Dysfunction, and Neurodegenerative Diseases

Mitochondria are known as the powerhouses of cells. They are important for cell viability and function [213]. They control cell process such as energy production, calcium signaling, and apoptosis [213]. Research has suggested that mitochondrial dysfunction is a cause and not a result of neurodegenerative diseases [214]. It is suggested mitochondrial dysfunction plays a major role, in AD, PD, HD, and ALS through the oxidative phosphorylation dysfunction [202]. Mitochondrial dysfunction may also increase ROS generation, cause abnormal protein-protein interactions, and may lead to loss of cellular integrity and cell death [215]. There is also evidence to suggest mitochondrial dysfunction may play a role in neuronal plasticity and maintenance [215]. Sex steroids have been observed to regulate mitochondrial function [216], and provide targets for EEDs such as BPA, phthalates, polychlorinated biphenyls, metals, oral contraceptives, and hormonal replacement therapies [5]. NRF1 regulates the expression of nuclear genes that encode that encode mitochondrial proteins that function in metabolic pathways such as the trichloroacetic acid cycle (TCA), oxidative phosphorylation, heme synthesis, and in mitochondrial DNA replication and transcription (e.g., mitochondrial transcription factor A [Tfam] [9,217]. For example, NRF1 and NRF2 are regulators of all 13 COX subunit genes in neurons which are integral to mitochondrial function [218]. By generating energy (ATP), and regulating subcellular Ca^2+^ and redox homoeostasis through modulating mitochondria biogenesis genes as well as neuroplasticity regulating genes, NRF1 may play important roles in controlling fundamental processes in neuroplasticity, including neural differentiation, neurite outgrowth, neurotransmitter release and dendritic remodeling. Since mitochondrial dysfunction has been implicated as a possible pathway for neurodegenerative diseases [214], gene targets of NRF-1 directly affect brain health and may provide insight into mechanisms of these diseases. PGC1α acts as a master inducer of mitochondrial biogenesis and respiration through regulating NRF1. PGC1α has also been implicated in Alzheimer’s disease (AD), amyotrophic lateral sclerosis (ALS) and Duchenne muscular dystrophy. In ALS patients and mice, significant alterations in mRNA expression of PGC-1α, NRF1, NRF2, and Tfam were observed [219]. The PGC-1 transcription impairment in the striatum and a significant decrease of PGC-1 and PGC-1A but unchanged NRF-1 and Tfam mRNA expression in muscle tissue have been reported in HD patients [220,221]. In a mouse model of HD, decreased mRNA expression of PGC-1 and Tfam in muscle tissue correlated with reduced numbers of mitochondria, whereas PGC-1A, NRF-1, and further downstream target genes were not significantly reduced [220]. These observations have led to the suggestion that the progressive muscle atrophy and morphologic abnormalities of neuromuscular junctions that occur in HD mice could be a consequence of impaired PGC-1 and target gene expression [220]. PGC-1, NRF1, and MnSOD are mainly localized in neurons suggests that these protective effects are of neuronal rather than glial origin. Expression of PGC1α is reduced in AD patients and in the transgenic 2576 mouse model of AD [222,223,224,225]. Importantly, PGC1α in cell models of AD ameliorates their phenotype [212,224]. Since levels of NRF1 are significantly decreased in both Alzheimer’s disease hippocampal tissues and AD-causing amyloid precursor protein mutant cells, impaired mitochondrial biogenesis is implicated to contribute to mitochondrial dysfunction in Alzheimer’s disease. NRF-1 has the ability to distinctly co-ordinate mitochondrial functions by tailoring nucleo-mitochondrial interactions to a specific cellular context, such as differentiation, stress stimulus and cell lineage. Furthermore, expression of PGC1-α, NRF1, NRF2, and transcription factor A were found to be significantly reduced in both AD hippocampal tissue as well as APPswe M17 cells which overexpress APP protein, which supports impaired mitochondrial biogenesis likely contributes to mitochondrial dysfunction [212]. When mtDNA contained within mitochondria undergoing phagocytosis is considered into account, it appears that total hippocampal neuron mtDNA may actually increase [226]. Increased mitochondrial mass or mitochondrial mass markers have been observed in the AD hippocampus of the healthiest remaining neurons that paradoxically appear to be increased [227]. Considering these disparate findings suggesting that neuron mitochondrial mass or mitochondrial mass markers increase with advancing age, Swerdlow suggested a physiologic difference between brain aging and the AD brain, which is the ability of healthy hippocampal neurons to adapt to mitochondrial stress by mounting a compensatory mitochondrial biogenesis response [228,229,230].

### 3.2. NRF1-Mediated Regulation of Neurogenesis and Synaptogenesis

Estrogen has been demonstrated to have a significant role in controlling neural progenitors in the developing embryonic brain. Estrogen has been shown to induce proliferation and differentiation of neural progenitor [231,232,233]. Neural progenitors have also been demonstrated to express estrogen receptors [232,233], which provide a site in EEDCs to exert their effects. EEDCs have been shown to cross the placental and blood brain barrier [6], but the mechanisms regarding the effects on the early stages of neural development are largely not known. Studies suggest neural development is affected by exposures to EEDCs. Low-dose exposure to BPA and BPS has been shown to induce hypothalamic neurogenesis in an embryonic zebrafish at a point in time analogous to the second trimester of human development [77]. Several classes of phthalates were shown to prevent neural stem cell proliferation in a rat mesencephalic stem cell model [234]. Polychlorinated biphenyls have also been demonstrated to interfere with neuronal cell differentiation in a rat embryonic neural stem cell model [235]. Arsenic was shown to substantially inhibit neuronal differentiation in human embryonic neural stem cells [236]. Manganese has also been demonstrated to cause cell death in a rat neural stem cell model through a mitochondrial-mediated pathway [237]. Both neurogenesis and synaptogenesis are controlled by NRF1 regulated genes. We now know that multipotent adult neural stem cells (NSCs) have differentiation potential to give rise to all three main neuronal cell types, the neurons, astrocytes and oligodendrocytes. Multipotent adult neuronal stem cells (NSCs) through neurogenesis (birth of brain cells or the process by which brain cells are generated from neural stem cells and progenitor cells) replenish loss of cells in discrete regions of the human brain. A decreased rate of neurogenesis as a result of impairment in NSCs with increasing age in the adult brains of human is considered to contribute to the neurodegenerative process and subsequently resulting in memory deficits and learning impediments during the pathogenesis of Alzheimer’s disease. Cholinergic neurons of the forebrain are most affected by the neurodegenerative process during the pathogenesis of Alzheimer’s disease. Neuronal loss is one of the neuropathological hallmarks of AD. The control of stem cell homeostasis within tissues such as the balance between proliferation, differentiation, apoptosis and senescence is strictly linked to the regulation of tissue repair and regeneration. It has been recently reported that estrogen helps in growth, survival and differentiation of either embryonic stem cells or tissue-specific stem/progenitor cells. New adult stem cells (ASCs) replenishing losses due to neurodegenerative processes underlying AD. EEDs may interfere with new neural stem cells generation. The generation of new neurons from neural progenitor stem cells, the growth of axons and dendrites and the formation and reorganization of synapses are examples of neuroplasticity. All these processes seem to be regulated by nuclear respiratory factor 1 (NRF1) target network genes. For example, recently, it has been shown that NRF1 regulates neurite outgrowth—a critical process in neuronal development in neuroblastoma cells and hippocampal neurons by regulating its target gene, Synapsin 1 [238]. Another fifteen genes involved in different biological processes of neurons, cell cycle-related genes—*MAPRE3*, *NPDC1*, *SUV39H2*, *SKA3*, transport-related genes—*RAB3IP*, *TRAPPC3*, signal transduction-related genes—*SMAD5*, *PIP5K1A*, *USP10*, *SPRY4*, transcription-related genes—*GTF2F2*, *NR1D1*, and regulation of GTPase activity-related genes—*RHOA*, *RAPGEF6*, *SMAP1*, have been reported to contain NRF1 binding motif(s) in their promoters and mRNA levels of 12 of these genes are regulated by NRF-1 [239]. Overexpression or knockdown of MAPRE3, NPDC1, SMAD5, USP10, SPRY4, GTF2F2, SKA3, RAPGEF6 positively regulates, where as RHOA and SMAP1 negatively regulates neurite outgrowth. Three hypothetical genes—*FAM134C*, *C3orf10*, and *ENOX1* involved in neurite outgrowth are regulated by NRF1. FAM134C positively regulates and C3orf10 negatively regulates neurite outgrowth [240]. In summary, it appears that NRF-1 regulates neurite outgrowth through cell cycle-, transport-, signal transduction-, transcription-, regulation of GTPase activity-related genes and hypothetical genes. This suggests that NRF1 regulates neuronal differentiation through a variety of biological processes.

NMDA receptors are very important for regulating neuronal plasticity including learning and memory. Soluble β-amyloid (Aβ) oligomers (Aβ), considered to be involved in the pathogenesis of Alzheimer’s disease (AD), have been reported to influence synaptic dysfunction through interacting with glutamatergic receptors of the NMDA type responsible for maintaining glutamate homeostasis such as uptake and release [241]. There is a clinical relevance of above interactions based on the NMDA receptor antagonist- memantine. This NMDA receptor antagonist is used clinically in the treatment of AD. NRF1 has recently been shown to functionally regulate important subunits of NMDA receptors—NR1 and NR2b (Grin1 and Grin2b) and AMPA receptor subunit 2 (GluR2) [242] in response to changing neuronal activity. NRF1 and NRF2 have been shown to concurrently regulate both GRIN1 and GRIN2B [243]. The *GRIN2B* gene which encodes NMDA receptor subunit NR2B has been suspected to play a role in the development of AD [244] which makes its relationship with NRF1 a possible route of neurodegeneration. Evidence suggests that the DNA methylation competes with transcriptions factors [245], which may indicate a possible mechanism for neurodegenerative conditions to take place, if NRF1 binding is affected. NRF1 has also been demonstrated to interact with macroH2A histone which stabilizes and positions nucleosomes and reduces transcriptional variability [246]. NRF1 has also been shown to work with chromosomal conformations to facilitate the expression of GRIN2B, which when affected, results in impairments in working memory [247]. NRF1 also has a role in the transport of NMDA receptors to dendrites by regulating the Kif17 gene [248]. This suggests that NRF1 is actively involved in the regulation of glutamatergic synaptic transmission in neurons and also possibly in brain diseases.

In vitro and in vivo BPA exposure via disrupting a chloride ion transporter Kcc2 gene expression in neurons through epigenetic mechanisms involving NRF1 target gene- Methyl-CpG binding protein 2 (MeCp2) is considered to be involved neurodevelopmental toxicity. This could possibly help to explain a NRF1 role in BPA-associated pathogenesis of human neurodevelopmental disorders [249].

In a study examining the effects of manganese exposure with HT disease pathology, manganese exposure adversely affects medium spiny neuron dendritic length and spine density which is associated with a decrease brain-derived neurotrophic factor which caused alterations in the Htt gene which causes HT [250]. In study using a monkey model, exposure to manganese was shown to promote α-synuclein positive cells, which indicate the development of PD and dementia [251]. Another study using a monkey model found the amyloid-β precursor-like protein 1 (APLP1), and indicator in the development of AD, was upregulated when exposed to manganese [252]

The accumulation of the β-amyloid protein outside neurons and an abnormal form of the protein tau inside neurons are considered to contribute to the development of Alzheimer’s. During the progression of Alzheimer’s disease, chemical transfer at synapses begins to fail, the number of synapses declines, and neurons eventually die. All these processes seem to be regulated by nuclear respiratory factor 1 (NRF1) target network genes. In addition to the regulation of neuroplasticity controlling genes, PSENEN (PEN2), a component of the γ-secretase complex, APLP1 (an isoform of APP) and MAPT (tau) are three principal NRF1 target genes directly associated with the development Alzheimer’s [9]. All three proteins—APP, presenilin-1 and tau are considered to play important roles in developmental and synaptic plasticity, and alterations in these proteins have been shown to modulate neurogenesis and synaptic plasticity [253]. The suppression of NRF-1 activity can induce neurodegeneration through growth arrest and increases in apoptosis and reduces growth and survival of neural stem cells. Therefore, NRF1 is an emerging potential target for therapeutic intervention for brain diseases, including AD.

### 3.3. Understanding Sex Bias and NRF1 Regulated Genes-EEDs Interactions in AD

Sex differences in nervous system diseases, such as multiple sclerosis, Parkinson’s disease, and Alzheimer’s disease, exist in prevalence, severity, and progression of pathologies and consequently has become a major obstacle for the treatment. Cognitive disturbances are frequent in brain health deficit-related disease. Men show greater cognitive impairment in schizophrenia, whereas women show more severe dementia and cognitive decline with Alzheimer’s disease. Low dose of raloxifene exposure to BPA alters the expression of hypothalamic ERα and ERβ (Esr1 and Esr2) expression in a sex-specific manner in the developing brain [254].

Menopausal hormone therapy (HT; 0.625 mg/day conjugated equine estrogens (CEE) therapy alone (CEE-Alone) and in combination with 2.5 mg/day medroxyprogesterone acetate (CEE + MPA)) has recently been shown to produce significant Gray Matter (GM) losses compared to the placebo groups in the anterior cingulate and the adjacent medial frontal gyrus, and the orbitofrontal cortex and risk for cognitive impairment and dementia in older women [255].

Alzheimer’s disease (AD) disproportionally affects women (F:M = 2:1) and the total number of Americans aged 65 y with AD is projected to increase from 5.1 million to 13.8 million by 2050. Despite tremendous progress in understanding the pathogenesis of AD, the molecular basis underlying the sex-dependent differences in AD remains largely unknown and consequently has become a major obstacle for the development of new sex-based molecular targets of AD. Hormone replacement therapy (HRT) and anti-estrogen therapy (raloxifene) are focused on circulating estrogens, as measured by blood levels, but not based on brain estrogen levels. This is one of the main reasons why such endocrine therapy is unable to produce a significant beneficial effect in human AD [256]. There is only one study in which human brain levels of estrogens have been measured; and these hormones do not change significantly with age in postmenopausal women, rather estradiol (E2) showed an increasing trend from age 60–100 years [257]. There is no change in brain levels of E2 in the male 60–79 years age range. In contrast, preclinical data are ample and unequivocal that brain estrogen deficiency induces Alzheimer’s pathology, and early E2 treatment, not late treatment, prevents AD pathologies in APP transgenic mice [258]. It is not clear why a sex-specific bias occurs in AD, even though brain hormone levels are similar between the two sexes. NRF1 targets have been shown to have roles in the development of AD. The NMDA receptor subunit GRIN1 and GRIN2B, have been shown to be regulated by NRF-1 [243]. GRIN1 and PSD-95 protein were found to be significantly increased in AD cases compared to controls and were found in areas of the brain that were reactive for anti-tau antibody AD2 [259]. A variant of GRIN2B was found to be overexpressed in AD patients and individuals with mild cognitive impairment [260] and may induce lower GRIN2B transcriptional activity as well as NR2B protein expression, which are associated with AD [261]. Other studies support this connection where AB peptides were shown to decrease the number and activity of NMDA receptors [262]. Another study found neurons from AD cases had significantly less mtDNA gene copy numbers from PCR methods compared to controls as well as lessened gene expression of mitobiogenesis factors such as NRF1, NRF2, EER α, and TFAM and the master regulator PGC1 α [263]. Another NRF1 target, prsenilin-2 (PSEN2), an AD-related gene was shown to have increased activity when an overexpression of EGR-1 is takes place [264]. This provides the evidence that NRF1 may also have a role in the regulation of PSEN2. Evidence also suggests the regulation of the transcription factor EGR-1 may be regulated by APP, which may play a role in AD development and memory formation [265]. Mutations in NRF1 targets PSEN1 and PSEN2 may also lead to the development of neurodegenerative disease [266,267,268]. In addition PSEN1 and PSEN2 transcription, expression, promoter activity, and mRNA levels are influenced by the PARKIN protein [269]. PARKIN inactivation as well as PGC-1 α and NRF1 repression is a possible mechanism for neurodegeneration [270].

**Figure 4:**
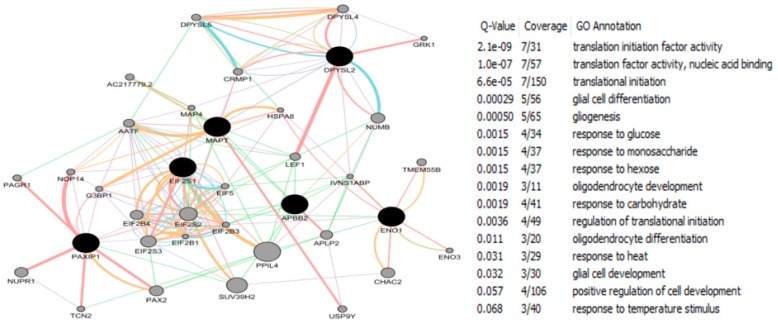
Gene set pathway enrichment analysis of 17-β estradiol (E2) responsive NRF1 target genes associated with Alzheimer’s Disease (AD). The network includes the top 30 E2 related genes in AD in gray. E2 responsive NRF1 genes are in black. Right panel show GO annotations of NRF1 enrichment network genes of E2 associated with biological process pathways involving multiple E2 and NRF1 genes and *Q*-value as a probability showing number of genes out of the total genes annotated to each GO term.

**Figure 5:**
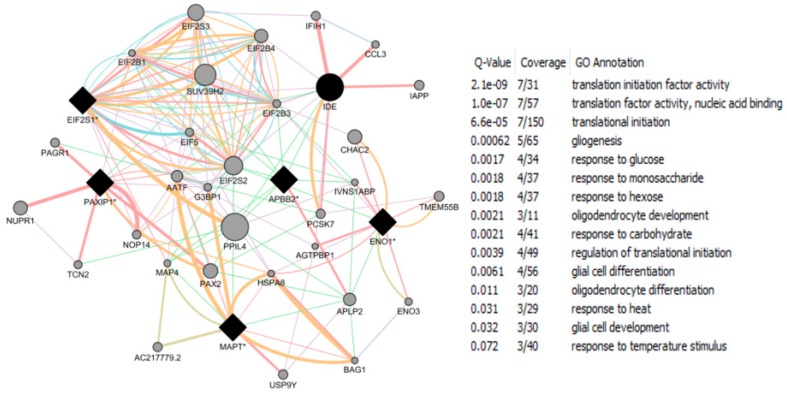
Gene set pathway enrichment analysis of ethinyl estradiol responsive E2 and NRF1 common target genes associated with Alzheimer’s Disease (AD). The network includes the top 30 ethinyl estradiol-associated genes in AD in gray. Ethinyl estradiol responsive NRF1 genes are in black and * genes denoted by a diamond-shaped node are E2 and NRF1. Right panel show GO annotations of E2 and NRF1 enrichment network genes of ethinyl estradiol associated with biological process pathways involving multiple E2 and NRF1 genes and *Q*-value as a probability showing number of genes out of the total genes annotated to each GO term.

**Figure 6:**
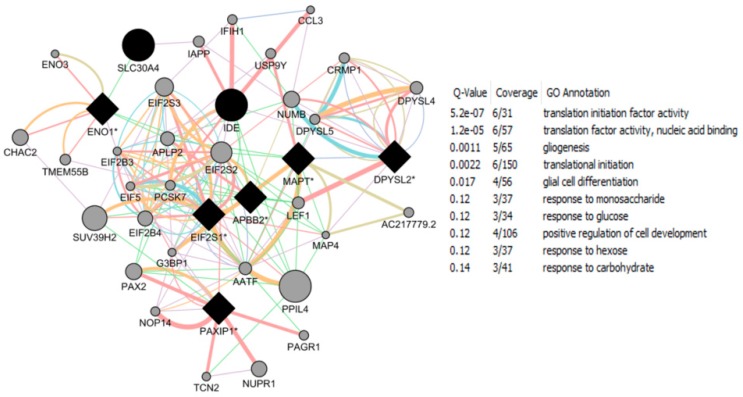
Gene set pathway enrichment analysis of bisphenol-A (BPA) responsive R2 and NRF1common target genes associated with Alzheimer’s Disease (AD). The network includes the top 30 BPA-related genes in AD in gray. Bisphenol-A responsive NRF1 genes are in black. Genes denoted by a diamond-shaped node and * are E2 and NRF1 responsive. Right panel show GO annotations of E2 and NRF1 enrichment network genes of BPA associated with biological process pathways involving multiple E2 and NRF1 genes and *Q*-value as a probability showing number of genes out of the total genes annotated to each GO term.

**Figure 7:**
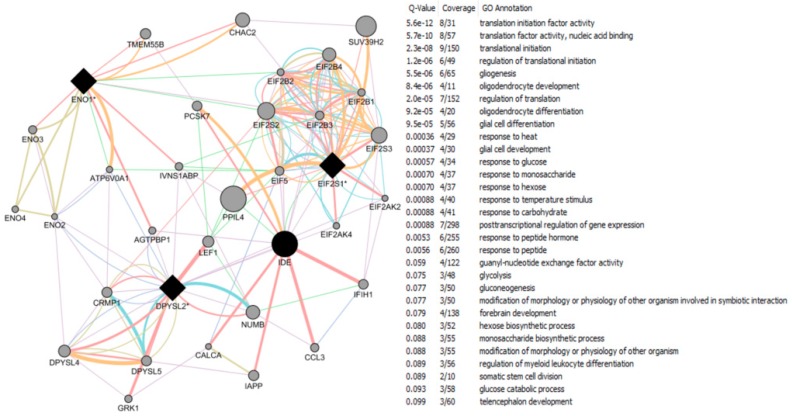
Gene set pathway enrichment analysis of dibutyl phthalate modified E2 and NRF1 common target genes associated with Alzheimer’s Disease (AD). The network includes the top 30 dibutyl phthalate related genes in AD in gray. Dibutyl phthalate responsive NRF1 genes are in black. Genes denoted by a diamond-shaped node and * are E2 and NRF1 responsive. Right panel show GO annotations of E2 and NRF1 enrichment network genes of dibutyl phthalate associated with biological process pathways involving multiple E2 and NRF1 genes and *Q*-value as a probability showing number of genes out of the total genes annotated to each GO term.

**Figure 8:**
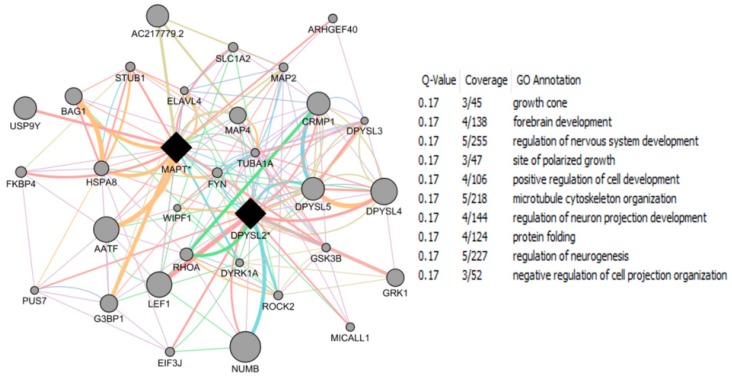
Gene set pathway enrichment analysis of diethylhexyl phthalate responsive E2 and NRF1 common target genes associated with Alzheimer’s Disease (AD). The network includes the top 30 diethylhexyl phthalate related genes in AD in gray. Diethylhexyl phthalate responsive NRF1 genes are in black. Genes denoted by a diamond-shaped node and * are E2 and NRF1 responsive. Right panel show GO annotations of E2 and NRF1 enrichment network genes of diethylhexyl phthalate associated with biological process pathways involving multiple E2 and NRF1 genes and *Q*-value as a probability showing number of genes out of the total genes annotated to each GO term.

**Figure 9:**
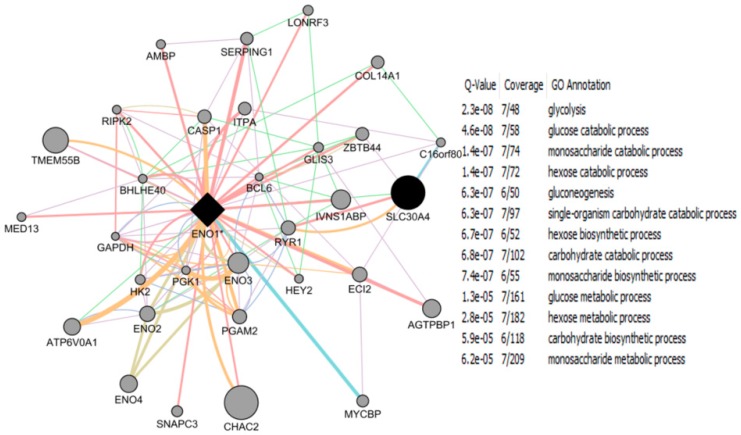
Gene set pathway enrichment analysis of cadmium responsive E2 and NRF1 common target genes associated with Alzheimer’s Disease (AD). The network includes the top 30 Cd related genes in AD in gray. Cadmium responsive NRF1 genes are in black. Genes denoted by a diamond-shaped node and * are E2 and NRF1 responsive. The network includes the top 30 related genes in gray. Right panel show GO annotations of E2 and NRF1 enrichment network genes of cadmium associated with biological process pathways involving multiple E2 and NRF1 genes and *Q*-value as a probability showing number of genes out of the total genes annotated to each GO term.

**Figure 10:**
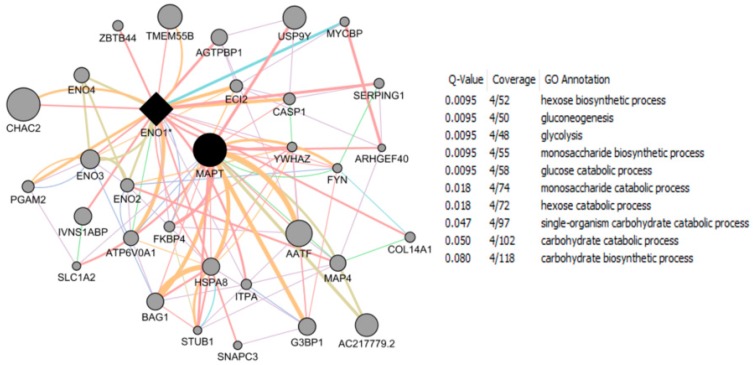
Gene set pathway enrichment analysis of arsenic responsive E2 and NRF1 common target genes associated with Alzheimer’s Disease (AD). The network includes the top 30 arsenic related genes in AD in gray. Arsenic responsive NRF1 genes are in black. Genes denoted by a diamond-shaped node and * are E2 and NRF1 responsive. Right panel show GO annotations of E2 and NRF1 enrichment network genes of arsenic associated with biological process pathways involving multiple E2 and NRF1 genes and *Q*-value as a probability showing number of genes out of the total genes annotated to each GO term.

**Figure 11:**
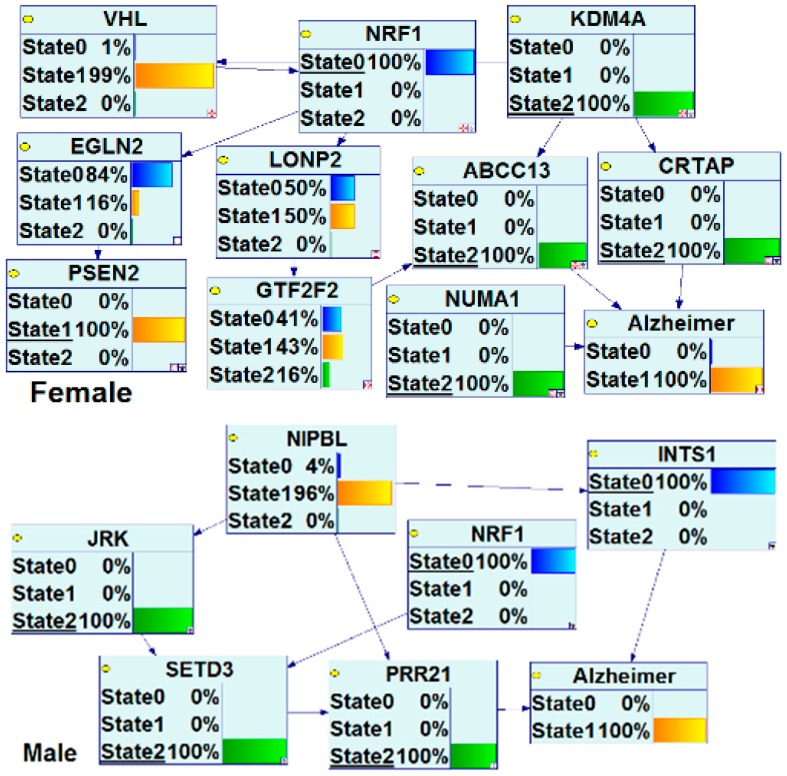
Gender differences in NRF1 network in AD of brain microarray data of 79 subjects of male (15 AD patients, 17 control) and female (17 AD patients, 30 controls) from GEO database [272].

Many genes modified by EEDs are common targets of both (E2) and NRF1 and some of these genes are involved with the specified brain diseases. Therefore, using NRF1-EDC interacting genes identified from CTD (Table 4), we focused our efforts on AD and conducted enrichment pathway analysis, which revealed that many of the NRF1 target genes interacting with each specific EEDC and they are part of pathway of AD (Figure 4, Figure 5, Figure 6, Figure 7, Figure 8, Figure 9 and Figure 10). Enrichment pathway analysis was performed using Cytoscape and Genemania. The search of enriched pathways showed that top 10 E2 interacting genes in AD: *APOE*, *APP*, *ATP5A1*, *CALM1*, *CASP3*, *GSK3B*, *IL1B*, *MAPT*, *PSEN2* and *TNF* underlie the enrichment of the Kyoto Encyclopedia of Genes and Genomes (KEGG) Alzheimer’s disease pathway. With AD, the six E2-responsive genes are NRF1 target genes: *APBB2*, *DPYSL2*, *EIF2S1*, *ENO1*, *MAPT*, and *PAXIP1*. These genes are also responsive to the following EEDs: ethinyl estradiol (*APBB2*, *DPYSL2*, *EIF2S1*, *ENO1*, *MAPT*, and *PAXIP1*) *BPA* (*APBB2*, *EIF2S1*, *ENO1*, *MAPT*, and *PAXIP1*), dibutyl phthalate (*DPYSL2*, *EIF2S1*, *ENO1*), diethylhexyl phthalate (*DPYSL2* and *MAPT*), dibutyl phthalate (*DPYSL2*, *EIF2S1*, *ENO1*) (Figure 5, Figure 6, Figure 7, Figure 8, Figure 9 and Figure 10, Table 5). GO annotations of E2 and NRF1 enrichment network genes of EEDs revealed multiple common E2 and NRF1 genes associated with carbohydrate metabolic pathways, which was common among all EEDs (Figure 4, Figure 5, Figure 6, Figure 7, Figure 8, Figure 9 and Figure 10). Other two biological process pathways showing association with multiple common E2 and NRF1 genes interacting with E2, EE, BPA or phthalates were translation (translation initiation, translation initiation factory activity and translation factory activity, nucleic acid binding) and glial/oligodendrocytes growth and differentiation (Figure 4, Figure 5, Figure 6, Figure 7 and Figure 8). To validate findings from CTD curated data, we conducted Bayesian network (BN) analysis [271] of microarray data of 79 subjects of male (15 AD patients, 17 control) and female (17 AD patients, 30 controls) from Gene Expression Omnibus (GEO) database [272] showed that the female NRF1 gene network is completely different from male human AD patients (Figure 11). For BN analysis, we have first identified 827 genes that showed significant correlations with NRF1 gene expression pattern among 79 subjects.

**Figure 12:**
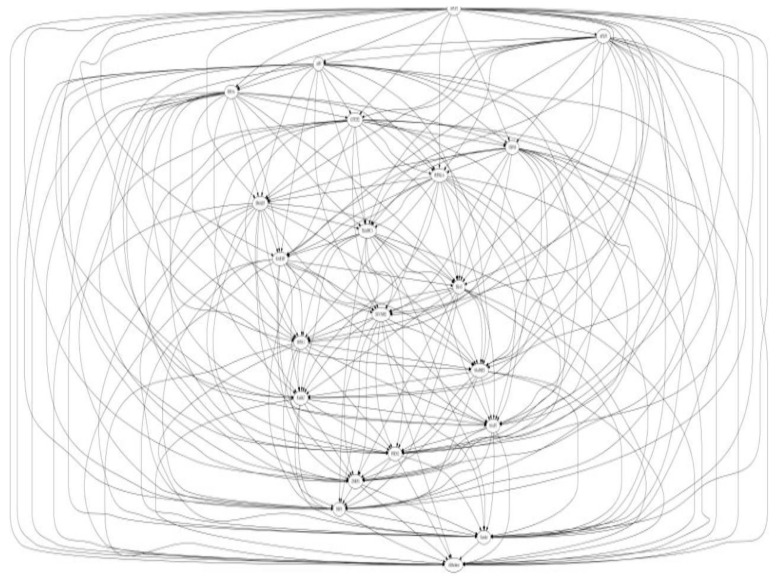
Bene network showing gender and NRF1 association with AD in hippocampal microarray [272].

**Figure 13:**
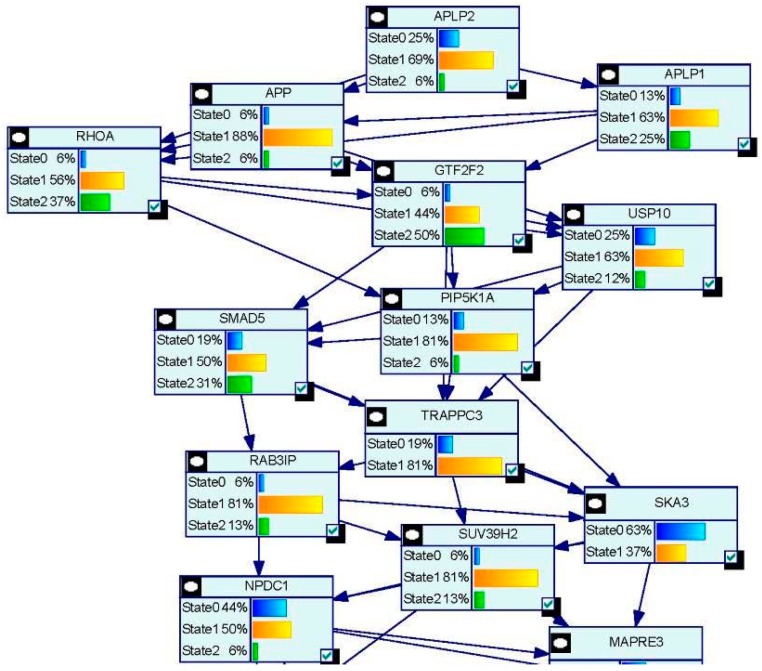
Causal network analysis of gender and NRF1 association with AD in hippocampal microarray [272].

We used Banjo [273] to identify the most likely BN given the male and female data. Figure 11 shows the summarized BN where for each gene, State 0, 1, and 2 represent low expression, no change, and high expression respectively; and for Alzheimer, State 0 and 1 represent a control subject and a subject with AD respectively. When we analyzed only hippocampal microarray, we observed that both gender and NRF1 were associated with AD (Figure 12 and Figure 13). The invertebrate NRF1 homologue, EWG, controls transcription of neuronal genes in Drosophila embryos [210]. A strong association of NRF1with human fetal brain and neural development has been recently reported [208]. NRF1 regulates neurite outgrowth. NRF1 regulates important subunits of NMDA receptors—NR1 and NR2b (Grin1 and Grin2b) and AMPA receptor subunit 2 (GluR2). These findings suggest that NRF1 may be involved in the regulation of brain and neural development [10,274].The neurotoxic amyloid β-peptide (Aβ), the major constituent of senile plaques in AD brains is generated by proteolytic cleavages of the amyloid precursor protein (APP), and the amyloid precursor-like proteins 1 and 2 (APLP1 and APLP2) by β- and γ-Secretases. PSEN2, and presenilin enhancer 2 (PEN2) subunits of γ-Secretase complex, involved in the formation of Aβ, MAPT (tau) and insulin-degrading enzyme (IDE) which degrades Aβ are NRF1 target genes [9]. Accumulation of Aβ is dependent on Aβ formation by MAPT, APP, PSEN2, and PEN2; and loss of IDE (Aβ degrading enzyme). AD-associated NRF1 target genes -APLP1, APP, GRIN1, GRIN2B, MAPT, PSEN2, PEN2, and IDE are also regulated by E2. These NRF-1 regulatable genes alone, or in concert with others, may contribute to the sex-dependent differences in the brain NRF1 network and the molecular mechanisms of prevention of AD by E2.

## 4. Conclusions

**Figure 14:**
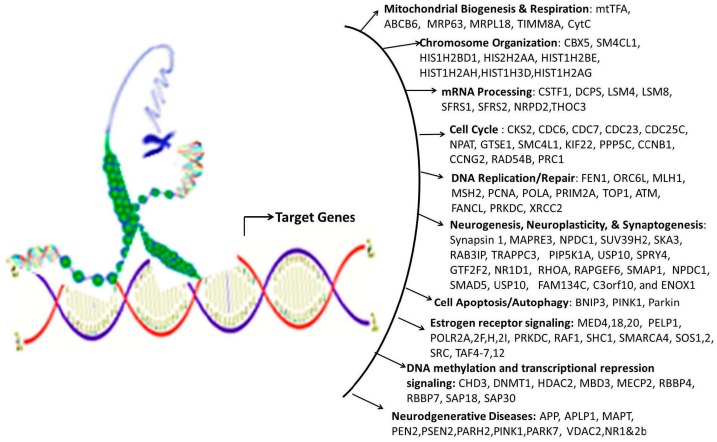
NRF1 regulating targets genes with diverse functions. NRF1 is depicted to recognize the palindromic sequence defined as YGCGCAYGCGCR, where Y and R indicate a pyrimidine or purine nucleotide, respectively, to recruit factors for the transcription of target genes, which are involved in many cellular processes.

A large number of genes (approximately 300) are considered to be altered during the development of complex chronic brain diseases. For example, a minimum of six to eight gene mutations and gene activation by genetic/epigenetic mechanisms is needed for the development of glioblastoma [275]. Similarly, in AD, there are a minimum of 6–8 genes that are involved in the causation of this disease. During the development of an individual from a single cell to prenatal stages to adolescent to adulthood and through the complete life span, humans are exposed to countless environmental and stochastic factors. Environment constitutes everything that surrounds us both internally and externally, including toxicants, hormones, diet, psychosocial behaviors, and life-styles. Like genes, these environmental factors also interact among themselves. It takes 5–15 years to develop the most sporadic brain cancers and other complex chronic brain diseases. A single exposure to an internal or external environmental factor alone cannot explain the development of a complex chronic brain disease, rather it appears that exposure to multiple environmental and stochastic factors across the lifespan and their interactions influence the development of a chronic disease in an individual. The old paradigm is that an orderly progression of changes in disease stem cells through a recognizable series of intermediate states leads toward a predictable endpoint, the disease, in equilibrium with the prevailing environment. In contrast, a more recent view of complex malignant or nonmalignant complex chronic lesion development is based on adaptations of independent disease stem cells. Transitions between a series of different states of disease stem cells are disorderly and unpredictable resulting from probabilistic processes such as death and survival of disease stem cells which make up a malignant or nonmalignant complex chronic lesion. This reflects inherent variability observed in behaviors of different types of cells present in the malignant or nonmalignant complex chronic lesion and the uncertainty of environmental and stochastic factors. In particular, it allows for succession of alternative pathways and endpoints dependent on the chance outcome of interactions among gene–gene, environment-environment, gene–environment and between cells and their environment. These concepts may help in explaining tremendous inter-individual variability in the response to environmental and stochastic stressors, which has hindered our understanding of why a certain individual develops a brain disease when exposed to environmental factors and others remain healthy. EEDs effects on brain go beyond known mechanisms to disrupt the circulatory and neural estrogen function and estrogen-mediated signaling. Bioinformatics analysis of gene-EEDs interactions and brain disease associations identified numerous NRF1 regulated genes that were altered by exposure to estrogen, phthalate or BPA or other EEDCs (*APP*, *APLP1*, *MAPT*, *PEN2*, *PSEN2*, *PARH2*, *PINK1*, *PARK7*, *VDAC2*, *NR1* and *2b*). EEDs -modified genes in brain health deficits are part of estrogen and NRF1 signaling pathways. NRF1 regulates targets genes with diverse functions, including involved in cell growth (*CKS2*, *CDC6*, *CDC7*, *CDC23*, *CDC25C*, *NPAT*, *GTSE1*, *SMC4L1*, *KIF22*, *PPP5C*, *CCNB1*, *CCNG2*, *RAD54B*, *PRC1*), *apoptosis/autophagy* (*BNIP3*, *PINK1*, *Parkin*), mitochondrial biogenesis/respiration (mtTFA, ABCB6, MRP63, MRPL18, TIMM8A, CytC), and neurogenesis/neuroplasticity/synaptogenesis (*Synapsin 1*, *MAPRE3*, *NPDC1*, *SUV39H2*, *SKA3*, *RAB3IP*, *TRAPPC3*, *PIP5K1A*, *USP10*, *SPRY4*, *GTF2F2*, *NR1D1*, *RHOA*, *RAPGEF6*, *SMAP1*, *NPDC1*, *SMAD5*, *USP10*, *FAM134C*, *C3orf10*, and *ENOX1*) (Figure 14). Both phosphorylation and acetylation of NRF-1 by two independent signaling molecules control NRF-1 activation. Activation of a redox sensitive signaling pathway regulates NRF-1 phosphorylation and acetylation events. These two events enhance its activating function by increasing its localization close to nuclear targets and its affinity for promoters of target genes.

This, in turn, may contribute to the susceptibility of normal brain cells to develop disease phenotype. NRF-1 is phosphorylated by cyclin D1-dependent kinase, p38MAPK, ERK and AKT (reviewed in [8]). All of these are redox sensitive kinases. Consistent with the redox-sensitivity of AKT, 4-OH-E2- or H2O2-induced NRF-1 phosphorylation was inhibited by the glutathione peroxidase mimic, ebselen. AKT activity depends on its phosphorylation, which is positively regulated by PI3K and negatively regulated by a class of protein phosphatases [52]. AKT controls translocation of NRF-1 to the nucleus, because translocation of NRF-1 to the nucleus does not occur in PTEN deficient cells. The ability of E2 and H_2_O_2_ to activate AKT may be attributed to the inactivation of cysteine-based phosphatases by ROS [8]. The reversible inactivation of phosphatase—PTEN, by estrogen-induced ROS may be a key component of AKT activation. Together, these data suggest that estrogen effects on brain cells may be in part mediated through ROS signaling biomolecules that regulate estrogen-induced NRF-1 activation. This, in turn, may control the expression of NRF-1-regulatable genes. These data implicated that ROS induced DNA synthesis, increased phosphorylation of kinases, and activated transcription factors, e.g., AP-1, CREB, E2F, NF-κB, and NRF1 of non-genomic pathways which are responsive to oxidants, estrogen and EEDs. Estrogen or EED-induced ROS by increasing genomic instability and by transducing signal through influencing redox sensitive transcription factors may play important role(s) in neurogenesis and synaptogenesis (Figure 2). It is envisioned that estrogen or EED induced ROS mediated signaling is a key complementary mechanism that drives the brain disorders. ROS mediated signaling, however, may occur in the context of other estrogen induced pathways such as ER-mediated signaling. NRF1 regulates targets genes with diverse functions, including cell growth, apoptosis/autophagy, mitochondrial biogenesis, genomic instability, neurogenesis, neuroplasticity, synaptogenesis, and senescence (Figure 14). By activating or repressing the genes involved in cell proliferation, growth suppression, DNA damage/repair, apoptosis/autophagy, angiogenesis, estrogen signaling, neurogenesis, synaptogenesis, and senescence, and inducing a wide range of DNA damage, genomic instability and DNA methylation and transcriptional repression, NRF1 may act as a major regulator of EEDs-induced brain health deficits. Our findings suggest that in addition to estrogen signaling, EED chemicals influencing NRF1-regulated communities of genes across genomic and epigenomic multiple networks may contribute to the development of complex chronic human brain health deficits.

## Figures and Tables

**Table 1:** Epidemiological studies summarizing association between endogenous estrogen, hormonal replacement therapy, phthalates, polychlorinated biphenyls, cadmium, arsenic and manganese and neurodegenerative diseases—Alzheimer’s disease (AD), Parkinson’s disease (PD), amyotrophic lateral sclerosis (ALS), and dementia.

Study Reference	EDC	Year	Epidemiological Study Type	Effect on Brain	Brain Disease
Manly et al., 2000	Endogenous estrogen	2000	Case-control	Lower estradiol levels in women associated with greater risk of AD.	AD
Geerlings et al., 2001	Endogenous estrogen	2001	Prospective cohort	Longer exposure to endogenous estrogen associated with AD and dementia.	Dementia and AD
Schupf et al., 2006	Endogenous estrogen	2006	Prospective-cohort	Women with low bioavailable estrogen were more likely to develop AD.	AD
De Jong et al., 2012	Endogenous estrogen	2012	Case-Control	Longer reproductive time-span and exposure to endogenous estrogen decreases ALS risk.	ALS
Fox et al., 2013	Endogenous estrogen	2013	Retrospective cohort study	Longer duration of endogenous estrogen exposure may have a protective effect against AD risk.	AD
Cereda et al., 2013	Endogenous estrogen	2013	Cross-sectional	Age of PD onset was positively associated with duration of exposure to endogenous estrogens.	PD
Shumaker et al., 2003	Hormonal replacement therapy	2003	Clinical trial	Estrogen progestin HRT increased risk for dementia and did not prevent cognitive impairment.	Dementia
Shumaker et al., 2004	Hormonal replacement therapy	2004	Clinical trial	Estrogen only HRT increased risk of dementia and cognitive impairment.	Dementia
Shao et al., 2012	Hormonal replacement therapy	2012	Cohort study	Increased Alzheimer’s disease risk amongst women who used HRT more than five years after menopause, but observed a decreased risk of AD if used within five years of menopause.	AD
Lundin et al., 2014	Hormonal replacement therapy	2014	Cohort study	Increased in PD risk observed depending on type of hormonal therapy.	PD
Weisskopf et al., 2012	Polychlorinated biphenyls	2012	Nested case-control	PCB exposure not associated with PD development.	PD
Steenland et al., 2006	Polychlorinated biphenyls	2006	Retrospective mortality study	In PCB exposed plant workers, higher death rates from PD were observed in women.	PD
Hatcher-Martin et al., 2012	Polychlorinated biphenyls	2012	Case-control	In post-mortem brain tissue from PD, AD, and controls, PCB levels were higher in PD brain tissue.	PD
Roos et al., 2013	Cadmium	2013	Cross-sectional	Elevated heavy metals, including cadmium, were higher in ALS patients.	ALS
Komatsu et al., 2011	Cadmium	2011	Case-control	Elevated cadmium hair levels were associated with Parkinson-like symptoms.	PD
Park et al., 2014	Arsenic	2014	Cross-sectional	No difference in serum arsenic levels in AD patients and controls.	AD
Hozumi et al., 2011	Manganese	2011	Cross-sectional	Higher levels of manganese found among PD patients.	PD
Koc et al., 2015	Manganese	2015	Cross-sectional	Higher manganese levels found in hair samples of AD patients compared to controls.	AD
Miyake et al., 2011	Manganese	2011	Case-control	In PD patients, no association found with increased manganese intake.	PD
Roos et al., 2013	Manganese	2013	Case-control	Elevated manganese levels observed in ALS patients.	ALS
Kumudini et al., 2014	Manganese	2014	Case-control	No association between blood manganese levels in PD patients’ vs. controls.	PD
Garzillo et al., 2014	Manganese	2014	Case-control	No association between blood manganese levels in ALS patients’ vs. controls.	ALS
Kihira et al., 2015	Manganese	2015	Case-control	Elevated manganese levels in hair observed in ALS patients’ vs. controls.	ALS
Arain et al., 2015	Manganese	2015	Case-control	Higher levels of manganese and aluminum in those suffering from neurodegenerative disease.	Neurodegenerative disease.
Baker et al., 2015	Manganese	2015	Prospective cohort	Low level manganese exposure causes sub-clinical brain changes before symptoms occur.	Neurodegenerative disease

**Table 2:** Epidemiological studies summarizing association between endogenous estrogen, hormonal replacement therapy, bisphenol-A, and phthalates and memory performance, child behavior, and IQ.

Study Reference	EDC	Year	Epidemiological Study Type	Study Population	Measurement of Exposure	Outcome	Results	Confounders	Comments	Brain Health Indicator
Barret-Connor and Goodman-Gruen, 1999	Endogenous estrogen	1999	Cross-sectional	393 females (ages 55 to 89).	Serum estradiol and estrone levels. Bioavailable and total.	Partial correlation (*p*-value) and Linear Regression (β, *p*-value).	No associations between neuropsychological tests and endogenous estrogen exposure.	Smoking status, alcohol use, body mass index, mood, age, education.	Does not account for past exogenous estrogen use, only present use.	Memory performance
Low et al., 2005	Endogenous estrogen	2005	Cross-sectional	760 women (ages 60–64).	Reproductive time period as a surrogate of endogenous estrogen exposure.	Linear regression (β, *p*-value).	No significant associations found between performance on cognitive and memory tests and endogenous estrogen exposure.	Age, education, verbal intelligence, health and mood variables, lifestyle variables, reproductive variables.	Study accounts for exogenous estrogen use.	Memory performance
Heys et al., 2011	Endogenous estrogen	2011	Cross-sectional	11094 women (age > 50 years).	Proxies of endogenous estrogen exposure.	Multivariate Linear regression (*p*-value, 95% CI).	Longer reproductive period associated with higher cognitive delayed recall scores (*p*-value = 0.001; 95% CI, 0.008–0.02) and mini-mental state exam sores (*p*-value < 0.001; 95% CI, 0.04–0.07.	Age, education, childhood and adulthood socio-economic position and physical activity.	Accounts for exogenous estrogen use.	Memory performance
Zimmerman et al., 2011	Endogenous estrogen	2011	Cross-sectional	181 men (mean age = 81 years).	Estradiol and testosterone levels.	Linear Regression (β, *p*-value).	Men with higher levels of total estradiol performed better on verbal memory assessments (β = 0.17, *p*-value < 0.02), compared to men with lower levels of total estradiol.	Age, education, body mass index, and cardiovascular comorbidities.	Accounts for exogenous estrogen use.	Memory performance
**Oral Contraceptives and Memory Performance**										
Beltz et al., 2015	Ethinyl estradiol	2015	Cross-sectional	136 men, 93 normally cycling women, 148 OC users. 18–30 years of age.	History of OC use.	Hierarchal Regression.	Ethinyl estradiol was found to affect memory, but only amongst homogenous groups of OC users.	Age and vocabulary.	Study does not account for endogenous estrogen exposure.	Memory Performance
Egan and Gleason, 2012	Ethinyl estradiol	2012	Cross-sectional	261 cognitively normal women, 40–65 years of age.	History of OC use.	MANCOVA (*u*, CI, *p*-value).	OC users performed better on Visuo-spatial ability (*u* = 0.75, CI 0.23–1.28, *p* = 0.005) and speed and flexibility (*u* = 0.52, CI 0.16–1.04, *p* = 0.007).	Age, years of education, socioeconomic status.	Study compares OC users to non-users.	Memory Performance
Griksiene and Ruksenas, 2011	Ethinyl estradiol	2011	Cross-sectional	43 females, 23 OC users, 20 non-users, ages 19 to 24 years of age.	Salivary 17- β estradiol and progesterone levels.	ANOVA.	OC use negatively affects verbal and spatial abilities.	Not stated.	Does not differentiate between the type of OC.	Memory Performance
**Hormone Replacement Therapy and Memory Performance**										
Resnick et al., 2006	Hormonal replacement therapy	2006	Randomized, double-blind, placebo–controlled clinical trial	1416 postmenopausal women, 65 years of age and older.	Estrogen + Progestin (CEE + MPA) or Placebo.	Change is cognitive function and affect. Generalized Linear Models (*p*-value).	CEE + MPA had a negative effect on verbal memory (*p* < 0.01) and positive effect on figural memory (*p* = 0.012) compared to placebo. No significant influence on positive affect, negative affect, or depressive symptoms.	Age, time at enrollment, education, race, BMI, smoking status, alcoholic drinks/week, history of cardiovascular disease, hypertension, diabetes, prior HT use.	Beneficial and detrimental effects observed.	Memory Performance
Resnick et al., 2009	Hormonal replacement therapy	2009	Randomized, double-blind, placebo-controlled clinical trial	866 postmenopausal women with prior hysterectomy, 65 years of age and older, free of probable dementia.	0.625 mg of CEE daily or placebo.	Annual rates of change in specific cognitive functions and affects. Linear Mixed Models (*p*-value).	Compared with placebo, CEE treatment was associated with lower spatial rotational ability (*p* < 0.01) which diminished over time. CEE did not affect other cognitive functions and affects.	Age, time at enrollment, education, race, BMI, smoking status, alcoholic drinks/week, history of cardiovascular disease, hypertension, diabetes, prior HT use.	No improvements in memory over time.	Memory performance
Almeida et al., 2006	Hormonal replacement therapy.	2006	Randomized, double-blind, placebo-controlled trial	115 Postmenopausal women, 65 years of age and older.	2 mg estradiol or placebo.	Changes in cognitive function and affect.	Higher dosages of estradiol containing HRT treatment did not improve cognitive function.	Age, marital status, education, previous HRT use, daily living, age at menarche, age at menopause, cognitive factors and complaints, plasma estradiol and homocysteine levels.	No significant improvements in memory.	Memory performance.
Pefanco et al., 2007	Hormonal replacement therapy	2007	Randomized, placebo-controlled trial	57 postmenopausal women, 65 years of age and older.	0.20 mg of micronized 17-β estradiol or placebo.	Neuropsychological measures of memory, language, mood, and executive function Repeated Measures Analysis (*p*-value).	No differences were found between ET and placebo on any of the neurocognitive measures or depression instruments, nor were there any differences when the groups were stratified according to age.	Age, education, income, daily living, estradiol levels, estrone levels, hysterectomy.	No significant improvements in memory.	Memory performance.
Viscoli et al., 2005	Hormone replacement therapy.	2005	Randomized, double-blind trial.	644 postmenopausal women.	1 mg of 17-β estradiol or placebo.	Results of MMSE and domain measures. Generalized Linear Models (*p*-values).	Estrogen therapy did not have a significant effect on cognitive measures over time.	Age, race, education level, chronic diseases, prior estrogen therapy use, hysterectomy, depression, stroke.	No significant improvement in memory.	Memory performance.
Yaffe et al., 2006	Hormone replacement therapy.	2006	Randomized, placebo-controlled, double-blind trial	417 postmenopausal women, 60 to 80 years of age.	Weekly transdermal patch of 0.014 mg/day estradiol or placebo.	Results of global cognitive function, verbal and visuospatial memory, language, executive function, and semantic memory. Linear mixed models (*p*-value).	Estrogen therapy did not have a significant effect on cognitive measures over time.	Age, education level, race, BMI, smoking status, depression status, presence of hot flashes, current estradiol level.	No significant improvement in memory.	Memory performance.
**Bisphenol A, Maternal Exposure, and Child Behavior**										
Harley et al., 2013	Bisphenol-A	2013	Prospective Cohort Study	592 mothers and their children, followed prenatally up to 9 years of age.	Urinary BPA concentrations (µg/L). Child and maternal exposure.	Child behavior assessment results. Multivariable linear regression (β, 95% CI).	Prenatal urinary BPA concentrations were associated with increased internalizing problems in boys at age 7 (β = 1.8, 95% CI: 0.3–3.3). Childhood urinary exposure was associated with increased externalizing behavior in girls at age 7 (β = 1.2, 95% CI: 0.3–2.1 and β = 1.0, 95% CI: 0.1–2.0) and increased inattention and hyperactivity behaviors in both boys and girls at age 7.	Maternal age, race/ethnicity, education level, marital status, country of birth, years of US residency, number siblings, family income, maternal depression, pesticide metabolites.	Behavioral results from both teacher and mother reported.	Behavior
Braun et al., 2009	Bisphenol-A	2009	Prospective Cohort Study	249 mothers and their children followed prenatally up to 2 years of age.	Urinary BPA concentrations (µg/L). Child and maternal exposure.	Child behavior assessment. Multivariable linear regression (β, 95% CI).	Prenatal BPA concentrations associated with externalizing scores among females (β = 6.0, 95% CI 0.1–12.0). BPA concentrations at 16 weeks associated with externalizing behavior among all children (β = 2.9, 95% CI 0.2–5.7).	Maternal age, race, education, marital status, SES, maternal depression.	Female appear to be more affected.	Behavior
Yolton et al., 2011	Bisphenol-A	2011	Prospective Cohort Study	350 mothers and their children up to 5 weeks.	Urinary BPA concentrations (ng/mL).	Neurobehavioral outcomes. Multivariable linear regression (*p*-value).	No significant associations between gestational exposure to BPA and infant neurobehavior. Higher BPA exposure associated with greater hypotonia at 26 weeks (*p*-value = 0.09).	Maternal race, household income, marital status, maternal depression, maternal BMI, maternal cotinine levels, infant weight, NICU stay after birth.	Phthalates also measured.	Behavior
Roen et al., 2015	Bisphenol-A	2015	Prospective Cohort Study	250 mothers and their children followed prenatally up to 7–9 years of age.	Urinary BPA concentrations (µg/L).	Neurobehavioral outcomes. Poisson Regression (β, *p*-value).	Among boys, high prenatal BPA concentrations was associated with increase internalizing (β = 0.41, *p* < 0.0001) and externalizing scores (β = 0.40, *p* < 0.0001). High postnatal BPA concentrations was associated with increased internalizing (β = 0.30, *p* = 0.0002) and externalizing scores (β = 0.33, *p* < 0.0001).	Prenatal and postnatal BPA concentration, child age, ethnicity, gestational age, maternal intelligence, maternal education, demoralization, tobacco smoke exposure, and phthalate exposure.	Possible sex specific and timing of exposure mechanism.	Behavior
Braun et al., 2011	Bisphenol-A	2011	Prospective Cohort Study	244 mothers and their children followed from gestation up to 3 years of age.	Urinary BPA concentrations (µg/L).	Neurobehavioral outcomes. Multivariate linear regression (β, 95% CI).	BPA detected in >97% of gestational and childhood samples. Each 10-fold increase in gestational BPA concentrations was associated with more anxious and depressed behavior. The effects were more pronounced in boys.	Mother’s race, education, marital status, household income, maternal depressive behavior, phthalate exposure, tobacco smoke.	Childhood exposure was not significantly associated with behavioral modifications.	Behavior
Miodovnik et al., 2011	Bisphenol-A	2011	Prospective Cohort Study	404 mothers and their children followed from gestation up to 9 years of age.	Urinary BPA concentrations (µg/L).	Neurobehavioral outcomes. General linear models (β, 95% CI).	No associations found with BPA and outcomes.	Maternal age, maternal IQ, marital status, maternal education, child race, sex, child IQ, age at exam, urinary creatinine.	Significant associations found with phthalate exposure.	Behavior
**Phthalates, Behavioral Disorders, and IQ**										
Yolton et al., 2011	Phthalates	2011	Prospective Cohort Study	350 mothers and their children up to 5 weeks.	Urinary phthalate concentrations (ng/mL).	Neurobehavioral outcomes. Multivariable linear regression (*p*-value).	Prenatal exposure to dibutyl phthalate was associated with improved behavioral organization in 5 week old infants characterized by decreased arousal (*p* = 0.04), increased self-regulation (*p* = 0.052), and decreased handling (*p* = 0.02). Prenatal exposure to diethylhexyl phthalate at 26 weeks was associated with nonoptimal reflexes (*p* = 0.02) in male infants.	Maternal race, household income, marital status, maternal depression, maternal BMI, maternal cotinine levels, infant weight, NICU stay after birth.	Results are inconsistent between phthalate types.	Behavior
Miodovnik et al., 2011	Phthalates	2011	Prospective Cohort Study	404 mothers and their children followed from gestation up to 9 years of age.	Urinary phthalate concentrations (µg/L).	Neurobehavioral outcomes. General linear models (β, 95% CI).	Increased phthalate exposure associated with greater social deficits (β = 1.52, 95% CI: 0.25–2.8).	Maternal age, maternal IQ, marital status, maternal education, child race, sex, child IQ, age at exam, urinary creatinine.	Significant associations found with phthalate exposure.	Behavior
Factor-Litvak et al., 2014	Phthalates	2014	Prospective Cohort Study	328 inner-city mothers and their children followed up to 7 years of age.	Urinary phthalate concentrations (ng/mL).	IQ test results. Linear regression models (β, 95% CI).	Prenatal metabolite concentrations of DnBP and DiBP inversely associated with IQ (β = −2.69, 95% CI −4.33, −1.05). Inverse associations seen between IQ, cognitive function, and maternal prenatal metabolite concentrations.	Race, maternal education, marital status, income, parity, gestational age, birth weight, child sex, breastfeeding history, tobacco smoke exposure, prenatal alcohol, hardships during pregnancy, maternal depression.	Phthalate-specific effects were observed.	Child IQ and cognitive function.

**Table 3:** Experimental animal studies showing association between Bisphenol-A, phthalates, polychlorinated biphenyls and neurotoxicity.

Reference	EDC	Study Type	Effects on Brain Health
Bowman et al., 2015	Bisphenol-A	Adolescent male and female adolescent rats	In male rats: Decrease in non-spatial memory and object recognition.
			In both sexes: Decreased spine density on apical and basal dendrites on pyramidal cells in CA1 of the hippocampus.
Eilam-Stock et al., 2012	Bisphenol-A	Adult male rats	Significantly impaired visual and spatial memory.
			Decreased spine density on pyramidal cells in the CA1 and mPFC.
			Decrease expression of PSD-95, increased expression of pCREB.
Elsworth et al., 2015	Bisphenol-A	Adult male vervet monkeys	Decreased working memory and accuracy.
			Decreased excitatory synaptic outputs on dendritic spines of pyramidal neurons in pfc and hippocampus.
Inagaki et al., 2012	Bisphenol-A	Ovariectomized female rats	Exposure to BPA alters E-induced enhancements of spatial and nonspatial memory.
			In the hippocampus, BPA blocked E2 induced increases in basal spine density.
Wang et al., 2014	Bisphenol-A	Postnatal male rats born from BPA exposed female rats	Maternal exposure affected locomotor activity, exploratory habits, emotional behavior.
			Increase reference and working memory errors.
			Decreased mRNA and protein expression of synaptophysin, PSD-95, spinophilin, GluR1, and NMDAR1 in hippocampus.
			Adverse effects on synaptic structure.
Xu et al., 2013	Bisphenol-A	Male and female adult mice	Sex-specific effects of BPA exposure on spatial and passive avoidance memory.
			Reduced synaptic density and adversely affected structure of synaptic interface.
			In hippocampus, down-regulation of snynapsin I, PSD-95, NDMA receptor subunit NR1 and AMPA receptor subunit GluR1 in male mice.
Jang et al., 2012	Bisphenol-A	Postnatal female rats born from BPA expose female rats	Decrease in newly generated cells in the hippocampus.
			Negatively affected memory attention.
			Lower levels of phospho-ERK, BDNF, phosphor-CREB in hippocampus.
			Increased DNA methylation of Crtc1.
Kim et al., 2011	Bisphenol-A	Young adult mice	High dose BPA exposure decreased the number of newly generated cells in the hippocampus, while low dose exposure had the opposite effect.
			High dose BPA exposure was shown to impair learning and memory performance significantly.
Diaz Weinstein et al., 2013	Bisphenol-A	Adolescent male and female rats	Decreased spatial memory.
			Adverse locomotor activity.
			Increased anxiety.
Elsworth et al., 2015	Bisphenol-A	Pregnant female rhesus monkeys	Abnormal fetal brain development.
			Fetuses of female monkeys showed a decrease in midbrain dopamine neurons and a reduction in spine synapses in the CA1 region of the hippocampus.
Johnson et al., 2015	Bisphenol-A	Pregnant female rats and offspring	In the offspring: Developmental exposure disrupted navigational learning and navigational memory.
Kumar and Kumar Thakur 2014	Bisphenol-A	Pregnant female mice and male offspring	In the offspring: Impaired spatial memory.
			Increased dendritic spine density in the cerebral cortex and hippocampus.
			Upregulation of synaptic proteins Nrxn1 and Nlgn3.
Matsuda et al., 2013	Bisphenol-A	Pregnant female mice and offspring	In the offspring: Enhanced fear memory.
			Increased serotonin metabolites.
			Increased expression of Tph2, Slc6a4, and Maoa.
Xu et al., 2014	Bisphenol-A	Pregnant female rats and offspring	In the offspring: Interference with estrogen receptor signaling in the developing hippocampus.
Zhang et al., 2014	Bisphenol-A	Adult male mice	Enhanced the acquisition and retention of fear memory by the increased levels of histone acetylation, NMDA receptor, and phosphorylation of the ERK1/2 in the hippocampus of male mice.
Stump et al., 2010	Bisphenol-A	Pregnant female rats and offspring	No adverse effects reported.
Ishido et al., 2011	Bisphenol-A	Postnatal male mice	Apparent hyperactivity observed.
Viberg et al., 2011	Bisphenol-A	Postnatal mice	Alterations in spontaneous behavior and cognitive function observed.
Kimura et al., 2015	Bisphenol-A	Prenatal mice	In utero exposure to BPA reduced spine densities in the hippocampal CA1 region of the brain in prenatal mice.
Kim et al., 2009	Bisphenol-A	Neonatal and postnatal mice	Stimulation of neuronal differentiation and possible disruption of neonatal brain development observed.
Dai et al., 2015	Phthalates	Young mice	Negatively affected locomotion activity and memory.
Betz et al., 2013	Phthalates	Young mice	Negatively affects learning and social behavior.
Li et al., 2013	Phthalates	Young mice	Induced apoptotic cell death, synaptic loss, and synaptic dysfunction.
Elnar et al., 2015	Phthalates	Young mice	Early exposure to PCBs induced neuronal susceptibility to amyloid stress. Lower expression of synaptic proteins.
Reilly et al., 2015	PCBs	Young mice	Negatively affects social behavior.
Zahara et al., 2015	PCBs	Juvenile Avians	Reduction in learning ability.
Hilgier et al., 2012	PCBs	Adult Rats	Exposure resulted in neuronal injury and loss.
Lee et al., 2012	PCBs	Adult mice	Exposure resulted in hyperactivity and dopaminergic neuron degeneration.

**Table 4:** Top 10 enriched pathways for E2 and NRF1-Target genes associated with estrogenic endocrine disruptors—BPA, PCB, phthalates, As, Cd, and Mn.

**17 β-Estradiol (E2) Interacting NRF1 Target Genes**		
**KEGG Pathway**	**Number of Genes**	**Annotated Genes**
Disease	86	*ADCY9|AKT2|ALDOA|AP1S1|CCNT2|CDC42|CDK1|CDKN2B|CHMP2A|CHMP2B|CHMP5|CREB1|CSK|CSNK1A1|CTBP1|CYB5A|ENO1|EPS15L1|ERCC2|EXT2|FASN|FZD4|GLB1|GTF2E2|HDAC2|HDAC3|HDAC4|HMMR|HRAS|HSP90AA1|IRS1|MAP2K7|MKNK1|MMADHC|MTHFD1|NAMPT|NPM1|NUP107|NUP50|OS9|P4HB|PAPSS1|PFKFB4|PIP5K1B|PLCG1|POLR2A|POM121|PPIA|PRKAR2A|PSENEN|PYGL|RAC1|RAE1|RAF1|RAN|RBX1|RHOA|RPL10|RPL13A|RPL14|RPL36|RPS13|RPS16|RPS21|RPS29|RPS9|SHC1|SLC25A10|SLC37A4|SMARCA4|SNW1|SOS1|SRC|STUB1|STX1A|TAF12|TAF5|TCEB2|TGIF1|TNKS2|TPR|UBA52|WNT5A|XRCC5|XRCC6|YWHAZ*
Metabolism	86	*ACADM|ADCY9|ADI1|AGPAT5|AKR7A2|ALAS1|ALDOA|AMACR|ATP5O|AUH|BCAT1|BCAT2|BSG|CBR1|COX5B|COX6C|CTPS1|CYB5A|CYCS|DTYMK|ELOVL4|ENO1|ERCC2|ETFB|ETFDH|EXT2|FASN|FDPS|GCLC|GLB1|GNG5|GPD1L|GSR|GSS|GSTM3|HDAC3|HMMR|HSD17B4|HSP90AA1|INPP5A|INPP5K|IP6K2|LDHA|LPCAT3|LSS|MGST3|MMADHC|MPC2|MTHFD1|NAMPT|NDUFA3|NDUFB4|NUBP1|NUP107|NUP50|OAZ2|ODC1|P4HB|PAICS|PAPSS1|PDHB|PFKFB4|PIP5K1B|PLA2G12A|PLCG1|PLD1|POM121|PPAT|PRKAR2A|PSAP|PYGL|RAE1|RAN|SDHA|SGMS1|SIN3B|SLC16A1|SLC25A10|SLC37A4|SLC44A2|STX1A|SUCLA2|TPMT|TPR|UBA52|VAPB*
Gene Expression	77	*CCNT2|CDKN2B|CNOT1|DHX9|DNMT1|EEF1A1|EEF1D|EEF1G|EIF2S1|EIF2S2|EIF3K|EIF4E|EIF4G1|ERCC2|ESRRA|EXOSC2|EZH2|FARSB|GEMIN4|GTF2E2|HARS|HDAC2|HNRNPA1|HNRNPH1|HNRNPU|HSPA8|LSM2|MARS2|MED20|MED4|NR1D1|PCBP2|POLR1A|POLR2A|POLRMT|PPA1|PTBP1|RAN|RBBP4|RBBP7|RNPS1|RPL10|RPL13A|RPL14|RPL36|RPS13|RPS16|RPS21|RPS29|RPS9|RUNX2|SAP18|SARS|SEC61G|SET|SIN3B|SNW1|SRRM1|SRSF1|TAF12|TAF5|TARS|TCEB2|TGIF1|THRA|TRAM1|U2AF1|UBA52|UPF1|UPF2|VARS2|XPO5|YWHAZ|ZNF610|ZNF658|ZNF711|ZNF750*
Signal Transduction	72	*ABI1|ADCY9|AKT2|ARHGDIA|BRK1|CASP2|CCNT2|CDC42|CDK1|CDKN2B|CREB1|CRK|CRKL|CSK|CSNK1A1|CTBP1|DAAM1|DGKH|E2F1|EIF4E|EIF4G1|EPS15L1|FSTL3|FZD4|GNG5|GPR37|HDAC2|HDAC3|HDAC4|HRAS|HSP90AA1|IRS1|JAK2|LFNG|MAPK7|MEF2A|MKNK1|OS9|P4HB|PFN1|PIP5K1B|PLCG1|PRDM4|PRKAR2A|PSAP|PSENEN|PTCH1|PTK2|RAC1|RAF1|RBX1|RELA|RHOA|RIPK2|ROCK1|RPS6KA5|RPS6KB1|SHC1|SMARCA4|SNW1|SOCS1|SOS1|SRC|STARD13|STUB1|TGIF1|THBS2|TNKS2|TRIO|UBA52|WNT5A|YWHAZ*
Immune System	67	*ABI1|ADCY9|AKT2|ANAPC1|ANAPC11|AP1S1|ARPC2|ARPC5|BRK1|CASP2|CDC34|CDC42|CDK1|CREB1|CRK|CRKL|CSK|CTSD|DHX9|DYNLL1|EIF4E|EIF4G1|FADD|FZR1|HRAS|HSP90AA1|IP6K2|IRS1|JAK2|KIF18A|KIF4A|MAP2K7|MAPK7|MEF2A|NUP107|NUP50|PCBP2|PELI1|PLCG1|PLD1|POM121|PRKAR2A|PRKDC|PTK2|PVR|RAC1|RAE1|RAF1|RBX1|RELA|RIPK2|RNF19B|RPS6KA5|SEC61G|SHC1|SOCS1|SOS1|SRC|STUB1|TCEB2|TPR|TUBB4B|UBA52|UBE2D4|XRCC5|XRCC6|YWHAZ*
Cell Cycle	62	*ANAPC1|ANAPC11|APITD1|ATM|AURKA|AURKB|BUB1|CCNB1|CCNE1|CDC7|CDK1|CDKN2B|CDKN2D|CDT1|CENPN|CKS1B|CLASP2|DKC1|DSN1|DYNLL1|E2F1|ERCC6L|FBXO5|FZR1|GOLGA2|HSP90AA1|KIF18A|LIN37|MAD2L1|MCM2|MCM4|MCM6|MLH3|NEK2|NPM1|NUP107|NUP50|OIP5|ORC6|PCNA|PLK4|POLA1|POM121|PPP1R12A|RAB1B|RAD50|RAE1|RBBP4|RBBP7|RBBP8|RBL2|RSF1|SDCCAG8|SET|SMC4|SPC24|SPDL1|TPR|TUBB|TUBB4B|UBA52|ZWINT*
Metabolic pathways	62	*ACADM|ADI1|ALAS1|ALDOA|AMACR|ATP5O|AUH|BCAT1|BCAT2|CBR1|COX5B|COX6A2|COX6C|CTPS1|DAD1|DGKH|DNMT1|DTYMK|ENO1|EXT2|FASN|FBL|FDPS|FUT8|GAA|GCLC|GFPT1|GLB1|GSS|HSD17B4|INPP5A|INPP5K|LAP3|LDHA|LSS|MECR|MTHFD1|NDUFA3|NDUFB4|ODC1|PAICS|PAPSS1|PDHB|PGAM5|PGAP1|PIGA|PIP5K1B|PLA2G12A|PLCG1|PLD1|POLA1|POLE4|POLG2|POLR1A|POLR2A|PPAT|SDHA|SGMS1|SHMT2|SUCLA2|SUFU|TGDS*
Metabolism of proteins	48	*B3GNTL1|CCT3|CCT6A|CCT8|CTSD|DAD1|DDIT3|DNAJB9|DNAJC3|DOHH|EEF1A1|EEF1D|EEF1G|EIF2S1|EIF2S2|EIF3K|EIF4E|EIF4G1|EIF5A|FBXW5|FUT8|GFPT1|GLB1|GRPEL2|HSPA9|HSPD1|IGFBP3|MANEA|PFDN1|PGAP1|PIGA|RPL10|RPL13A|RPL14|RPL36|RPS13|RPS16|RPS21|RPS29|RPS9|SEC61G|SHC1|STX1A|THBS2|TRAM1|TSPYL2|TUBB4B|UBA52*
Developmental Biology	42	*ABLIM2|AKT2|ARPC2|ARPC5|CDC42|CDK1|CLASP2|CREB1|CRMP1|CTNNA2|DPYSL2|HDAC3|HRAS|HSP90AA1|HSPA8|ITGA1|KIF4A|MED11|MED18|MED20|MED4|MEF2A|MYH10|MYH14|NRTN|PFN1|PLCG1|PSENEN|PTK2|RAC1|RAF1|RHOA|ROCK1|RPS6KA5|RPS6KA6|SCN3B|SDCBP|SIAH2|SOS1|SPTAN1|SRC|TRIO|TUBB4B*
Mitotic M-M/G1 phases	35	*ANAPC1|ANAPC11|APITD1|AURKB|BUB1|CCNB1|CDC7|CDK1|CDT1|CENPN|CLASP2|DSN1|ERCC6L|FBXO5|GOLGA2|KIF18A|MAD2L1|MCM2|MCM4|MCM6|NUP107|NUP50|ORC6|POLA1|POM121|RAB1B|RAE1|SET|SMC4|SPC24|SPDL1|TPR|TUBB4B|UBA52|ZWINT*
**Ethinyl Estradiol Interacting Common E2- and NRF1-Target Genes**		
**KEGG Pathway**	**Number of Genes**	**Annotated Genes**
Metabolism	49	*ACADM|ADCY9|AGPAT5|ALAS1|ALDOA|ATP5O|BCAT1|BSG|CBR1|COX5B|COX6C|CTPS1|CYB5A|CYCS|ENO1|ERCC2|ETFDH|EXT2|FASN|FDPS|GCLC|GLB1|GSR|GSS|GSTM3|HDAC3|HSD17B4|HSP90AA1|INPP5A|LDHA|LSS|MGST3|MTHFD1|NAMPT|NUP50|ODC1|P4HB|PAPSS1|PDHB|PPAT|PSAP|PYGL|RAE1|RAN|SGMS1|SIN3B|SLC16A1|SLC25A10|SLC37A4*
Disease	48	*ADCY9|AKT2|ALDOA|AP1S1|CDC42|CDK1|CHMP2A|CREB1|CSK|CSNK1A1|CYB5A|ENO1|ERCC2|EXT2|FASN|GLB1|GTF2E2|HDAC2|HDAC3|HRAS|HSP90AA1|MAP2K7|MTHFD1|NAMPT|NPM1|NUP50|OS9|P4HB|PAPSS1|PSENEN|PYGL|RAE1|RAN|RBX1|RHOA|RPL36|RPS16|RPS9|SLC25A10|SLC37A4|SMARCA4|STUB1|TAF12|TAF5|TCEB2|WNT5A|XRCC5|YWHAZ*
Gene Expression	43	*DNMT1|EEF1A1|EEF1G|EIF2S1|EIF2S2|EIF3K|EIF4E|ERCC2|EZH2|FARSB|GTF2E2|HARS|HDAC2|HNRNPA1|HNRNPH1|HNRNPU|HSPA8|MED4|NR1D1|PCBP2|POLR1A|POLRMT|RAN|RBBP4|RBBP7|RNPS1|RPL36|RPS16|RPS9|SAP18|SARS|SEC61G|SET|SIN3B|SRSF1|TAF12|TAF5|TARS|TCEB2|THRA|UPF1|VARS2|YWHAZ*
Immune System	36	*ABI1|ADCY9|AKT2|ANAPC1|ANAPC11|AP1S1|ARPC2|ARPC5|CASP2|CDC42|CDK1|CREB1|CRK|CSK|CTSD|DYNLL1|EIF4E|FADD|FZR1|HRAS|HSP90AA1|MAP2K7|MAPK7|NUP50|PCBP2|PTK2|PVR|RAE1|RBX1|SEC61G|SOCS1|STUB1|TCEB2|TUBB4B|XRCC5|YWHAZ*
Cell Cycle	35	*ANAPC1|ANAPC11|AURKB|BUB1|CCNB1|CCNE1|CDC7|CDK1|CDT1|CKS1B|DYNLL1|E2F1|FZR1|GOLGA2|HSP90AA1|LIN37|MAD2L1|MCM2|MCM4|MCM6|NPM1|NUP50|ORC6|PCNA|PLK4|PPP1R12A|RAE1|RBBP4|RBBP7|RBBP8|RSF1|SET|SPC24|SPDL1|TUBB4B*
Metabolic pathways	33	*ACADM|ALAS1|ALDOA|ATP5O|BCAT1|CBR1|COX5B|COX6C|CTPS1|DNMT1|ENO1|EXT2|FASN|FDPS|GCLC|GLB1|GSS|HSD17B4|INPP5A|LDHA|LSS|MECR|MTHFD1|ODC1|PAPSS1|PDHB|PGAM5|PIGA|POLE4|POLG2|POLR1A|PPAT|SGMS1*
Signal Transduction	33	*ABI1|ADCY9|AKT2|CASP2|CDC42|CDK1|CREB1|CRK|CSK|CSNK1A1|E2F1|EIF4E|HDAC2|HDAC3|HRAS|HSP90AA1|MAPK7|OS9|P4HB|PFN1|PRDM4|PSAP|PSENEN|PTCH1|PTK2|RBX1|RHOA|SMARCA4|SOCS1|STUB1|TRIO|WNT5A|YWHAZ*
Metabolism of proteins	28	*B3GNTL1|CCT3|CCT6A|CCT8|CTSD|DDIT3|DNAJC3|EEF1A1|EEF1G|EIF2S1|EIF2S2|EIF3K|EIF4E|EIF5A|FBXW5|GLB1|GRPEL2|HSPA9|HSPD1|IGFBP3|PFDN1|PIGA|RPL36|RPS16|RPS9|SEC61G|TSPYL2|TUBB4B*
Developmental Biology	25	*AKT2|ARPC2|ARPC5|CDC42|CDK1|CREB1|CTNNA2|HDAC3|HRAS|HSP90AA1|HSPA8|ITGA1|MED11|MED4|MYH10|NRTN|PFN1|PSENEN|PTK2|RHOA|RPS6KA6|SDCBP|SIAH2|TRIO|TUBB4B*
**Bisphenol A Interacting Common E2- and NRF1-Target Genes**		
**KEGG Pathway**	**Number of Genes**	**Annotated Genes**
Metabolism	85	*ACADM|ADCY9|ADI1|AGPAT5|AKR7A2|ALAS1|ALDOA|AMACR|ATP5O|BCAT1|BCAT2|BSG|CBR1|COX5B|COX6C|CTPS1|CYB5A|CYCS|DTYMK|ELOVL4|ENO1|ERCC2|ETFB|ETFDH|EXT2|FASN|FDPS|GCLC|GLB1|GNG5|GPD1L|GSR|GSS|GSTM3|HDAC3|HMMR|HSD17B4|HSP90AA1|INPP5A|INPP5K|IP6K2|LDHA|LPCAT3|LSS|MGST3|MMADHC|MPC2|MTHFD1|NAMPT|NDUFA3|NDUFB4|NUBP1|NUP107|NUP50|OAZ2|ODC1|P4HB|PAICS|PAPSS1|PDHB|PFKFB4|PIP5K1B|PLA2G12A|PLCG1|PLD1|POM121|PPAT|PRKAR2A|PSAP|PYGL|RAE1|RAN|SDHA|SGMS1|SIN3B|SLC16A1|SLC25A10|SLC37A4|SLC44A2|STX1A|SUCLA2|TPMT|TPR|UBA52|VAPB*
Disease	84	*ADCY9|AKT2|ALDOA|AP1S1|CCNT2|CDC42|CDK1|CDKN2B|CHMP2A|CHMP2B|CHMP5|CREB1|CSK|CSNK1A1|CTBP1|CYB5A|ENO1|ERCC2|EXT2|FASN|FZD4|GLB1|GTF2E2|HDAC2|HDAC3|HDAC4|HMMR|HRAS|HSP90AA1|IRS1|MAP2K7|MKNK1|MMADHC|MTHFD1|NAMPT|NPM1|NUP107|NUP50|OS9|P4HB|PAPSS1|PFKFB4|PIP5K1B|PLCG1|POLR2A|POM121|PPIA|PRKAR2A|PSENEN|PYGL|RAC1|RAE1|RAF1|RAN|RBX1|RHOA|RPL13A|RPL14|RPL36|RPS13|RPS16|RPS21|RPS29|RPS9|SHC1|SLC25A10|SLC37A4|SMARCA4|SNW1|SOS1|SRC|STUB1|STX1A|TAF12|TAF5|TCEB2|TGIF1|TNKS2|TPR|UBA52|WNT5A|XRCC5|XRCC6|YWHAZ*
Gene Expression	71	*CCNT2|CDKN2B|CNOT1|DHX9|DNMT1|EEF1A1|EEF1D|EEF1G|EIF2S1|EIF2S2|EIF3K|EIF4E|EIF4G1|ERCC2|ESRRA|EXOSC2|EZH2|FARSB|GEMIN4|GTF2E2|HARS|HDAC2|HNRNPA1|HNRNPH1|HNRNPU|HSPA8|LSM2|MARS2|MED20|MED4|NR1D1|PCBP2|POLR1A|POLR2A|PPA1|PTBP1|RAN|RBBP4|RBBP7|RNPS1|RPL13A|RPL14|RPL36|RPS13|RPS16|RPS21|RPS29|RPS9|RUNX2|SAP18|SARS|SEC61G|SET|SIN3B|SNW1|SRRM1|SRSF1|TAF12|TAF5|TARS|TCEB2|TGIF1|THRA|TRAM1|U2AF1|UBA52|UPF1|UPF2|XPO5|YWHAZ|ZNF711*
Signal Transduction	68	*ABI1|ADCY9|AKT2|ARHGDIA|BRK1|CASP2|CCNT2|CDC42|CDK1|CDKN2B|CREB1|CRK|CRKL|CSK|CSNK1A1|CTBP1|DAAM1|DGKH|E2F1|EIF4E|EIF4G1|FSTL3|FZD4|GNG5|GPR37|HDAC2|HDAC3|HDAC4|HRAS|HSP90AA1|IRS1|JAK2|LFNG|MAPK7|MKNK1|OS9|P4HB|PFN1|PIP5K1B|PLCG1|PRKAR2A|PSAP|PSENEN|PTCH1|PTK2|RAC1|RAF1|RBX1|RELA|RHOA|RIPK2|ROCK1|RPS6KA5|RPS6KB1|SHC1|SMARCA4|SNW1|SOCS1|SOS1|SRC|STARD13|STUB1|TGIF1|THBS2|TNKS2|UBA52|WNT5A|YWHAZ*
Immune System	66	*ABI1|ADCY9|AKT2|ANAPC1|ANAPC11|AP1S1|ARPC2|ARPC5|BRK1|CASP2|CDC34|CDC42|CDK1|CREB1|CRK|CRKL|CSK|CTSD|DHX9|DYNLL1|EIF4E|EIF4G1|FADD|FZR1|HRAS|HSP90AA1|IP6K2|IRS1|JAK2|KIF18A|KIF4A|MAP2K7|MAPK7|NUP107|NUP50|PCBP2|PELI1|PLCG1|PLD1|POM121|PRKAR2A|PRKDC|PTK2|PVR|RAC1|RAE1|RAF1|RBX1|RELA|RIPK2|RNF19B|RPS6KA5|SEC61G|SHC1|SOCS1|SOS1|SRC|STUB1|TCEB2|TPR|TUBB4B|UBA52|UBE2D4|XRCC5|XRCC6|YWHAZ*
Cell Cycle	61	*ANAPC1|ANAPC11|ATM|AURKA|AURKB|BUB1|CCNB1|CCNE1|CDC7|CDK1|CDKN2B|CDKN2D|CDT1|CENPN|CKS1B|CLASP2|DKC1|DSN1|DYNLL1|E2F1|ERCC6L|FBXO5|FZR1|GOLGA2|HSP90AA1|KIF18A|LIN37|MAD2L1|MCM2|MCM4|MCM6|MLH3|NEK2|NPM1|NUP107|NUP50|OIP5|ORC6|PCNA|PLK4|POLA1|POM121|PPP1R12A|RAB1B|RAD50|RAE1|RBBP4|RBBP7|RBBP8|RBL2|RSF1|SDCCAG8|SET|SMC4|SPC24|SPDL1|TPR|TUBB|TUBB4B|UBA52|ZWINT*
Metabolic pathways	58	*ACADM|ADI1|ALAS1|ALDOA|AMACR|ATP5O|BCAT1|BCAT2|CBR1|COX5B|COX6C|CTPS1|DAD1|DGKH|DNMT1|DTYMK|ENO1|EXT2|FASN|FBL|FDPS|FUT8|GAA|GCLC|GFPT1|GLB1|GSS|HSD17B4|INPP5A|INPP5K|LDHA|LSS|MECR|MTHFD1|NDUFA3|NDUFB4|ODC1|PAICS|PAPSS1|PDHB|PGAM5|PGAP1|PIGA|PIP5K1B|PLA2G12A|PLCG1|PLD1|POLA1|POLG2|POLR1A|POLR2A|PPAT|SDHA|SGMS1|SHMT2|SUCLA2|SUFU|TGDS*
Metabolism of proteins	47	*B3GNTL1|CCT3|CCT6A|CCT8|CTSD|DAD1|DDIT3|DNAJB9|DNAJC3|DOHH|EEF1A1|EEF1D|EEF1G|EIF2S1|EIF2S2|EIF3K|EIF4E|EIF4G1|EIF5A|FBXW5|FUT8|GFPT1|GLB1|GRPEL2|HSPA9|HSPD1|IGFBP3|MANEA|PFDN1|PGAP1|PIGA|RPL13A|RPL14|RPL36|RPS13|RPS16|RPS21|RPS29|RPS9|SEC61G|SHC1|STX1A|THBS2|TRAM1|TSPYL2|TUBB4B|UBA52*
Developmental Biology	41	*ABLIM2|AKT2|ARPC2|ARPC5|CDC42|CDK1|CLASP2|CREB1|CRMP1|CTNNA2|DPYSL2|HDAC3|HRAS|HSP90AA1|HSPA8|ITGA1|KIF4A|MED11|MED18|MED20|MED4|MYH10|MYH14|NRTN|PFN1|PLCG1|PSENEN|PTK2|RAC1|RAF1|RHOA|ROCK1|RPS6KA5|RPS6KA6|SCN3B|SDCBP|SIAH2|SOS1|SPTAN1|SRC|TUBB4B*
Pathways in cancer	34	*AKT2|ARAF|CCNE1|CDC42|CDKN2B|CKS1B|CKS2|CRK|CRKL|CTBP1|CTNNA2|CYCS|E2F1|FADD|FZD4|HDAC2|HRAS|HSP90AA1|MSH2|PLCG1|PLD1|PTCH1|PTK2|RAC1|RAF1|RASSF1|RBX1|RELA|RHOA|SOS1|SUFU|TCEB2|TPR|WNT5A*
**Dibutyl Phthalate Interacting Common E2- and NRF1-Target Genes**		
**KEGG Pathway**	**Number of Genes**	**Annotated Genes**
Metabolism	56	*ACADM|ADCY9|ADI1|AKR7A2|ALAS1|ALDOA|AMACR|ATP5O|BCAT1|BCAT2|BSG|CBR1|CYB5A|CYCS|DTYMK|ENO1|ETFB|ETFDH|EXT2|FASN|FDPS|GCLC|GLB1|GNG5|GSR|GSS|HDAC3|HMMR|HSD17B4|HSP90AA1|INPP5A|IP6K2|LDHA|LPCAT3|LSS|MGST3|MTHFD1|NAMPT|NDUFA3|NDUFB4|NUBP1|NUP50|OAZ2|ODC1|PAPSS1|PLA2G12A|PLD1|POM121|PRKAR2A|PYGL|RAE1|RAN|SDHA|SGMS1|SLC25A10|UBA52*
Disease	45	*ADCY9|ALDOA|CCNT2|CDK1|CREB1|CSNK1A1|CTBP1|CYB5A|ENO1|EXT2|FASN|GLB1|HDAC3|HMMR|HSP90AA1|MTHFD1|NAMPT|NPM1|NUP50|PAPSS1|POLR2A|POM121|PRKAR2A|PYGL|RAE1|RAF1|RAN|RHOA|RPL13A|RPL14|RPL36|RPS13|RPS21|RPS29|SLC25A10|SMARCA4|SNW1|SRC|STUB1|TCEB2|TGIF1|TNKS2|UBA52|WNT5A|XRCC5*
Gene Expression	39	*CCNT2|DNMT1|EEF1A1|EEF1D|EIF2S1|EIF2S2|EIF4E|EIF4G1|ESRRA|EXOSC2|EZH2|GEMIN4|HNRNPA1|HNRNPU|HSPA8|LSM2|MED20|POLR1A|POLR2A|PTBP1|RAN|RBBP4|RNPS1|RPL13A|RPL14|RPL36|RPS13|RPS21|RPS29|SET|SNW1|SRSF1|TARS|TCEB2|TGIF1|THRA|U2AF1|UBA52|XPO5*
Metabolic pathways	38	*ACADM|ADI1|ALAS1|ALDOA|AMACR|ATP5O|BCAT1|BCAT2|CBR1|COX6A2|DNMT1|DTYMK|ENO1|EXT2|FASN|FBL|FDPS|GCLC|GFPT1|GLB1|GSS|HSD17B4|INPP5A|LDHA|LSS|MTHFD1|NDUFA3|NDUFB4|ODC1|PAPSS1|PGAM5|PLA2G12A|PLD1|POLE4|POLR1A|POLR2A|SDHA|SGMS1*
Cell Cycle	34	*ANAPC1|ANAPC11|APITD1|ATM|AURKB|CCNB1|CCNE1|CDC7|CDK1|CDKN2D|CDT1|CENPN|CKS1B|CLASP2|FBXO5|FZR1|HSP90AA1|MCM2|MCM4|MCM6|NPM1|NUP50|OIP5|ORC6|PCNA|POM121|PPP1R12A|RAE1|RBBP4|SET|SPC24|SPDL1|UBA52|ZWINT*
Signal Transduction	32	*ADCY9|ARHGDIA|CCNT2|CDK1|CREB1|CSNK1A1|CTBP1|DAAM1|EIF4E|EIF4G1|GNG5|HDAC3|HSP90AA1|JAK2|LFNG|MEF2A|PRDM4|PRKAR2A|PTCH1|RAF1|RHOA|SMARCA4|SNW1|SOCS1|SRC|STUB1|TGIF1|THBS2|TNKS2|TRIO|UBA52|WNT5A*
Immune System	31	*ADCY9|ANAPC1|ANAPC11|ARPC2|ARPC5|CDK1|CREB1|CTSD|EIF4E|EIF4G1|FADD|FZR1|HSP90AA1|IP6K2|JAK2|MEF2A|NUP50|PLD1|POM121|PRKAR2A|PRKDC|PVR|RAE1|RAF1|RNF19B|SOCS1|SRC|STUB1|TCEB2|UBA52|XRCC5*
Metabolism of proteins	25	*CCT3|CTSD|DDIT3|DNAJB9|DNAJC3|EEF1A1|EEF1D|EIF2S1|EIF2S2|EIF4E|EIF4G1|FBXW5|GFPT1|GLB1|HSPA9|HSPD1|PFDN1|RPL13A|RPL14|RPL36|RPS13|RPS21|RPS29|THBS2|UBA52*
Mitotic M-M/G1 phases	23	*ANAPC1|ANAPC11|APITD1|AURKB|CCNB1|CDC7|CDK1|CDT1|CENPN|CLASP2|FBXO5|MCM2|MCM4|MCM6|NUP50|ORC6|POM121|RAE1|SET|SPC24|SPDL1|UBA52|ZWINT*
Developmental Biology	21	*ARPC2|ARPC5|CDK1|CLASP2|CREB1|CTNNA2|DPYSL2|HDAC3|HSP90AA1|HSPA8|ITGA1|MED20|MEF2A|MYH10|NRTN|RAF1|RHOA|SCN3B|SDCBP|SRC|TRIO*
**Diethylhexyl Phthalate Interacting Common E2- and NRF1-Target Genes**		
**KEGG Pathway**	**Number of Genes**	**Annotated Genes**
Metabolism	21	*ACADM|ALAS1|ALDOA|BCAT2|BSG|CYCS|FASN|FDPS|GSR|GSTM3|HSD17B4|HSP90AA1|LDHA|LSS|MMADHC|NDUFA3|ODC1|PAPSS1|PLD1|PYGL|SLC37A4*
Metabolic pathways	15	*ACADM|ALAS1|ALDOA|BCAT2|DNMT1|FASN|FDPS|HSD17B4|LDHA|LSS|NDUFA3|ODC1|PAPSS1|PLD1|POLE4*
Disease	15	*AKT2|ALDOA|FASN|HDAC2|HDAC4|HSP90AA1|IRS1|MMADHC|NPM1|PAPSS1|PYGL|RBX1|RPL36|RPS13|SLC37A4*
Immune System	12	*AKT2|ANAPC11|CDC34|DYNLL1|EIF4G1|HSP90AA1|IRS1|KIF18A|PLD1|RBX1|SEC61G|TUBB4B*
Metabolism of proteins	11	*CCT3|CCT6A|DNAJB9|EIF4G1|GRPEL2|HSPA9|HSPD1|RPL36|RPS13|SEC61G|TUBB4B*
Cell Cycle	9	*ANAPC11|CCNB1|CCNE1|DYNLL1|HSP90AA1|KIF18A|NPM1|TUBB4B|ZWINT*
Cellular responses to stress	8	*ANAPC11|CBX2|CCNE1|CYCS|GSR|HSP90AA1|HSPA8|RBX1*
Pathways in cancer	8	*AKT2|CCNE1|CYCS|HDAC2|HSP90AA1|MSH2|PLD1|RBX1*
Developmental Biology	8	*AKT2|DPYSL2|HSP90AA1|HSPA8|ITGA1|SCN3B|SIAH2|TUBB4B*
Cell cycle	5	*ANAPC11|CCNB1|CCNE1|HDAC2|RBX1*
**Polychlorinated Biphenyls Interacting Common E2- and NRF1-Target Gene**		
**KEGG Pathway**	**Number of Genes**	**Annotated Genes**
Cell Cycle	13	*AURKA|AURKB|CDK1|CDT1|CENPN|CKS1B|FBXO5|MCM2|MCM6|PCNA|PLK4|SMC4|ZWINT*
Mitotic M-M/G1 phases	9	*AURKB|CDK1|CDT1|CENPN|FBXO5|MCM2|MCM6|SMC4|ZWINT*
DNA Replication	4	*CDT1|MCM2|MCM6|PCNA*
Cell cycle	4	*CDK1|MCM2|MCM6|PCNA*
DNA replication	3	*MCM2|MCM6|PCNA*
DNA replication and repair	3	*CDK1|PCNA|UNG*
Pancreatic cancer	3	*ARAF|CDC42|RAF1*
**Cadmium Interacting Common E2- and NRF1-Target Genes**		
**KEGG Pathway**	**Number of Genes**	**Annotated Genes**
Metabolism	26	*ACADM|ALDOA|AUH|BCAT1|COX5B|COX6C|CYCS|DTYMK|ENO1|FASN|GCLC|GSR|GSS|GSTM3|HSD17B4|HSP90AA1|INPP5K|LDHA|MGST3|OAZ2|PAICS|RAN|SDHA|SGMS1|SLC16A1|SLC37A4*
Gene Expression	25	*CCNT2|CDKN2B|DNMT1|EEF1A1|EEF1D|EEF1G|EXOSC2|HARS|HNRNPA1|HNRNPH1|HSPA8|LSM2|MARS2|MED20|RAN|RPL10|RPL13A|RPL14|RUNX2|SRRM1|TAF12|TCEB2|THRA|XPO5|YWHAZ*
Disease	24	*ALDOA|CCNT2|CDC42|CDK1|CDKN2B|CREB1|CTBP1|ENO1|FASN|HSP90AA1|IRS1|OS9|PPIA|RAC1|RAF1|RAN|RPL10|RPL13A|RPL14|SHC1|SLC37A4|TAF12|TCEB2|YWHAZ*
Signal Transduction	21	*ARHGDIA|CCNT2|CDC42|CDK1|CDKN2B|CREB1|CTBP1|E2F1|FSTL3|GPR37|HSP90AA1|IRS1|OS9|PTK2|RAC1|RAF1|RELA|ROCK1|RPS6KB1|SHC1|YWHAZ*
Metabolic pathways	20	*ACADM|ALDOA|AUH|BCAT1|COX5B|COX6C|DNMT1|DTYMK|ENO1|FASN|GAA|GCLC|GSS|HSD17B4|INPP5K|LDHA|PAICS|SDHA|SGMS1|SHMT2*
Immune System	18	*CDC42|CDK1|CREB1|CTSD|HSP90AA1|IRS1|KIF4A|PELI1|PTK2|PVR|RAC1|RAF1|RELA|RNF19B|SHC1|TCEB2|UBE2D4|YWHAZ*
Cell Cycle	17	*AURKA|BUB1|CCNB1|CCNE1|CDK1|CDKN2B|CKS1B|E2F1|FBXO5|HSP90AA1|MAD2L1|MCM2|MCM4|MCM6|NEK2|OIP5|SMC4*
Pathways in cancer	15	*CCNE1|CDC42|CDKN2B|CKS1B|CTBP1|CYCS|E2F1|HSP90AA1|MSH2|PTK2|RAC1|RAF1|RASSF1|RELA|TCEB2*
Developmental Biology	15	*CDC42|CDK1|CREB1|HSP90AA1|HSPA8|KIF4A|MED20|MYH10|MYH14|NRTN|PTK2|RAC1|RAF1|ROCK1|SPTAN1*
Metabolism of proteins	12	*CTSD|DDIT3|DNAJB9|EEF1A1|EEF1D|EEF1G|EIF5A|HSPD1|RPL10|RPL13A|RPL14|SHC1*
**Arsenic Interacting Common E2- and NRF1-Target Genes**		
**KEGG Pathway**	**Number of Genes**	**Annotated Genes**
Metabolism	33	*ADCY9|ALDOA|CYCS|ENO1|ERCC2|ETFB|ETFDH|FASN|GCLC|GSR|GSS|GSTM3|HSD17B4|HSP90AA1|INPP5A|IP6K2|LDHA|MMADHC|NUBP1|P4HB|PAICS|PLCG1|PLD1|POM121|PPAT|PRKAR2A|SDHA|SGMS1|SLC44A2|STX1A|SUCLA2|TPMT|UBA52*
Immune System	30	*ADCY9|ARPC2|CASP2|CDC42|CDK1|CREB1|CTSD|FZR1|HRAS|HSP90AA1|IP6K2|JAK2|KIF4A|MAPK7|PCBP2|PELI1|PLCG1|PLD1|POM121|PRKAR2A|PRKDC|PTK2|PVR|RAF1|SOS1|SRC|TUBB4B|UBA52|UBE2D4|XRCC5*
Disease	27	*ADCY9|ALDOA|CDC42|CDK1|CDKN2B|CREB1|CTBP1|ENO1|ERCC2|FASN|HDAC4|HRAS|HSP90AA1|MMADHC|NPM1|P4HB|PLCG1|POM121|PRKAR2A|RAF1|RPL36|SMARCA4|SOS1|SRC|STX1A|UBA52|XRCC5*
Signal Transduction	24	*ADCY9|CASP2|CDC42|CDK1|CDKN2B|CREB1|CTBP1|E2F1|HDAC4|HRAS|HSP90AA1|JAK2|MAPK7|P4HB|PLCG1|PRKAR2A|PTCH1|PTK2|RAF1|ROCK1|SMARCA4|SOS1|SRC|UBA52*
Developmental Biology	22	*ABLIM2|ARPC2|CDC42|CDK1|CREB1|HRAS|HSP90AA1|HSPA8|KIF4A|MED18|MYH10|MYH14|NRTN|PLCG1|PTK2|RAF1|ROCK1|SCN3B|SIAH2|SOS1|SRC|TUBB4B*
Metabolic pathways	20	*ALDOA|COX6A2|DNMT1|ENO1|FASN|FUT8|GCLC|GSS|HSD17B4|INPP5A|LAP3|LDHA|PAICS|PLCG1|PLD1|PPAT|SDHA|SGMS1|SUCLA2|SUFU*
Cell Cycle	19	*ATM|AURKA|CCNB1|CCNE1|CDK1|CDKN2B|CDKN2D|E2F1|FZR1|HSP90AA1|MAD2L1|NPM1|PCNA|POM121|SET|SPDL1|TUBB|TUBB4B|UBA52*
Gene Expression	19	*CDKN2B|CNOT1|DNMT1|EEF1G|ERCC2|EZH2|HNRNPA1|HSPA8|LSM2|PCBP2|PTBP1|RNPS1|RPL36|RUNX2|SET|UBA52|UPF1|VARS2|ZNF610*
Pathways in cancer	17	*CCNE1|CDC42|CDKN2B|CTBP1|CYCS|E2F1|HRAS|HSP90AA1|MSH2|PLCG1|PLD1|PTCH1|PTK2|RAF1|RASSF1|SOS1|SUFU*
Cellular responses to stress	16	*ATM|CCNE1|CDKN2B|CDKN2D|CYCS|E2F1|EHMT1|EZH2|FZR1|GSR|HSP90AA1|HSPA8|MAPK7|P4HB|PRDX5|UBA52*
**Manganese Interacting Common E2- and NRF1-Target Genes**		
**KEGG Pathway**	**Number of Genes**	**Annotated Genes**
Cellular responses to stress	6	*ATM|CYCS|GSR|HSPA8|P4HB|RELA*
Developmental Biology	5	*CREB1|HSPA8|SCN3B|SPTAN1|SRC*
Tuberculosis	4	*CREB1|CYCS|RELA|SRC*
p53 signaling pathway	3	*ATM|CYCS|PPM1D*
Small cell lung cancer	3	*CKS2|CYCS|RELA*
Apoptosis	3	*ATM|CYCS|RELA*
Cell cycle	3	*ATM|BUB1|MCM4*

**Table 5:** Interaction of estrogenic endocrine disrupting chemicals modified genes with estrogen signaling and NRF1 network genes in the individual neurodegenerative disease.

Endocrine Disrupting Chemical (EDC)	Individual EDC Responsive Modified Genes Common to Both NRF1 and E2 Target. * Indicates E2 Responsive
**Alzheimer’s Disease (AD)**	
17 β-estradiol	6 genes: *APBB2|DPYSL2|EIF2S1|ENO1|MAPT|PAXIP1*
Ethinyl Estradiol	6 genes: APBB2 *|EIF2S1 *|ENO1 *|IDE|MAPT *|PAXIP1 *
Bisphenol A	8 genes: APBB2 *|DPYSL2 *|EIF2S1 *|ENO1 *|IDE|MAPT *|PAXIP1 *|SLC30A4
Dibutyl Phthalate	4 genes: DPYSL2 *|EIF2S1 *|ENO1 *|IDE
Diethylhexyl Phthalate	2 genes: DPYSL2 *|MAPT *
Cadmium	2 genes: ENO1 *|SLC30A4
Arsenic	2 genes: ENO1 *|MAPT
Manganese	1 gene: ENO1 *
**Parkinson’s Disease (PD)**	
17 β-estradiol	8 genes: *HSPA9|MAPT|RPL14*
Ethinyl Estradiol	3 genes: HSPA9 *|MAPT *|PINK1
Bisphenol A	8 genes: GAK|HSPA9 *|MAPT *|PARK2|PARK7|PINK1|RPL14 *|VPS35
Dibutyl Phthalate	4 genes: HSPA9 *|PARK2|PARK7|RPL14 *
Diethylhexyl Phthalate	3 genes: HSPA9 *|MAPT *|PARK2
Cadmium	3 genes: PARK2|PINK1|RPL14 *
Arsenic	4 genes: GAK|HSPA9 *|MAPT *|PARK2
Manganese	2 genes: *PARK2|PARK7*
**Huntington’s Disease (HD)**	
17 β-estradiol	2 genes: *AIFM1|P6K2*
Ethinyl Estradiol	1 gene: AIFM1 *
Bisphenol A	2 genes: AIFM1 *|IP6K2 *
Dibutyl Phthalate	1 gene: IP6K2 *
Cadmium	1 gene: AIFM1 *
Arsenic	1 gene: IP6K2 *
**Amyotrophic Lateral Sclerosis (ALS)**	
17 β-estradiol	1 gene: *GSR|CHMP2B*
Ethinyl Estradiol	1 gene: GSR *
Bisphenol A	2 genes: GSR *|CHMP2B *|UNC13A
Dibutyl Phthalate	1 gene: GSR *
Diethylhexyl Phthalate	1 gene: GSR *
Cadmium	1 gene: GSR *
Arsenic	1 gene: GSR *
Manganese	1 gene: *GSR*
**Autism Spectrum Disorder (ASD)**	
17 β-estradiol	3 genes: *CIRBP|PCDH9|GTF2I*
Ethinyl Estradiol	2 genes: CIRBP *|GTF2I *
Bisphenol A	3 genes: CIRBP *|GTF2I *|PCDH9 *
Polychlorinated Biphenyls	1 gene: CIRBP *
Cadmium	1 gene: CIRBP *
Arsenic	1 gene: PCDH9 *
**Brain Neoplasms**	
17 β-estradiol	3 genes: *PCNA|PTCH1|RELA*
Ethinyl Estradiol	2 genes: PCNA *|PTCH1 *
Bisphenol A	4 genes: EML4|PCNA *|PTCH1 *|RELA *
Dibutyl Phthalate	2 genes: PCNA *|PTCH1 *
Polychlorinated Biphenyls	1 gene: *PCNA*
Cadmium	1 gene: RELA *
Arsenic	2 genes: PCNA *|PTCH1 *
Manganese	1 gene: RELA *

## Supplementary Materials

Supplementary materials can be found at www.mdpi.com/1422-0067/17/12/2086/s1.
