# Supplementary material for: Estrogenic Endocrine Disrupting Chemicals Influencing NRF1 Regulated Gene Networks in the Development of Complex Human Brain Diseases
Source: Int J Mol Sci. 2016 Dec 13;17(12):2086. doi: 10.3390/ijms17122086 (PMC5187886; doi:10.3390/ijms17122086)
Supplement: Supplementary file 1 [file ijms-17-02086-s001.zip › ijms-160503-Supplementary Materials/ijms-160503 suppl.pdf]

Supplementary Materials: Estrogenic Endocrine Disrupting Chemicals Influencing NRF1 Regulated Gene Networks in the Development of Complex Human Brain Diseases

Mark Preciados, Changwon Yoo and Deodutta Roy

Table S1. 17-β Estradiol interacting genes which are also NRF1 target genes.

Table S2. NRF-1 target genes associated with estrogen and estrogenic endocrine disruptors—BPA, PCB, phthalate, As, Cd, and Mn.

<table>
<tr><th>Estrogenic Endocrine Disruptor</th><th>Number of Genes</th><th>NRF1-Target Genes</th></tr>
<tr><td>17-β Estradiol</td><td>724</td><td>ABCA3|ABCD3|ABI1|ABLIM2|ACADM|ACYP2|ADCY9|ADI1|ADIPOR2|ADM5|AGPAT5|AIFM1|AKAP8L|AKIP1|AKIRIN2|AKR7A2|
AKT2|AKTIP|ALAS1|ALDOA|AMACR|ANAPC1|ANAPC11|ANAPC16|ANKRD37|ANLN|AP1S1|APBB2|APBB3|APITD1|ARAF|ARF4|
ARHGDIA|ARHGEF39|ARL3|ARPC2|ARPC5|ASH1L|ASTN2|ATF5|ATF7IP|ATM|ATP11A|ATP5O|AUH|AURKA|AURKB|B3GNTL1|
BAG4|BASP1|BCAT1|BCAT2|BCL6B|BCL7A|BOP1|BRD2|BRK1|BSG|BTBD3|BTBD7|BUB1|BYSL|C16ORF45|C2ORF76|C4ORF46|
C4ORF48|CACYBP|CANT1|CASK|CASP2|CBFB|CBR1|CBX1|CBX2|CCDC86|CCNB1|CCNE1|CCNG2|CCNI|CCNT2|CCT3|CCT6A|
CCT8|CD27|CD99L2|CDC34|CDC42|CDC7|CDCA2|CDK1|CDK10|CDK2AP2|CDKN2B|CDKN2D|CDT1|CDV3|CENPN|CEP170|
CEP85|CHAC2|CHMP2A|CHMP2B|CHMP5|CHORDC1|CIRBP|CISD1|CKLF|CKS1B|CKS2|CLASP2|CLEC11A|CLIC4|CMC1|CNBP|
CNKSR3|CNOT1|CNST|COMMD8|COPB2|COX5B|COX6A2|COX6C|CPNE1|CREB1|CREM|CRK|CRKL|CRMP1|CSK|CSNK1A1|
CTBP1|CTDSPL2|CTNNA2|CTPS1|CTSD|CYB5A|CYB5R4|CYCS|DAAM1|DAD1|DAP|DCAF8|DDAH1|DDIT3|DDX10|DDX27|
DDX3X|DEDD2|DENND5A|DESI1|DGKH|DHX9|DKC1|DLGAP4|DLL3|DLX4|DNAAF1|DNAH14|DNAJB12|DNAJB2|DNAJB6|
DNAJB9|DNAJC3|DNAJC5|DNMT1|DOHH|DONSON|DPY30|DPYSL2|DSN1|DST|DTYMK|DYNLL1|E2F1|E2F6|EBAG9|EEF1A1|
EEF1D|EEF1G|EHD1|EHMT1|EIF1|EIF2S1|EIF2S2|EIF3K|EIF3L|EIF4E|EIF4G1|EIF5A|ELOVL4|ENO1|EPHX1|EPS15L1|EPS8|ERCC2|
ERCC6|ERCC6L|ERICH1|ERRFI1|ESRP2|ESRRA|ETFB|ETFDH|ETV5|EXOSC2|EXT2|EZH1|EZH2|FADD|FAM102A|FAM118B|
FAM151B|FAM161A|FAM189A1|FAM219A|FAM46C|FAM47E|FAM49B|FARSB|FASN|FAU|FBL|FBXO31|FBXO46|FBXO5|FBXW5|
FCHO2|FDPS|FIGN|FIGNL1|FIGNL2|FKBPL|FSTL3|FUT8|FXYD3|FYTTD1|FZD4|FZR1|G3BP2|GAA|GABRB1|GATAD2A|GCLC|
GEMIN4|GFOD2|GFPT1|GLB1|GLE1|GNG5|GOLGA2|GPBP1L1|GPD1L|GPR37|GPSM2|GRPEL2|GSR|GSS|GSTK1|GSTM3|GTF2E2|
GTF2I|HARS|HAUS3|HBP1|HDAC2|HDAC3|HDAC4|HDLBP|HEATR3|HIRIP3|HMMR|HNRNPA1|HNRNPH1|HNRNPU|HOOK3|
HOXC6|HRAS|HSD17B4|HSDL2|HSF1|HSF2|HSF4|HSP90AA1|HSPA14|HSPA4L|HSPA8|HSPA9|HSPD1|HSPE1|HUNK|HYLS1|
IAH1|IFIT5|IGFBP3|IKZF2|IL17RD|ILVBL|IMMP2L|INPP5A|INPP5K|INTS7|IP6K2|IPO9|IRS1|ITGA1|ITGAE|JAK2|JMY|KANSL1L|
KIAA0100|KIF18A|KIF4A|KIFC1|KLF13|KLHL11|KLHL22|KRCC1|KRR1|KRTCAP2|L3MBTL1|L3MBTL3|LAP3|LDHA|LETM1|LFNG|
LIN37|LIPA|LPCAT3|LRIF1|LRRC27|LRRC58|LSM2|LSR|LSS|LYAR|MACF1|MAD2L1|MAGED2|MAGED4|MAGI1|MANEA|
MAP2K7|MAP4K3|MAPK7|MAPT|MARS2|MBD1|MBD4|MCM2|MCM4|MCM6|MECP2|MECR|MED11|MED18|MED20|MED4|
MEF2A|MEX3B|MGEA5|MGST3|MKNK1|MKNK2|MKS1|MLH3|MLLT3|MMADHC|MMP17|MOK|MPC2|MPP6|MRPL12|MRPL49|
MRPS18B|MSH2|MST1|MTHFD1|MYBBP1A|MYCBP2|MYH10|MYH14|MYO1D|NAA38|NAMPT|NAP1L4|NARF|NBEA|NBPF3|
NDFIP2|NDRG3|NDUFA3|NDUFB4|NEFH|NEGR1|NEIL2|NEIL3|NEK2|NEK4|NF1|NGLY1|NMRK1|NOP2|NPM1|NR1D1|NRTN|
NUBP1|NUP107|NUP50|NUSAP1|NXT1|OAZ2|ODC1|OIP5|ORC6|OS9|OSBPL6|P4HB|PAICS|PAPSS1|PAQR8|PARP3|PAXIP1|
PCBP2|PCDH9|PCNA|PDCD4|PDCD6|PDHB|PDIA4|PDRG1|PEG10|PELI1|PELP1|PER1|PFDN1|PFKFB4|PFN1|PGAM5|PGAP1|</td></tr>
</table>

<table>
<tr><td></td><td></td><td>*PGRMC2|PHC3|PHF12|PIGA|PIP5K1B|PITX2|PKD1|PLA2G12A|PLCG1|PLD1|PLK4|PLS1|PMS2|PNISR|PNMAL1|POLA1|POLE4|POLG2|POLR1A|POLR2A|POLRMT|POM121|POMT1|PPA1|PPAN|PPAT|PPDPF|PPIA|PPIF|PPM1D|PPM1F|PPM1K|PPP1R12A|PPP4R4|PPP6R1|PPTC7|PRAME|PRC1|PRDM4|PRDX5|PRKAR2A|PRKDC|PRMT1|PRRC2C|PRRT2|PSAP|PSENEN|PSPC1|PSTK|PTBP1|PTCH1|PTK2|PTMA|PTPRS|PUS7L|PVR|PXDN|PYGL|RAB1B|RAB32|RAB4A|RABL6|RAC1|RAD50|RAE1|RAF1|RAN|RANBP3|RASSF1|RBBP4|RBBP7|RBBP8|RBKS|RBL2|RBM14|RBM7|RBMXL1|RBX1|RELA|RENBP|RFXANK|RGS19|RGS20|RHD|RHOA|RIPK2|RNF139|RNF14|RNF145|RNF149|RNF19B|RNF32|RNPS1|ROCK1|RPL10|RPL13A|RPL14|RPL36|RPS13|RPS16|RPS21|RPS29|RPS6KA5|RPS6KA6|RPS6KB1|RPS9|RPUSD1|RRP9|RSF1|RSL24D1|RSU1|RTN3|RUNX2|S100A13|SACS|SAMHD1|SAP18|SARS|SBF1|SCAF11|SCAMP4|SCAND1|SCARB2|SCN3B|SCOC|SDCBP|SDCCAG8|SDF4|SDHA|SDHAF4|SEC14L1|SEC22C|SEC61G|SECTM1|SELT|SERTAD1|SESN1|SET|SFMBT2|SFR1|SFXN2|SGCB|SGMS1|SH3BGR|SH3BP5|SHBG|SHC1|SHC4|SHMT2|SIAH2|SIN3B|SIRT5|SLC16A1|SLC22A5|SLC25A10|SLC37A4|SLC39A14|SLC39A6|SLC3A2|SLC44A2|SMAP1|SMARCA4|SMC4|SMIM14|SMYD3|SNRK|SNW1|SNX7|SOCS1|SOS1|SPC24|SPDL1|SPOCD1|SPRY4|SPTAN1|SRC|SRFBP1|SRRM1|SRRM2|SRSF1|SSH2|ST5|STARD13|STARD7|STK38|STPG2|STRN3|STUB1|STX1A|STX3|SUCLA2|SUFU|SUV39H2|SYMPK|TADA2B|TAF12|TAF5|TANGO2|TARS|TBC1D24|TBC1D8|TCAIM|TCEB2|TCF19|TCF25|TCTEX1D2|TGDS|TGIF1|THBS2|THRA|TIA1|TIMELESS|TIPARP|TMED3|TMEM106C|TMEM116|TMEM120B|TMEM139|TMEM14A|TMEM14C|TMEM170B|TMEM38B|TMEM43|TMX1|TNKS2|TNPO2|TOP1|TP53BP2|TPM1|TPMT|TPR|TPX2|TRADD|TRAM1|TRIO|TRUB1|TSPAN12|TSPAN3|TSPYL2|TTC25|TTLL12|TUBB|TUBB4B|TUBD1|TWF2|U2AF1|UBA52|UBE2D4|UBE2T|UNG|UNKL|UPF1|UPF2|USP12|VAPB|VARS2|VASH1|VMA21|VPS8|WDHD1|WDR4|WDR43|WDR5|WNT5A|XPA|XPO5|XRCC2|XRCC5|XRCC6|YWHAZ|ZBED8|ZBTB43|ZBTB8OS|ZFAND2A|ZNF281|ZNF428|ZNF610|ZNF652|ZNF658|ZNF711|ZNF750|ZNF84|ZP3|ZWINT|ZXDC*</td></tr>
<tr><td>Ethinyl estradiol</td><td>800</td><td>*A1BG|AATF|ABCB10|ABCF1|ABCF3|ABHD14A|ABI1|ACAD9|ACADM|ACAT2|ACOT13|ACP1|ACTB|ACTN4|ACTR1A|ACTR8|ACYP2|ADCK5|ADCY9|ADIPOR2|AGPAT5|AIFM1|AKAP8|AKT2|AKTIP|ALAS1|ALDOA|AMZ2|ANAPC1|ANAPC11|ANAPC7|ANGEL2|ANLN|ANXA5|AP1M1|AP1S1|AP2M1|AP3B1|APBA3|APBB2|APBB3|APEX2|APRT|ARAF|ARF1|ARF4|ARHGEF18|ARL2BP|ARL3|ARMCX2|ARPC1A|ARPC2|ARPC5|ASB8|ATAD1|ATAD3A|ATAD3B|ATF5|ATG7|ATP5O|ATP6V0D1|ATP6V0E2|ATP6V1D|ATRN|AURKB|AVEN|B3GALNT2|B3GNTL1|B9D1|BBS2|BCAT1|BCCIP|BCKDHA|BCL6B|BCL7B|BCLAF1|BNIP3|BOP1|BSG|BUB1|BYSL|C20ORF24|CACYBP|CANT1|CASC5|CASP2|CBFB|CBR1|CBX1|CBX7|CBY1|CCDC43|CCNB1|CCNE1|CCNF|CCNG2|CCS|CCT3|CCT5|CCT6A|CCT8|CD320|CD47|CD59|CD99L2|CDC42|CDC42SE2|CDC7|CDK1|CDK2AP2|CDT1|CDV3|CDYL|CENPI|CFDP1|CHMP2A|CHMP3|CHORDC1|CHST12|CHTF18|CIAO1|CIB1|CIRBP|CIZ1|CKLF|CKS1B|CKS2|CLEC11A|CLK3|CLOCK|CLP1|CMAS|CNOT4|COG6|COPB2|COPG1|COQ10B|COQ3|COX5B|COX6C|CPT1C|CREB1|CRK|CRY1|CSE1L|CSK|CSNK1A1|CSTF1|CTNNA2|CTNNBIP1|CTPS1|CTSD|CUTC|CYB5A|CYCS|CYTH2|DAP|DAP3|DAZAP1|DBI|DCAF8|DDAH1|DDIT3|DDX27|DDX39B|DDX41|DDX42|DDX56|DESI1|DHDDS|DHODH|DHX15|DIRC2|DLD|DLGAP4|DNAJA3|DNAJB6|DNAJC3|DNM1L|DNMT1|DOLPP1|DPF2|DST|DSTN|DTNBP1|DUSP14|DUSP22|DYNC1H1|DYNC1LI1|DYNLL1|DYNLRB2|E2F1|E2F6|EED|EEF1A1|EEF1G|EGLN2|EHD1|EI24|EIF1|EIF2S1|EIF2S2|EIF3E|EIF3K|EIF3L|EIF4B|EIF4E|EIF4E2|EIF5A|ELMOD3|ELOF1|ELP5|EMC6|ENO1|EPHX1|EPN3|ERCC2|ERGIC3|ERI3|ERLIN1|ERP29|ERRFI1|ETFDH|ETV5|EVC|EXOG|EXOSC1|EXOSC10|EXOSC5|EXT2|EZH1|EZH2|FADD|FAM102A|FAM120B|FAM122B|FANCA|FARSB|FASN|FAU|FBXL8|FBXO9|FBXW5|FDPS|FGFR1OP2|FKBPL|FKRP|FLYWCH2|FNBP4|FOPNL|FSD1|FUNDC2|FXR2|FXYD3|FZR1|GABPB1|GCLC|GDPGP1|GEMIN2|GIGYF2|GIT2|GLB1|GLRX3|GMEB2|GNAO1|GNPAT|GNPDA2|GOLGA1|GOLGA2|GOLGA4|GPAA1|GPSM2|GRHPR|GRPEL2|GSR|GSS|GSTM3|GSTZ1|GTF2A1|GTF2E2|GTF2F1|GTF2I|GTSE1|HARS|HBP1|HBS1L|HCRT|HDAC2|HDAC3|HDHD2|HDHD3|HDLBP|HERC4|HGS|HINT1|HIRIP3|HMGN1|HMHA1|HNRNPA1|HNRNPH1|HNRNPM|HNRNPU|HOXC6|HRAS|HSD17B4|HSF1|HSP90AA1|HSPA14|HSPA4L|HSPA8|HSPA9|HSPD1|HSPE1|HUNK|ICMT|IDE|IFT172|IFT27|IGFBP3|IKBKAP|IL4I1|ILKAP|INPP5A|INSIG2|INTS6|IPO11|IRF3|ISOC1|ITGA1|KARS|KBTBD2|KCTD9|KDELR2|KIF5B|KLF13|KLHL11|KRTCAP2|L3MBTL3|LARP7|LDHA|LENG1|LETM1|LIG3|LIN37|LIPA|LONP1|LRRC27|LSM4|LSS|LYAR|LYPLA1|LYPLAL1|MACF1|MAD2L1|MAFG|MAGED1|MAGED2|MAP1LC3B|MAP2|*</td></tr>
</table>

<table>
<tr><td></td><td></td><td>MAP2K7|MAPK7|MAPT|MAST2|MBD1|MBD3|MBTD1|MCM2|MCM4|MCM6|MEA1|MECR|MED11|MED25|MED28|MED4|MESP2|MGAT1|MGEA5|MGST3|MIDN|MIP|MKNK2|MLX|MMRN2|MOK|MORF4L1|MORF4L2|MPDU1|MPG|MPHOSPH6|MPP6|MRPL12|MRPL17|MRPL24|MRPL32|MRPL36|MRPL45|MRPL49|MRPL54|MRPS12|MRPS18B|MRPS25|MRPS6|MSH2|MSRA|MTCH2|MTG1|MTHFD1|MTHFSD|MVD|MXI1|MYCBP|MYCBP2|MYH10|N6AMT1|NAA10|NAA16|NAA38|NADSYN1|NAE1|NAGK|NAMPT|NAP1L4|NAPA|NAPB|NAPG|NARFL|NDST2|NDUFA8|NDUFS8|NEFH|NEIL3|NEK4|NEK8|NEUROD1|NF2|NFX1|NOL7|NOMO1|NONO|NPM1|NPRL3|NR1D1|NR1D2|NR2C1|NRBP1|NRTN|NSUN2|NT5C3|NUDT7|NUP35|NUP50|NUSAP1|NXT1|OCIAD1|ODC1|OPA1|ORAOV1|ORC6|OS9|OSTF1|OTUD5|P4HB|PAIP2|PAPSS1|PARD6A|PATL1|PAXIP1|PCBP2|PCGF6|PCIF1|PCNA|PCNT|PDCD4|PDCD5|PDCD6|PDCD6IP|PDE8A|PDHB|PDIA4|PDXDC1|PEG10|PER1|PFDN1|PFN1|PGAM5|PGRMC2|PGS1|PHF23|PI4K2B|PIGA|PIN4|PINK1|PKD1|PKIB|PLEKHA3|PLEKHB2|PLK4|PLOD3|PLS1|PNISR|POLD1|POLE4|POLG2|POLR1A|POLR1C|POLR3K|POLRMT|POMT1|POP1|POP4|POP7|POT1|POU2F1|PPAN|PPAT|PPCS|PPIF|PPP1R12A|PPP2CA|PPP2R1A|PPP2R5C|PPP5C|PPP6C|PPP6R1|PQLC2|PRC1|PRDM4|PRDX3|PREB|PRMT1|PRPF3|PRPF4B|PRPSAP1|PSAP|PSENEN|PSMA2|PSMA4|PSMC4|PSPC1|PTCH1|PTK2|PTMA|PTPN2|PTPN23|PTPRS|PTS|PVR|PXDN|PYGL|QPCTL|RAB14|RAB18|RAB1A|RAB4A|RAB6A|RAB8A|RABEP1|RABGGTB|RABIF|RAE1|RALBP1|RALGDS|RAN|RANBP1|RAP1GDS1|RASSF1|RBBP4|RBBP7|RBBP8|RBKS|RBM12|RBM14|RBM17|RBX1|RCBTB2|RDM1|REEP3|REEP6|RER1|RFXANK|RGS19|RHEBL1|RHOA|RNF130|RNF19A|RNF8|RNH1|RNPS1|RPA1|RPA2|RPL19|RPL36|RPL5|RPL8|RPP14|RPP30|RPS12|RPS16|RPS6KA6|RPS9|RRP9|RSF1|RSG1|RSL1D1|RSPH3|RSU1|RTCA|RTCB|S100A13|SAMHD1|SAP18|SARS|SBDS|SBF1|SCAMP1|SCAND1|SCARB2|SCOC|SDCBP|SDHB|SDHD|SEC61G|SEPT2|SERTAD1|SESN1|SET|SETD4|SGMS1|SH3BP5|SHBG|SHMT1|SIAH2|SIN3B|SIRT5|SIVA1|SLC16A1|SLC22A5|SLC25A10|SLC25A3|SLC27A3|SLC30A5|SLC35A4|SLC35E3|SLC37A4|SLC39A6|SLC3A2|SLK|SMAD4|SMARCA4|SMARCB1|SMARCC1|SMG6|SMYD4|SNAP29|SNRK|SNRNP25|SNX1|SNX4|SOCS1|SPAG6|SPC24|SPDL1|SPECC1L|SPG7|SRD5A1|SRP54|SRP68|SRPRB|SRSF1|SRSF11|SRSF4|SRSF9|SS18L2|SSB|ST5|STAG1|STARD10|STARD7|STK25|STK38|STRN3|STUB1|STX17|STX2|STX3|SUCO|SUPT3H|SUV39H2|SZRD1|TAF12|TAF5|TAF6|TARS|TAX1BP3|TBCB|TBK1|TCEB2|TCF19|TCP1|TCTA|TDG|TERF2IP|TFDP1|THOP1|THRA|TIMELESS|TIMM21|TIMM8B|TIPARP|TM9SF2|TMED9|TMEM109|TMEM115|TMEM116|TMEM127|TMEM131|TMEM135|TMEM14C|TMEM206|TMEM39A|TMEM41B|TMEM43|TMEM53|TMEM9|TOMM20|TOMM34|TOP1|TOPORS|TPCN2|TPM1|TRAPPC5|TRIM28|TRIM32|TRIM39|TRIO|TRMT2A|TSEN34|TSPAN14|TSPAN3|TSPYL2|TSSC4|TTC13|TTC25|TUBA1B|TUBB4B|TUBG2|TXLNA|TYMP|UBA2|UBE2D3|UBE2K|UBE2M|UBE2T|UBE4B|UBTD1|UIMC1|UPF1|UQCR11|UQCRB|UQCRC1|UQCRFS1|USP10|USP21|USP39|VAMP2|VARS2|VASH1|VDAC3|VGF|VHL|VPRBP|VPS28|VPS8|WAC|WDHD1|WDR1|WDR19|WDR4|WDR45|WDR5|WDR5B|WDR60|WDR62|WNT5A|WRB|XPO7|XRCC1|XRCC5|YIF1A|YIPF5|YWHAH|YWHAZ|ZC3H14|ZC3H3|ZDHHC12|ZDHHC16|ZFAND2A|ZMAT3|ZP3</td></tr>
<tr><td>Bisphenol-A</td><td>2113</td><td>A1BG|AARSD1|AATF|ABCA3|ABCB10|ABCD3|ABCF1|ABHD14A|ABHD18|ABHD5|ABI1|ABLIM2|ACAD9|ACADM|ACAP3|ACAT2|ACD|ACIN1|ACOT13|ACP1|ACTB|ACTN4|ACTR1A|ACTR8|ACYP2|ADAM10|ADAT3|ADCK3|ADCK5|ADCY9|ADI1|ADIPOR2|AEBP2|AFG3L2|AFTPH|AGPAT1|AGPAT5|AIFM1|AIMP2|AIP|AK3|AKAP17A|AKAP8|AKAP8L|AKIP1|AKIRIN2|AKR7A2|AKT2|AKTIP|ALAS1|ALDH16A1|ALDOA|ALG12|ALG8|ALKBH5|ALYREF|AMACR|AMZ2|ANAPC1|ANAPC11|ANAPC13|ANAPC15|ANAPC16|ANAPC2|ANAPC7|ANGEL2|ANGPTL6|ANKRD13D|ANKRD26|ANKRD31|ANKRD37|ANLN|ANXA5|AP1M1|AP1S1|AP2M1|AP3B1|AP4S1|APBA3|APBB2|APC|APC2|APEX2|APLF|APOA1BP|APOLD1|APRT|AQP11|ARAF|ARF1|ARF4|ARF6|ARFGAP3|ARHGAP44|ARHGDIA|ARHGEF10|ARHGEF18|ARHGEF39|ARL10|ARL13B|ARL16|ARL2BP|ARL3|ARL5A|ARMC2|ARMC6|ARMC7|ARMCX2|ARPC1A|ARPC2|ARPC5|ARRB2|ARSB|ARV1|ASB3|ASB8|ASCC1|ASH1L|ASNSD1|ASTN2|ASUN|ATAD1|ATAD3A|ATAT1|ATF5|ATF7IP|ATG10|ATG2A|ATG5|ATG7|ATM|ATOH7|ATP11A|ATP5G2|ATP5I|ATP5O|ATP6V0D1|ATP6V0E2|ATP6V1C1|ATP6V1D|ATP6V1F|ATP6V1G1|ATPAF2|ATRN|ATXN2L|ATXN7L3B|AURKA|AURKB|AVEN|B3GALNT2|B3GALT6|B3GNT4|B3GNTL1|B4GALT5|B9D1|B9D2|BAG1|BAG4|BASP1|BBS9|BCAN|BCAR1|BCAT1|BCAT2|BCCIP|BCKDHA|BCL10|BCL2L12|BCL6B|BCL7A|BCL7B|BCLAF1|BLOC1S2|BLOC1S4|BNIP3|BORCS7|BPNT1|BRD2|BRK1|BROX|BRSK1|BSG|BTBD7|BTRC|</td></tr>
</table>

<table>
<tr>
<td></td>
<td>BUB1|BUB3|BVES|BYSL|C10ORF137|C10ORF88|C11ORF71|C12ORF45|C12ORF65|C14ORF119|C14ORF2|C14ORF28|C14ORF80|
C15ORF40|C16ORF45|C16ORF70|C16ORF87|C17ORF97|C18ORF54|C19ORF47|C19ORF52|C19ORF67|C19ORF81|C1GALT1C1|
C1ORF109|C1ORF131|C1ORF228|C1ORF27|C1ORF35|C1ORF50|C1ORF53|C1QBP|C20ORF194|C20ORF24|C21ORF2|C21ORF58|
C22ORF23|C22ORF29|C2CD3|C2ORF73|C2ORF74|C3ORF67|C4ORF46|C4ORF48|C5ORF24|C6ORF136|C8ORF37|C8ORF48|C9ORF40|
C9ORF69|CAB39|CACUL1|CACYBP|CANT1|CAPZA2|CARNMT1|CASK|CASKIN2|CASP2|CASZ1|CATSPERD|CBFB|CBLL1|CBR1|
CBR4|CBX1|CBX2|CBX5|CBX7|CBY1|CBY3|CC2D1A|CC2D1B|CCDC11|CCDC114|CCDC12|CCDC120|CCDC127|CCDC150|CCDC167|
CCDC171|CCDC28B|CCDC43|CCDC47|CCDC86|CCDC87|CCDC89|CCDC92|CCDC93|CCDC96|CCDC97|CCHCR1|CCNB1|CCNE1|
CCNF|CCNG2|CCNI|CCNJL|CCNT2|CCS|CCT3|CCT5|CCT6A|CCT8|CD27|CD274|CD320|CD47|CD59|CD99L2|CDAN1|CDC34|
CDC42|CDC42BPB|CDC42EP4|CDC42SE2|CDC7|CDCA2|CDK1|CDK10|CDK16|CDK19|CDK2AP2|CDKAL1|CDKL3|CDKN2B|
CDKN2D|CDNF|CDPF1|CDS2|CDT1|CDV3|CDYL|CENPI|CENPN|CEP104|CEP170|CEP19|CEP192|CEP250|CEP57L1|CEP63|CEP85|
CES4A|CFAP206|CFDP1|CHAC2|CHCHD4|CHD2|CHD3|CHKB|CHMP1A|CHMP2A|CHMP2B|CHMP3|CHMP5|CHMP7|CHORDC1|
CHST12|CHTF18|CHTF8|CHTOP|CIAO1|CIB1|CIC|CIRBP|CIRH1A|CISD1|CISD2|CIZ1|CKLF|CKS1B|CKS2|CLASP1|CLASP2|CLCN5|
CLEC11A|CLIC4|CLK3|CLN5|CLN8|CLOCK|CLP1|CLPTM1L|CLPX|CMAS|CMC1|CMC2|CNBP|CNKSR3|CNNM3|CNOT1|CNOT11|
CNOT4|CNP|CNPY4|CNST|CNTLN|CNTNAP1|CNTROB|COG1|COG2|COG6|COMMD8|COPB2|COPG1|COQ10B|COQ3|COX14|
COX15|COX18|COX5B|COX6C|CPSF1|CPSF3L|CPT1C|CREB1|CREM|CRIPT|CRK|CRKL|CRMP1|CRTC1|CRY1|CRYZL1|CSAD|
CSDE1|CSE1L|CSK|CSNK1A1|CSNK1G2|CSTF1|CTBP1|CTDNEP1|CTDSP1|CTDSPL2|CTNNA2|CTNNBIP1|CTPS1|CTSD|CTU1|
CUTC|CXORF38|CXXC1|CYB5A|CYB5R4|CYCS|CYHR1|CYTH1|CYTH2|D2HGDH|DAAM1|DAD1|DALRD3|DAP|DAP3|DAZAP1|
DBI|DCAF6|DCAF8|DCPS|DCUN1D1|DCUN1D2|DDAH1|DDIT3|DDRGK1|DDX27|DDX39A|DDX39B|DDX3X|DDX41|DDX42|
DDX56|DECR2|DENND3|DENND5A|DESI1|DFFB|DGKH|DHDDS|DHODH|DHX15|DHX34|DHX9|DIDO1|DIP2A|DIRC2|DISP2|
DKC1|DLD|DLGAP4|DLL3|DLX4|DMXL2|DNA2|DNAAF1|DNAH2|DNAI1|DNAJA3|DNAJB12|DNAJB2|DNAJB6|DNAJB9|DNAJC25|
DNAJC28|DNAJC3|DNAJC5|DNAL1|DNM1L|DNMT1|DOCK3|DOCK9|DOHH|DOLPP1|DONSON|DPF2|DPY19L2|DPY30|DPYSL2|
DSCR3|DSN1|DST|DSTN|DTNBP1|DTYMK|DUS3L|DUSP11|DUSP14|DUSP18|DYNC1H1|DYNLL1|DYNLRB2|DYNLT1|DYRK1B|
E2F1|E2F6|EBAG9|ECSIT|EDEM2|EED|EEF1A1|EEF1D|EEF1G|EGLN2|EHD1|EHMT1|EI24|EID1|EID2|EID2B|EIF1|EIF1AX|
EIF2AK1|EIF2D|EIF2S1|EIF2S2|EIF3E|EIF3K|EIF3L|EIF4B|EIF4E|EIF4E2|EIF4G1|EIF4H|EIF5A|EIF5B|ELL|ELMOD3|ELOF1|ELOVL4|
ELP2|ELP5|ELP6|EMC10|EMC6|EMD|EML4|ENDOV|ENKUR|ENO1|ENTPD5|ENTPD6|EPHX1|EPN3|EPS8|ERCC2|ERCC6|ERCC6L|
ERGIC3|ERI1|ERI3|ERICH1|ERLIN1|ERMAP|ERP29|ERRFI1|ESCO1|ESRP2|ESRRA|ETFB|ETFDH|ETV5|EVC|EXOC8|EXOG|
EXOSC1|EXOSC10|EXOSC2|EXOSC5|EXT2|EZH1|EZH2|FADD|FAM102A|FAM104A|FAM109A|FAM118A|FAM118B|FAM120A|
FAM120B|FAM122A|FAM122B|FAM133B|FAM134A|FAM134C|FAM151B|FAM161A|FAM175A|FAM189A1|FAM195B|FAM206A|
FAM210B|FAM213A|FAM219A|FAM219B|FAM220A|FAM222B|FAM227A|FAM32A|FAM46C|FAM49B|FAM58A|FAM60A|FAM71E1|
FAM98A|FANCA|FANCL|FARSB|FASN|FAU|FBL|FBXL5|FBXL8|FBXO15|FBXO21|FBXO31|FBXO42|FBXO48|FBXO5|FBXO7|
FBXO9|FBXW5|FCHO2|FDPS|FDX1L|FGFR1OP2|FGGY|FIGN|FIGNL1|FIP1L1|FITM2|FKBP1A|FKBP2|FKRP|FLYWCH1|FMNL1|
FMR1|FNBP4|FOCAD|FOPNL|FOXO4|FPGT|FRMD8|FSD1|FSD1L|FSTL3|FUNDC2|FUT11|FUT8|FXN|FXR2|FXYD3|FZD4|FZR1|
G3BP2|GAA|GABPB1|GABRB1|GABRB2|GAK|GALNT11|GAPVD1|GATAD2A|GCLC|GDPD1|GEMIN2|GEMIN4|GEN1|GFOD2|
GFPT1|GID8|GINM1|GIT1|GIT2|GLB1|GLIPR1L2|GLRX3|GLTPD1|GMCL1|GMEB2|GNAO1|GNB1L|GNG5|GNPAT|GNPDA2|
GNPTAB|GNPTG|GNS|GOLGA1|GOLGA2|GOLGA4|GON4L|GOPC|GPAA1|GPANK1|GPATCH11|GPATCH8|GPBP1L1|GPD1L|
GPR101|GPR180|GPR19|GPR37|GPRIN1|GPSM2|GPSM3|GRHPR|GRIPAP1|GRPEL2|GRSF1|GSG2|GSR|GSS|GSTK1|GSTM3|GSTZ1|
GTF2A1|GTF2E2|GTF2F1|GTF2H4|GTF2I|GTF3C1|GTPBP10|GTPBP8|GTSE1|GUCD1|GXYLT1|GZF1|HARS|HARS2|HAUS3|HAUS6|
HAUS8|HBP1|HBS1L|HCRT|HDAC2|HDAC3|HDAC4|HDHD2|HDHD3|HDLBP|HEATR3|HEATR5B|HERC4|HEXIM1|HEXIM2|HGS|
HINFP|HIRIP3|HLCS|HMGN1|HMMR|HNRNPA1|HNRNPH1|HNRNPM|HNRNPU|HNRNPUL2|HOOK3|HOXC6|HP1BP3|HRAS|
HRSP12|HSD17B4|HSDL1|HSDL2|HSF1|HSF2|HSF4|HSP90AA1|HSPA14|HSPA8|HSPA9|HSPD1|HSPE1|HUNK|HYLS1|IAH1|IBA57|</td>
</tr>
</table>

<table>
<tr>
<td></td>
<td>ICMT|IDE|IFNAR1|IFT122|IFT172|IFT27|IGFBP3|IGFBPL1|IGFLR1|IKBKAP|IKZF2|IL17RD|ILKAP|ILVBL|IMMP2L|IMMT|ING2|
INO80C|INPP4A|INPP5A|INPP5K|INSIG2|INTS10|INTS2|INTS3|INTS5|INTS6|INTS7|IP6K1|IP6K2|IPMK|IPO11|IPO9|IPP|IQCE|
IRAK4|IRF2BPL|IRF3|IRS1|ISOC1|ITFG2|ITGA1|ITGAE|IWS1|JAK2|JMJD4|JMY|JPH4|KANK3|KARS|KATNA1|KCNC3|KCNN1|
KCTD13|KCTD7|KCTD9|KDELR2|KDM3A|KDM4A|KDM5C|KEAP1|KIAA0100|KIAA0513|KIAA0556|KIAA0586|KIAA0895|KIAA2026|
KIF16B|KIF18A|KIF26A|KIF27|KIF2A|KIF3A|KIF4A|KIF5B|KIF6|KIFAP3|KIFC1|KLF13|KLHDC3|KLHL11|KLHL22|KLHL26|KLHL35|
KPNA1|KRCC1|KRI1|KRR1|KRTCAP2|KXD1|L3MBTL1|LAGE3|LAMTOR4|LAPTM4A|LARP7|LDHA|LEMD2|LENG1|LENG8|LEPRE1|
LETM1|LFNG|LGI4|LIG3|LIN37|LIPA|LIPT2|LLGL1|LMF1|LONP1|LPCAT1|LPCAT3|LRBA|LRCH3|LRIF1|LRPAP1|LRRC10B|
LRRC14|LRRC20|LRRC24|LRRC27|LRRC42|LRRC46|LRRC58|LRRC63|LRRIQ3|LSM1|LSM11|LSM12|LSM14B|LSM2|LSM4|LSM7|LSR|
LSS|LUC7L|LYAR|LYPLA1|LYPLAL1|LYRM2|LYSMD3|MACF1|MAD2L1|MAFG|MAGED1|MAGED2|MAGI1|MAGIX|MAN1B1|
MANEA|MAP1LC3B|MAP2|MAP2K7|MAP3K14|MAP4K3|MAP7D2|MAPK12|MAPK7|MAPKAP1|MAPRE3|MAPT|MARS2|MAST2|
MATN1|MBD1|MBD3|MBD6|MCM2|MCM4|MCM6|MCMBP|MCRS1|MEA1|MEAF6|MECP2|MECR|MED11|MED18|MED20|
MED25|MED28|MED4|MED9|MEGF8|MEMO1|MEPCE|MESDC2|MESP1|MESP2|METTL15|METTL22|METTL8|MEX3B|MFSD8|
MFSD9|MGA|MGAT1|MGEA5|MGST3|MIB1|MIDN|MINK1|MIP|MIS18A|MKKS|MKL2|MKNK1|MKNK2|MKRN1|MKS1|MLH3|
MLL2|MLLT3|MLST8|MLX|MMAA|MMADHC|MMP17|MMP25|MOB3A|MOCS2|MOK|MORC3|MORF4L1|MORF4L2|MORN1|
MORN5|MPC2|MPDU1|MPG|MPHOSPH6|MPL|MPP6|MRGBP|MRM1|MRPL10|MRPL12|MRPL14|MRPL17|MRPL24|MRPL32|
MRPL36|MRPL45|MRPL49|MRPL54|MRPS12|MRPS18B|MRPS25|MRPS6|MRS2|MSH2|MSH5|MSL2|MSRA|MST1|MTCH2|MTFMT|
MTG1|MTHFD1|MTURN|MVD|MXI1|MYBBP1A|MYCBP|MYCBP2|MYH10|MYH14|MYH7B|MYO1D|MYO1F|MYO9A|MYPOP|
MZT2B|N4BP1|N6AMT1|N6AMT2|NAA10|NAA16|NAA25|NAA38|NAA40|NAA50|NACAD|NADK|NADSYN1|NAE1|NAGK|
NAMPT|NANP|NAP1L4|NAPA|NAPB|NAPG|NAPSA|NARF|NARFL|NARS2|NAT9|NBEA|NCAN|NCAPD3|NCKIPSD|NCOA5|
NDEL1|NDFIP2|NDNL2|NDRG3|NDST2|NDUFA11|NDUFA12|NDUFA3|NDUFA6|NDUFA8|NDUFAF4|NDUFB10|NDUFB4|
NDUFS6|NDUFV3|NEFH|NEFM|NEGR1|NEIL2|NEIL3|NEK2|NEK4|NEK8|NEUROD1|NF1|NF2|NFU1|NFX1|NFYA|NGLY1|NIPA2|
NKTR|NMRK1|NOB1|NOL7|NOMO1|NONO|NOP2|NOTCH4|NPAT|NPLOC4|NPM1|NPRL3|NR1D1|NR1D2|NR2C1|NR2C2AP|
NRBP1|NRN1|NRTN|NSUN2|NT5C3|NT5DC1|NUBP1|NUDCD2|NUDT15|NUDT16L1|NUDT7|NUP107|NUP35|NUP50|NUP62|
NUSAP1|NXT1|OARD1|OAZ2|OCEL1|OCIAD1|OCLN|ODC1|OIP5|OPA1|ORAOV1|ORC4|ORC6|ORMDL1|OS9|OSBPL6|OSER1|
OSTC|OSTF1|OTUD3|OTUD5|OTUD6B|OXSM|P4HA1|P4HB|PAICS|PAIP2|PAM16|PAPD7|PAPSS1|PAQR4|PAQR8|PARD6A|
PARD6B|PARK2|PARK7|PARN|PARP3|PARP6|PATL1|PATZ1|PAXIP1|PCBP2|PCDH9|PCGF1|PCGF3|PCGF6|PCLO|PCMT1|
PCMTD1|PCMTD2|PCNA|PCSK4|PCYOX1|PDCD2L|PDCD4|PDCD5|PDCD6|PDCD6IP|PDCL3|PDDC1|PDE7A|PDE8A|PDHB|
PDIA4|PDIA5|PDRG1|PDXDC1|PDZD11|PEG10|PEG3|PELI1|PELP1|PER1|PEX5L|PFDN1|PFKFB4|PFN1|PGAM1|PGAM5|PGAP1|
PGLS|PGRMC2|PGS1|PHACTR4|PHC3|PHF12|PHF14|PHF20|PHF23|PHF6|PI4K2A|PI4K2B|PIAS4|PIGA|PIGF|PIGH|PIGP|PIGT|
PIGX|PIKFYVE|PILRB|PINK1|PIP5K1B|PITHD1|PITPNB|PITX2|PJA1|PKD1|PKD2L2|PKIB|PKMYT1|PLA2G12A|PLCG1|PLD1|
PLEKHA3|PLEKHA8|PLEKHB2|PLEKHJ1|PLK4|PLOD3|PLS1|PM20D2|PMS1|PMS2|PNISR|PNMAL1|PNMAL2|POC1A|POFUT2|
POLA1|POLD1|POLG2|POLR1A|POLR1C|POLR2A|POLR2F|POLR2H|POLR2I|POLR3B|POLR3H|POLR3K|POM121|POMT1|POMT2|
POP1|POP4|POP7|POT1|POU2F1|PPA1|PPAN|PPAT|PPCS|PPDPF|PPIA|PPIF|PPM1D|PPM1K|PPME1|PPP1CB|PPP1R10|PPP1R11|
PPP1R12A|PPP1R27|PPP1R7|PPP2R1A|PPP2R3B|PPP2R5C|PPP4R2|PPP4R4|PPP5C|PPP6R1|PPTC7|PQLC2|PRAME|PRC1|PRDX3|
PRDX5|PREB|PRKAR2A|PRKDC|PRMT1|PRPF3|PRPF39|PRPF4B|PRPSAP1|PRR14|PRR29|PRRC1|PRRC2C|PSAP|PSENEN|PSMA2|
PSMA3|PSMA4|PSMC4|PSPC1|PSTK|PSTPIP2|PTBP1|PTCD3|PTCH1|PTGES3|PTGR2|PTK2|PTMA|PTPN2|PTPN20|PTPN23|
PTPRS|PTS|PTTG1IP|PUS7L|PUSL1|PVR|PWP1|PXDN|PYGL|QPCTL|RAB10|RAB14|RAB18|RAB1A|RAB1B|RAB23|RAB32|RAB33B|
RAB35|RAB4A|RAB6A|RAB6B|RAB8A|RAB9B|RABEP1|RABGGTA|RABGGTB|RABIF|RABL6|RAC1|RAD21|RAD23B|RAD50|
RAD54B|RAE1|RAF1|RALBP1|RALGAPA2|RALGDS|RAN|RANBP1|RANBP3|RAP1GDS1|RAPGEF6|RASSF1|RAVER1|RB1CC1|RBAK|
RBBP4|RBBP7|RBBP8|RBFA|RBKS|RBL2|RBM12|RBM14|RBM17|RBM19|RBM4B|RBM7|RBMX2|RBX1|RCAN3|RDM1|REEP3|</td>
</tr>
</table>

<table>
<tr><td></td><td>REEP4|REEP6|RELA|RELL2|RENBP|RER1|RETSAT|REXO2|RFWD2|RFX1|RFX2|RFXANK|RGS17|RGS19|RGS20|RHEBL1|RHOA|
RIOK2|RIPK2|RNF123|RNF139|RNF14|RNF145|RNF149|RNF170|RNF19A|RNF19B|RNF24|RNF32|RNF38|RNF5|RNF8|RNGTT|RNH1|
RNPS1|ROCK1|ROPN1L|RPA1|RPA2|RPL13A|RPL14|RPL19|RPL36|RPL5|RPL7L1|RPL8|RPLP1|RPP14|RPP30|RPP38|RPS12|RPS13|
RPS16|RPS19BP1|RPS21|RPS23|RPS29|RPS5|RPS6KA5|RPS6KA6|RPS6KB1|RPS9|RPUSD1|RPUSD3|RRM2B|RRP1|RRP9|RSBN1|
RSF1|RSG1|RSL1D1|RSL24D1|RSPH3|RSRP1|RSU1|RTCA|RTCB|RTDR1|RTKN2|RTN3|RUNX2|RWDD2B|RXRB|SACS|SAFB|
SAFB2|SAMHD1|SAP18|SARM1|SARS|SARS2|SAYSD1|SBDS|SBF1|SBF2|SCAF11|SCAMP1|SCAMP3|SCAMP4|SCAND1|SCAP|
SCARB2|SCN3B|SCOC|SDCBP|SDCBP2|SDCCAG8|SDF4|SDHA|SDHAF4|SDHB|SDHD|SDR39U1|SEC14L1|SEC24C|SEC24D|
SEC61A2|SEC61G|SECTM1|SEH1L|SELT|SENP2|SENP3|SENP8|SEPT2|SERINC1|SERTAD1|SESN1|SET|SETD4|SF3A2|SF3B1|SF3B2|
SFI1|SFMBT1|SFMBT2|SFR1|SFT2D1|SFXN2|SGCA|SGCB|SGMS1|SGTA|SH3BGR|SH3BP5|SHBG|SHC1|SHC4|SHMT1|SHMT2|
SHPRH|SIAH2|SIKE1|SIL1|SIN3B|SIRT5|SIRT6|SIRT7|SIT1|SIVA1|SKA1|SLC12A6|SLC16A1|SLC16A13|SLC22A5|SLC25A10|
SLC25A3|SLC25A6|SLC27A3|SLC30A4|SLC30A5|SLC35A4|SLC35A5|SLC35B3|SLC35D1|SLC35E3|SLC35G1|SLC36A4|SLC37A4|
SLC39A14|SLC39A6|SLC3A2|SLC44A2|SLC9A3|SLK|SLTM|SLU7|SLX4|SLX4IP|SMAD4|SMAP1|SMAP2|SMARCA4|SMARCB1|
SMARCC1|SMARCD3|SMC4|SMC5|SMG6|SMG9|SMIM14|SMIM4|SMIM7|SMPD4|SMYD3|SMYD4|SNAP23|SNAP29|SNAPC1|
SNRK|SNRNP25|SNRNP70|SNRPD2|SNURF|SNW1|SNX1|SNX14|SNX32|SNX4|SNX7|SNX8|SOCS1|SOCS4|SORCS3|SOS1|SOS2|
SOX12|SPACA5|SPAG6|SPAG8|SPAST|SPATA1|SPATA4|SPATA5L1|SPATS2|SPC24|SPDL1|SPECC1L|SPOCD1|SPOP|SPPL2B|
SPPL3|SPRTN|SPRY4|SPRYD7|SPTAN1|SPTLC1|SPTY2D1|SRC|SRCRB4D|SRD5A1|SRFBP1|SRP54|SRP68|SRPK1|SRPRB|SRR|
SRRM1|SRSF1|SRSF10|SRSF11|SRSF4|SRSF9|SSB|SSH1|SSH2|SSNA1|SSSCA1|ST5|STAG1|STAP2|STARD10|STARD13|STIM2|
STK25|STK38|STPG2|STRADA|STRN3|STRN4|STUB1|STX12|STX17|STX1A|STX2|STX3|STX4|STX7|SUCLA2|SUCO|SUFU|SUMF2|
SUPT3H|SUV39H2|SWI5|SYCE2|SYF2|SYMPK|SYNGR4|SYP|SYPL1|SYS1|SZRD1|TADA2B|TAF12|TAF1B|TAF4|TAF5|TAF6|TAF7|
TANGO2|TAOK2|TAOK3|TARS|TAX1BP3|TBC1D10B|TBC1D20|TBC1D24|TBC1D25|TBC1D5|TBC1D7|TBC1D8|TBCA|TBCB|TBCD|
TBK1|TCAIM|TCEANC|TCEB2|TCF19|TCF25|TCP1|TCTA|TCTEX1D2|TDG|TDP2|TDRD7|TDRP|TECR|TERF2IP|TEX264|TEX30|
TFAP2B|TFB2M|TFDP1|TFG|TGDS|TGIF1|THAP7|THBS2|THNSL1|THOC3|THOP1|THRA|TIA1|TIGAR|TIMELESS|TIMM13|
TIMM21|TIMM8B|TIMM9|TIPARP|TM2D3|TM9SF2|TMBIM6|TMCO6|TMED3|TMED4|TMED5|TMED9|TMEM106B|TMEM106C|
TMEM109|TMEM115|TMEM116|TMEM120B|TMEM127|TMEM131|TMEM135|TMEM139|TMEM141|TMEM143|TMEM145|TMEM14A|
TMEM14C|TMEM161B|TMEM170A|TMEM170B|TMEM175|TMEM184B|TMEM19|TMEM206|TMEM219|TMEM237|TMEM254|
TMEM263|TMEM38B|TMEM39A|TMEM41B|TMEM43|TMEM53|TMEM55B|TMEM63A|TMEM65|TMEM70|TMEM9|TMEM91|TMF1|
TMTC2|TMX1|TMX3|TMX4|TNFSF13|TNKS2|TNPO2|TOLLIP|TOMM20|TOMM20L|TOMM34|TOMM7|TOP1|TOP1MT|TOX2|
TP53BP2|TPBGL|TPCN2|TPGS1|TPM1|TPMT|TPR|TPRA1|TPX2|TRADD|TRAF3IP1|TRAM1|TRAM1L1|TRAPPC1|TRAPPC12|
TRAPPC13|TRAPPC3|TRAPPC5|TRAPPC9|TRIM23|TRIM28|TRIM32|TRIM37|TRIM39|TRIM46|TRIM59|TRIM62|TRIT1|TRMO|
TRMT11|TRMT112|TRMT2A|TRNAU1AP|TRO|TRPC1|TRPM7|TRUB1|TSEN2|TSEN34|TSEN54|TSPAN12|TSPAN3|TSPYL2|TSPYL5|
TSR3|TSSC4|TTBK2|TTC13|TTC25|TTC34|TTC37|TTC7B|TTLL12|TTLL9|TUBA1B|TUBB|TUBB4B|TUBD1|TUBG1|TUBG2|TUBGCP4|
TUSC1|TXLNA|TXNDC11|TXNDC9|TXNL1|TYMP|U2AF1|U2AF1L4|U2AF2|UBA2|UBA52|UBAC2|UBE2D3|UBE2D4|UBE2K|
UBE2M|UBE2N|UBE2T|UBE2V1|UBE4B|UBN2|UBR4|UBTD1|UBXN7|UCK1|UGGT2|UIMC1|UNC13A|UNC45A|UNG|UPF1|UPF2|
UQCC|UQCR10|UQCR11|UQCRB|UQCRC1|UQCRFS1|URGCP|URI1|UROS|USF1|USO1|USP10|USP12|USP21|USP22|USP28|
USP37|USP39|UTP18|UTP20|UTP23|UVSSA|UXS1|VAMP2|VAPA|VAPB|VASH1|VDAC2|VDAC3|VGF|VHL|VKORC1L1|VMA21|
VMAC|VPS13A|VPS13C|VPS26B|VPS28|VPS35|VPS4B|VPS8|VRK3|WAC|WARS2|WDFY2|WDHD1|WDR1|WDR13|WDR19|WDR20|
WDR4|WDR43|WDR44|WDR45|WDR5|WDR5B|WDR60|WDR62|WDR78|WDSUB1|WDTC1|WIPI2|WNK3|WNT5A|WRB|WRNIP1|
WWP1|XPA|XPO5|XPO7|XRCC1|XRCC5|XRCC6|YARS2|YEATS4|YIF1A|YIPF2|YIPF3|YIPF5|YTHDC1|YTHDF1|YWHAH|YWHAZ|
ZBED4|ZBTB17|ZBTB2|ZBTB24|ZBTB4|ZBTB43|ZBTB49|ZBTB8OS|ZC2HC1C|ZC3H14|ZC3H15|ZC3H18|ZC3H3|ZC4H2|ZCCHC7|
ZCWPW1|ZDHHC16|ZFAND2A|ZFAND3|ZFP1|ZFP14|ZFP161|ZFP28|ZFR|ZFYVE16|ZFYVE27|ZGRF1|ZMAT2|ZMAT3|ZMAT5|</td></tr>
</table>

<table>
<tr><td></td><td></td><td>ZMYM1|ZMYM4|ZMYM6|ZMYND12|ZMYND19|ZNF14|ZNF174|ZNF175|ZNF180|ZNF248|ZNF251|ZNF280B|ZNF281|ZNF287|ZNF329|ZNF438|ZNF445|ZNF483|ZNF518B|ZNF524|ZNF565|ZNF583|ZNF652|ZNF667|ZNF692|ZNF706|ZNF711|ZNF740|ZNF761|ZNF770|ZNF784|ZNF800|ZNF845|ZNFX1|ZP3|ZRSR2|ZSCAN18|ZSCAN30|ZWINT|ZXDC|ZYG11B</td></tr>
<tr><td>Dibutyl phthalate</td><td>756</td><td>AATF|ABCB10|ABCF1|ABHD14A|ACAD9|ACADM|ACAP3|ACAT2|ACD|ACOT13|ACP1|ACTB|ACTN4|ADCK5|ADCY9|ADI1|ADIPOR2|AIP|AKAP17A|AKIP1|AKR7A2|ALAS1|ALDH16A1|ALDOA|ALG8|ALKBH4|AMACR|AMZ2|ANAPC1|ANAPC11|ANAPC16|ANKRD37|ANXA5|APC2|APITD1|APLF|APOA1BP|ARF4|ARF6|ARHGDIA|ARHGEF10|ARHGEF18|ARL2BP|ARL5A|ARMC2|ARMC6|ARMCX2|ARPC1A|ARPC2|ARPC5|ARRB2|ARSB|ASCC1|ATAT1|ATF5|ATG10|ATM|ATP5I|ATP5O|ATP6V0E2|ATP6V1F|ATPAF2|ATRN|AURKB|BAG1|BAG4|BASP1|BCAT1|BCAT2|BCKDHB|BCL10|BCLAF1|BLOC1S5|BNIP3|BOP1|BROX|BSG|BTF3|BTRC|BUB3|C1GALT1C1|C1QBP|CAB39|CASC5|CBFB|CBR1|CBR4|CBX5|CBY1|CCDC12|CCDC28B|CCNB1|CCNE1|CCNF|CCNT2|CCT3|CD320|CD47|CD59|CD99L2|CDC7|CDCA2|CDK1|CDK2AP2|CDKN2D|CDPF1|CDT1|CDV3|CENPN|CEP250|CEP57L1|CEP85|CHAC2|CHTF18|CISD1|CISD2|CKS1B|CKS2|CLASP2|CLEC11A|CLIC4|CLN8|CLPX|CMAS|CNBP|CNKSR3|CNP|CNPY4|CNST|CNTLN|CNTROB|COPG1|COQ10B|COQ3|COX15|COX18|COX6A2|CPSF3L|CREB1|CREM|CRIPT|CRTC1|CSNK1A1|CSNK1G2|CTBP1|CTDNEP1|CTNNA2|CTSD|CUTC|CYB5A|CYCS|CYHR1|CYTH2|DAAM1|DBI|DCAF12|DCPS|DDAH1|DDIT3|DDX27|DDX39B|DDX3X|DDX56|DFFB|DHODH|DHRS7B|DHX15|DIP2A|DLGAP4|DNA2|DNAAF1|DNAJB9|DNAJC3|DNM3|DNMT1|DOLPP1|DONSON|DPY30|DPYSL2|DST|DSTN|DTNBP1|DTYMK|DUSP11|DUSP14|EBAG9|EEF1A1|EEF1D|EIF1|EIF2S1|EIF2S2|EIF4E|EIF4E2|EIF4G1|ELL|ELP5|EMD|ENO1|ENTPD5|EPHX1|EPS8|ERI1|ERRFI1|ESRRA|ETFB|ETFDH|ETV5|EXOC8|EXOG|EXOSC10|EXOSC2|EXOSC5|EXOSC6|EXT2|EZH1|EZH2|FAAP20|FADD|FAM134A|FAM151B|FAM210B|FAM46C|FAM49B|FAM60A|FANCA|FANCL|FASN|FBL|FBXL8|FBXO21|FBXO31|FBXO5|FBXO9|FBXW5|FDPS|FGFR1OP2|FIGN|FIP1L1|FKBP1A|FLYWCH1|FMR1|FOPNL|FOXO4|FRMD8|FXN|FZR1|G3BP2|GABRB1|GABRB2|GALNT11|GAPVD1|GCLC|GEMIN2|GEMIN4|GFPT1|GINM1|GLB1|GMEB1|GNB1L|GNG5|GNPAT|GPBP1L1|GPR19|GRHPR|GRSF1|GSG2|GSR|GSS|GSTZ1|GTF2A1|GTF2F1|GTF3A|GTF3C1|HAUS8|HBP1|HCRT|HDAC3|HDHD3|HEATR5B|HEXIM1|HIRIP3|HMGN1|HMHA1|HMMR|HNRNPA1|HNRNPU|HNRNPUL2|HOOK3|HP1BP3|HSD17B4|HSDL1|HSF1|HSP90AA1|HSPA14|HSPA8|HSPA9|HSPD1|HSPE1|ICMT|IDE|IGFLR1|ILVBL|IMMT|ING2|INPP5A|INSIG2|INTS5|INTS6|IP6K2|IRAK4|IRF3|ISOC1|ITGA1|JAK2|JMY|KANK3|KAT2A|KDM4A|KEAP1|KIF26A|KIF2A|KIF6|KIFC1|KLHDC3|KLHL22|KPNA1|KRR1|KRTCAP2|LAGE3|LAMTOR4|LARP7|LDHA|LFNG|LIPA|LIPT2|LLGL1|LPCAT3|LRRC20|LRWD1|LSM2|LSM4|LSM7|LSR|LSS|LYPLAL1|LYRM2|LYSMD3|MACF1|MAFG|MAP1LC3B|MAP2|MAST2|MBD3|MBD4|MCM2|MCM4|MCM6|MEAF6|MED20|MED28|MED9|MEF2A|MEGF8|METTL22|MEX3B|MGAT1|MGST3|MKRN1|MKS1|MLST8|MLX|MMP17|MOB3A|MOCS2|MORF4L2|MPG|MRPL12|MRPL36|MRPL49|MRPS18B|MRPS25|MRPS6|MSH2|MTCH2|MTFMT|MTHFD1|MVD|MXI1|MYCBP|MYCBP2|MYH10|NAA10|NAA38|NAA50|NADK|NAGK|NAMPT|NANP|NAP1L4|NCKIPSD|NDEL1|NDNL2|NDST2|NDUFA12|NDUFA3|NDUFA6|NDUFAF4|NDUFB10|NDUFB4|NDUFS6|NDUFS8|NEGR1|NEK4|NF1|NF2|NFX1|NIPA2|NOL7|NONO|NOTCH4|NPM1|NPRL3|NR1D2|NR2C2AP|NRTN|NT5C|NUBP1|NUDT16L1|NUDT7|NUP50|NUP62|NXT1|OAZ2|OCLN|ODC1|OIP5|OPA1|ORC4|ORC6|OSTC|OSTF1|P4HA1|PAIP2|PAPSS1|PARD6B|PARK2|PARK7|PATZ1|PCGF3|PCNA|PCNT|PDCD4|PDCD6|PDCD6IP|PDCL3|PER1|PFDN1|PGAM1|PGAM5|PGLS|PGP|PGRMC2|PHACTR4|PI4K2B|PIGH|PIGP|PIN4|PJA1|PLA2G12A|PLD1|PLEKHA3|PLEKHA8|PLEKHJ1|PMS1|PMS2|PNISR|PNMAL2|POFUT2|POLD1|POLE4|POLI|POLR1A|POLR2A|POLR2F|POLR2I|POLR3K|POM121|PPAN|PPCS|PPDPF|PPIF|PPP1CB|PPP1R10|PPP1R12A|PPP1R7|PPP2CA|PPP2R5C|PRDM4|PRDX3|PRDX5|PRKAR2A|PRKDC|PRMT1|PRPSAP1|PSMA3|PSPC1|PSTK|PTBP1|PTCH1|PTGES3|PTPN2|PTPRS|PTS|PUS3|PUS7L|PUSL1|PVR|PYGL|RAB10|RAB14|RAB32|RAB4A|RABEP1|RABGGTA|RABGGTB|RAE1|RAF1|RALBP1|RAN|RANBP1|RASSF1|RAVER1|RBBP4|RBM19|REEP6|RELL2|RENBP|RETSAT|REXO2|RGS19|RHOA|RHPN1|RIOK2|RNF145|RNF19B|RNF5|RNF8|RNGTT|RNH1|RNPS1|RPA1|RPA2|RPL13A|RPL14|RPL19|RPL36|RPP14|RPP30|RPS12|RPS13|RPS21|RPS29|RRP9|RXRB|S100A13|SAFB2|SARM1|SAYSD1|SBF2|SCAMP3|SCARB2|SCN3B|SDCBP|SDF4|SDHA|SDHB|SDR39U1|SERINC1|SERTAD1|SET|SETD4|SF3A2|SF3B1|SF3B2|SFI1|SFMBT1|SFT2D1|SGMS1|SGTA|SH3BGR|SHBG|SHMT1|SHPRH|SIKE1|SIRT5|SLC12A6|SLC25A10|SLC25A3|SLC27A3|</td></tr>
</table>

		SLC35G1 | *SLC39A6* | *SLC3A2* | *SLK* | *SLTM* | *SMAP2* | *SMARCA4* | *SMARCC1* | *SMARCD3* | *SMIM14* | *SMPD4* | *SMYD3* | *SNRK* | *SNRNP25* | *SNRPD2* | *SNW1* | *SOCS1* | *SORCS3* | *SOX12* | *SPC24* | *SPDL1* | *SPG11* | *SPOP* | *SPRY4* | *SRC* | *SRD5A1* | *SRR* | *SRSF1* | *SRSF10* | *SRSF11* | *SSB* | *SSH2* | *SSNA1* | *SSSCA1* | *STAP2* | *STARD7* | *STIM2* | *STK38* | *STRADA* | *STRN3* | *STUB1* | *STX17* | *STX3* | *SYCE2* | *SYP* | *SZRD1* | *TAF1B* | *TAF6* | *TARS* | *TAX1BP3* | *TBC1D8* | *TBCB* | *TBCD* | *TCEB2* | *TCF19* | *TCF25* | *TCP1* | *TCTA* | *TCTEX1D2* | *TDP1* | *TDRD7* | *TFE3* | *TGIF1* | *THAP7* | *THBS2* | *THOC3* | *THOP1* | *THRA* | *TIA1* | *TIMELESS* | *TIPARP* | *TMED9* | *TMEM116* | *TMEM141* | *TMEM14C* | *TMEM175* | *TMEM180* | *TMEM263* | *TMEM39A* | *TMEM43* | *TMEM65* | *TMEM9* | *TMTC2* | *TMX1* | *TNKS2* | *TOMM20* | *TOMM34* | *TPM1* | *TPRA1* | *TPX2* | *TRAF3IP1* | *TRAPPC9* | *TRIM28* | *TRIM62* | *TRIO* | *TRMT2A* | *TSEN2* | *TSEN34* | *TSPAN3* | *TSSC4* | *TTC7B* | *TTLL12* | *TTLL9* | *TUBA1B* | *TUBG1* | *TWF2* | *TXNL1* | *U2AF1* | *U2AF2* | *UBA2* | *UBA52* | *UBE2T* | *UBE4B* | *UCK1* | *UGGT2* | *UIMC1* | *UNG* | *UQCR11* | *UQCRB* | *UQCRFS1* | *USF1* | *USO1* | *USP21* | *UXS1* | *VAMP2* | *VAPA* | *VDAC2* | *VKORC1L1* | *VMAC* | *VPS28* | *WDHD1* | *WDR1* | *WDR43* | *WDR45* | *WDR62* | *WNT5A* | *WRB* | *XPO5* | *XRCC2* | *XRCC5* | *YARS2* | *YIF1A* | *YTHDF1* | *YWHAH* | *ZBTB17* | *ZBTB8OS* | *ZC3H14* | *ZDHHC12* | *ZFAND2A* | *ZFAND3* | *ZFYVE27* | *ZMYND19* | *ZNF652* | *ZNFX1* | *ZNHIT1* | *ZSCAN18* | *ZWINT*
Diethylhexyl phthalate	149	*ACADM* | *ACAP3* | *ACAT2* | *ACOT13* | *ACTB* | *AKAP17A* | *AKT2* | *ALAS1* | *ALDOA* | *AMZ2* | *ANAPC11* | *ARMCX2* | *ARRB2* | *ATG7* | *BCAT2* | *BROX* | *BSG* | *CASZ1* | *CBX2* | *CCNB1* | *CCNE1* | *CCT3* | *CCT6A* | *CDC34* | *CDV3* | *CEP85* | *CNKSR3* | *CSAD* | *CYCS* | *DBI* | *DECR2* | *DLGAP4* | *DNAJB9* | *DNMT1* | *DPY30* | *DPYSL2* | *DYNLL1* | *EIF4G1* | *ENTPD5* | *EPHX1* | *ERCC6* | *ERP29* | *ESRRA* | *ETV5* | *FAM102A* | *FASN* | *FBXL8* | *FBXO21* | *FBXO9* | *FDPS* | *FITM2* | *FKBP2* | *FMR1* | *G3BP2* | *GABRB1* | *GNPTG* | *GPR19* | *GRPEL2* | *GSR* | *GSTK1* | *GSTM3* | *HDAC2* | *HDAC4* | *HSD17B4* | *HSDL2* | *HSP90AA1* | *HSPA4L* | *HSPA8* | *HSPA9* | *HSPD1* | *HSPE1* | *INPP4A* | *INSIG2* | *IRS1* | *ITGA1* | *KIF18A* | *KLHDC3* | *LDHA* | *LRRC42* | *LSS* | *MAP3K14* | *MAPT* | *MECP2* | *MIDN* | *MMADHC* | *MMP17* | *MRPL54* | *MRPS18B* | *MSH2* | *MVD* | *NAE1* | *NDUFA3* | *NF1* | *NKTR* | *NOTCH4* | *NPM1* | *NR1D1* | *ODC1* | *ORC4* | *PAPSS1* | *PARK2* | *PDCD5* | *PDCD6* | *PDIA5* | *PER1* | *PGLS* | *PI4K2B* | *PIGP* | *PLD1* | *POLE4* | *POU2F1* | *PPP1R10* | *PPP2R5C* | *PSMA2* | *PSMA4* | *PSMC4* | *PTPRS* | *PUSL1* | *PYGL* | *RBX1* | *RETSAT* | *RNF14* | *RNF19A* | *RPL36* | *RPS13* | *RPS23* | *RPS6KB1* | *RUNX2* | *RXRB* | *SCN3B* | *SEC61G* | *SIAH2* | *SLC37A4* | *SMAP1* | *SNX7* | *SPRY4* | *SRD5A1* | *SRR* | *SYP* | *TIMM13* | *TIMM9* | *TMEM141* | *TPM1* | *TUBA1B* | *TUBB4B* | *TXNL1* | *UCK1* | *VDAC3* | *ZWINT*
Polychlorinated biphenylsCadmium	263	*ARAF* | *ARHGEF10* | *ARL3* | *AURKA* | *AURKB* | *BASP1* | *BCL10* | *C18ORF54* | *CASP2* | *CCNF* | *CDC42* | *CDK1* | *CDK2AP2* | *CDKAL1* | *CDT1* | *CENPN* | *CHTF18* | *CIRBP* | *CKS1B* | *CNTLN* | *CTPS1* | *DAD1* | *DIDO1* | *DNAJB9* | *DNMT1* | *DTYMK* | *EEF1D* | *EIF2S2* | *EPHX1* | *FAM98A* | *FANCA* | *FBXO5* | *FCHO2* | *GABRB2* | *GNPTAB* | *HNRNPU* | *HSPD1* | *KEAP1* | *KIF3A* | *KIF4A* | *LENG8* | *MACF1* | *MCM2* | *MCM6* | *P4HB* | *PCNA* | *PEX5L* | *PILRB* | *PLK4* | *PPIA* | *PPM1F* | *PPM1K* | *PPME1* | *PPP4R2* | *PRC1* | *PRRT2* | *RAD23B* | *RAF1* | *RBKS* | *RPA1* | *RTKN2* | *SAFB* | *SCML1* | *SDCBP* | *SFXN2* | *SGMS1* | *SMC4* | *SMG6* | *SPAG8* | *SPOCD1* | *TCF25* | *THRA* | *TMEM38B* | *TNKS2* | *TRPM7* | *TSEN54* | *TSPAN12* | *TXLNA* | *UBE2T* | *UNG* | *VDAC2* | *ZNF428* | *ZNF692* | *ZSCAN18* | *ZWINT* *A1BG* | *ACADM* | *ACP1* | *AIFM1* | *AKIRIN2* | *ALDOA* | *ALYREF* | *ANLN* | *ANXA5* | *ARF1* | *ARHGDIA* | *ASH1L* | *ATAT1* | *ATXN7L3B* | *AUH* | *AURKA* | *BAG1* | *BCAT1* | *BNIP3* | *BTF3* | *BUB1* | *CACYBP* | *CAPZA2* | *CCDC96* | *CCDC97* | *CCNB1* | *CCNE1* | *CCNF* | *CCNT2* | *CCT5* | *CD274* | *CD47* | *CDC42* | *CDCA2* | *CDK1* | *CDK2AP2* | *CDKN2B* | *CHD2* | *CIRBP* | *CISD2* | *CKS1B* | *CLEC11A* | *COX15* | *COX5B* | *COX6C* | *CREB1* | *CREM* | *CSAD* | *CTBP1* | *CTSD* | *CYB5R4* | *CYCS* | *CYHR1* | *D2HGDH* | *DBI* | *DCAF6* | *DDIT3* | *DEDD2* | *DHDDS* | *DLD* | *DNAJB6* | *DNAJB9* | *DNMT1* | *DST* | *DSTN* | *DTYMK* | *E2F1* | *EEF1A1* | *EEF1D* | *EEF1G* | *EID2* | *EIF2AK1* | *EIF4B* | *EIF5A* | *ENO1* | *EPHX1* | *EPT1* | *ERRFI1* | *ETV5* | *EXOSC2* | *FAM122B* | *FASN* | *FBXL5* | *FBXO5* | *FKBP1A* | *FRMD8* | *FSTL3* | *GAA* | *GABPB1* | *GCLC* | *GDPD1* | *GLRX3* | *GNAO1* | *GNPDA2* | *GNPTG* | *GNS* | *GPR37* | *GRHPR* | *GSR* | *GSS* | *GSTM3* | *GSTZ1* | *GTF3A* | *GTSE1* | *GXYLT1* | *HARS* | *HILPDA* | *HINT1* | *HNRNPA1* | *HNRNPH1* | *HSD17B4* | *HSDL2* | *HSF1* | *HSP90AA1* | *HSPA4L* | *HSPA8* | *HSPD1* | *HSPE1* | *INPP5K* | *INSIG2* | *INTS10* | *INTS5* | *IRS1* | *KIF4A* | *KLF13* | *LDHA* | *LRPAP1* | *LSM2* | *MAD2L1* | *MAGI1* | *MAP1LC3B* | *MAP2* | *MAP3K14* | *MARS2* | *MCM2* | *MCM4* | *MCM6* | *MED20* | *MED25* | *MED26* | *MESP1* | *MGST3* | *MINK1* | *MMP17* | *MPL* | *MSH2* | *MST1* | *MYH10* | *MYH14* | *NAA38* | *NDUFA8* | *NDUFS8* | *NEK2* | *NPRL3* | *NRN1* | *NRTN* | *NUDT16L1* | *NUP35* | *NUSAP1* | *OAZ2* | *OCLN* | *OIP5* | *OS9* | *OSTF1* | *PAICS* | *PAQR4* | *PARK2* | *PATZ1* | *PDCD2L* | *PELI1* | *PER1* | *PGLS* | *PGRMC2* | *PIGH* | *PINK1* | *PKMYT1* | *PPCS* | *PPIA* | *PPIF* | *PQLC2* | *PRC1* | *PTK2* | *PVR* | *RAB32* | *RAB4A* | *RABGGTA* | *RAC1* | *RAF1* | *RAN* | *RASSF1* | *RELA* | *RETSAT* | *RGS20* | *RHEBL1* | *RNF19B* | *RNF8* | *ROCK1* | *RPA2* | *RPL10* | *RPL13A* | *RPL14* | *RPS12* | *RPS6KB1* | *RPUSD1* | *RSRP1* | *RUNX2* | *SDHA* | *SDHB* | *SGMS1* | *SHBG* | *SHC1* | *SHMT2* | *SHPRH* | *SLC16A1* | *SLC30A4* | *SLC37A4* | *SLC39A14* | *SLC3A2* | *SMC4* | *SNAP23* | *SPTAN1* | *SRD5A1* | *SRPK1* | *SRR* | *SRRM1* | *SRSF11* | *STX2* | *TAF12* | *TAX1BP3* | *TBCB* |

<table>
<tr><td></td><td></td><td>TCEB2 | TCF19 | TCP1 | TDP2 | TFAP2B | THRA | TMX4 | TNFSF13 | TPM1 | TPX2 | TRADD | TRPM7 | TSSC4 | UBE2D3 | UBE2D4 | UBE2N | UCK1 | USF1 | USP39 | VGF | VHL | WDSUB1 | WWP1 | XPA | XPO5 | YWHAZ | ZBTB17 | ZBTB4 | ZFAND2A | ZMYND19 | ZNF286A | ZNF554 | ZNF747</td></tr>
<tr><td>Arsenic</td><td>520</td><td>ABCA3 | ABHD14A | ABLIM2 | ACAD9 | ACTB | ADAM10 | ADCK5 | ADCY9 | ADIPOR2 | AGPAT1 | AKAP8 | ALDOA | ANAPC16 | ANAPC7 | APBB3 | APC2 | APLF | APOLD1 | ARF1 | ARHGEF10 | ARHGEF18 | ARPC2 | ARSB | ASB7 | ASB8 | ATAD3B | ATF7IP | ATG10 | ATG2A | ATG7 | ATM | ATP11A | ATP6V0D1 | AURKA | B3GNTL1 | B9D2 | BBS9 | BCAR1 | BCL10 | BCL2L12 | BCL7B | BLOC1S2 | BORCS7 | BPNT1 | BTBD3 | BTBD7 | BYSL | C16ORF87 | C17ORF82 | C19ORF57 | C1GALT1C1 | C1ORF35 | C2CD3 | C6ORF136 | CASP2 | CASZ1 | CBFB | CBLL1 | CBR4 | CCDC114 | CCDC150 | CCDC47 | CCDC89 | CCHCR1 | CCNB1 | CCNE1 | CD27 | CD59 | CDC42 | CDC42BPB | CDK1 | CDK19 | CDKN2B | CDKN2D | CDYL | CEP104 | CEP250 | CEP63 | CEP72 | CES4A | CHCHD4 | CHD2 | CHORDC1 | CHST12 | CIPC | CLASP1 | CLN8 | CLP1 | CLPTM1L | CNOT1 | CNST | COG1 | COQ2 | COX6A2 | CPSF3L | CREB1 | CREM | CRTC1 | CSAD | CSE1L | CSNK1G2 | CTBP1 | CTSD | CXXC1 | CYCS | CYHR1 | D2HGDH | DAZAP1 | DCPS | DDIT3 | DDX10 | DDX11 | DDX27 | DDX39B | DECR2 | DENND3 | DFFB | DHDDS | DHODH | DIRC2 | DLGAP4 | DLL3 | DNA2 | DNAJC5 | DNM3 | DNMT1 | DOLPP1 | DUSP14 | DYNC1H1 | DYNLRB2 | DYRK1B | E2F1 | E2F6 | EED | EEF1G | EHD1 | EHMT1 | EIF5A | ELL | ENO1 | ENTPD6 | ERCC2 | ERCC6 | ERGIC3 | ERMAP | ERP29 | ERRFI1 | ETFB | ETFDH | EVI5 | EXOC8 | EXOSC5 | EZH2 | FAAP20 | FAM133B | FAM134C | FAM189A1 | FAM213A | FAM3A | FAM49B | FANCA | FASN | FBXL5 | FBXO31 | FCHO2 | FGFR1OP2 | FGGY | FIGN | FIP1L1 | FKBPL | FUT8 | FZR1 | GAK | GCLC | GDPD1 | GFOD2 | GJA9 | GLE1 | GMEB2 | GNAO1 | GNPDA2 | GOLGA4 | GPANK1 | GPR180 | GPRIN1 | GSR | GSS | GSTM3 | GTF2A1 | GTSE1 | HAUS3 | HCRT | HDAC4 | HDHD3 | HEXIM2 | HLCS | HNRNPA1 | HNRNPM | HP1BP3 | HRAS | HSD17B4 | HSDL1 | HSP90AA1 | HSPA8 | HSPA9 | HSPD1 | IBA57 | IFT122 | IFT80 | IGFBP3 | INPP4A | INPP5A | INSIG2 | INTS6 | IP6K2 | IQCE | IRAK4 | ISOC1 | ITFG2 | ITGAE | IWS1 | JAK2 | KANK3 | KANSL1 | KANSL1L | KDM5C | KEAP1 | KIF4A | KIF6 | KIFAP3 | KIFC1 | KLF13 | KPNA1 | KRR1 | LAP3 | LDHA | LENG8 | LIG3 | LMF1 | LPCAT1 | LRCH3 | LRRC20 | LRRC58 | LSM2 | LSM4 | LYPLA1 | LYRM2 | MACF1 | MAD2L1 | MAGI1 | MAGIX | MAP2 | MAPK7 | MAPT | MBD1 | MED18 | MED26 | MED9 | MEGF8 | METTL22 | MFSD9 | MGA | MMADHC | MMP17 | MMRN2 | MMS19 | MPG | MSH2 | MSH5 | MSRA | MST1 | MYH10 | MYH14 | MYH7B | MYO1D | N6AMT1 | NADK | NAP1L4 | NARFL | NARS2 | NCAN | NDEL1 | NEFH | NEFM | NEGR1 | NEIL2 | NEK4 | NEUROD1 | NF1 | NFYA | NKTR | NOMO2 | NOTCH4 | NPM1 | NPRL3 | NR1D2 | NRN1 | NRTN | NSUN2 | NUBP1 | OCLN | ORC4 | P4HA1 | P4HB | PAICS | PAIP2 | PARK2 | PATL1 | PCBP2 | PCDH9 | PCGF3 | PCIF1 | PCLO | PCNA | PCNT | PDCD2L | PDCD4 | PDE7A | PDE8A | PELI1 | PER1 | PGP | PGS1 | PHF20 | PHF23 | PI4K2B | PI4KA | PIN4 | PITHD1 | PKD1 | PKIB | PLCG1 | PLD1 | PLEKHA8 | PLEKHJ1 | PLOD3 | PLS1 | POFUT2 | POLD1 | POLR2H | POLR3B | POM121 | PPAN | PPAT | PPFIA3 | PPM1D | PPP1R10 | PPP2R1A | PPP2R5C | PPP5C | PPTC7 | PQLC2 | PRDX5 | PRKAR2A | PRKDC | PSMA3 | PTBP1 | PTCH1 | PTK2 | PTPRS | PVR | RABL6 | RAF1 | RALGAPA2 | RAPGEF6 | RASSF1 | RBM12B | RBM19 | RBM4B | REEP3 | RGS17 | RHEBL1 | RNF130 | RNF14 | RNF149 | RNF170 | RNF19A | RNF8 | RNPS1 | ROCK1 | RPA1 | RPL36 | RPUSD3 | RRM2B | RSRP1 | RUNX2 | RXRB | S100A13 | SBF1 | SBF2 | SCAP | SCN3B | SDHA | SDHB | SET | SGMS1 | SHMT1 | SIAH2 | SIL1 | SIVA1 | SKA1 | SLC30A5 | SLC3A2 | SLC44A2 | SLC9A3 | SMAD4 | SMARCA4 | SMARCC1 | SMPD4 | SMYD3 | SNX32 | SNX8 | SOS1 | SOS2 | SPATA5L1 | SPDL1 | SPG7 | SPICE1 | SPPL2B | SRC | SRP68 | SRRM2 | SRSF11 | SSH1 | ST5 | STARD7 | STIM2 | STRN4 | STX12 | STX1A | STX3 | SUCLA2 | SUFU | SYCE2 | SYPL1 | TAF4 | TAF6 | TAF7 | TARBP1 | TBC1D24 | TBCD | TECR | TEX264 | TFDP1 | TIGD1 | TIMM8B | TMEM106C | TMEM115 | TMEM116 | TMEM135 | TMEM263 | TMTC2 | TOLLIP | TOX2 | TPCN2 | TPGS1 | TPMT | TRAPPC9 | TRIM39 | TRIT1 | TSEN54 | TSPAN12 | TSPAN14 | TSPYL5 | TTC37 | TTLL12 | TUBB | TUBB4B | U2AF2 | UBA52 | UBE2D4 | UBE2K | UBE2N | UBE2V1 | UGGT2 | UNKL | UPF1 | UQCRC1 | USO1 | UVSSA | VAPA | VARS2 | VASH1 | VHL | VPS13C | VPS26B | VPS8 | WARS2 | WDFY2 | WDR19 | WDR20 | WDR5 | WRB | XPA | XRCC1 | XRCC5 | YIPF3 | ZBTB4 | ZBTB49 | ZC3H3 | ZFAND3 | ZFR | ZNF10 | ZNF146 | ZNF2 | ZNF248 | ZNF324B | ZNF331 | ZNF445 | ZNF524 | ZNF527 | ZNF551 | ZNF610 | ZNF611 | ZNF692 | ZNF771 | ZNF782 | ZNF787 | ZNF845 | ZP3</td></tr>
<tr><td>Manganese</td><td>50</td><td>ACAT2 | ARHGDIA | ATM | BNIP3 | BUB1 | CKS2 | COMMD8 | CREB1 | CYCS | CYHR1 | DDAH1 | DDIT3 | DLD | DNAH14 | ENO1 | EPHX1 | ERCC2 | ERCC6L | ERICH1 | GSR | HSPA8 | IPO11 | MAGED2 | MAP3K14 | MCM4 | NAMPT | NOTCH4 | P4HB | PARK2 | PARK7 | PPM1D | PSMA3 | PSMA4 | PVR | RAB23 | REEP4 | RELA | RNF145 | SCN3B | SDCBP2 | SEH1L | SLC39A14 | SMIM14 | SPTAN1 | SRC | TFAP2B | TRPM7 | UBE2N | VPS13A | ZMYM6</td></tr>
</table>
